# Synthesis and Morphology Characteristics of New Highly Branched Polycaprolactone PCL

**DOI:** 10.3390/molecules29050991

**Published:** 2024-02-24

**Authors:** Aleksandra Zioło, Beata Mossety-Leszczak, Małgorzata Walczak, Beata Strachota, Adam Strachota, Kamil Awsiuk, Natalia Janiszewska, Joanna Raczkowska

**Affiliations:** 1 Doctoral School of the Rzeszów University of Technology, al. Powstańców Warszawy 12, 35-959 Rzeszow, Poland; 2 Department of Industrial and Materials Chemistry, Faculty of Chemistry, Rzeszow University of Technology, al. Powstańców Warszawy 12, 35-959 Rzeszow, Poland; mossety@prz.edu.pl (B.M.-L.); mwalczak@prz.edu.pl (M.W.); 3 Institute of Macromolecular Chemistry, Czech Academy of Sciences, Heyrovského nám. 2, 162 00 Praha, Czech Republic; beata@imc.cas.cz (B.S.);; 4 M. Smoluchowski Institute of Physics, Faculty of Physics, Astronomy and Applied Computer Science, Jagiellonian University, Łojasiewicza 11, 30-348 Krakow, Polandnatalia.janiszewska@doctoral.uj.edu.pl (N.J.);; 5 Doctoral School of Exact and Natural Sciences, Jagiellonian University, Łojasiewicza 11, 30-348 Krakow, Poland

**Keywords:** highly branched polymers, star polymers, poly(caprolactone), biodegradable polymers, core–shell macromolecules, dendrimers, macro-initiators of polymerization

## Abstract

A simple and efficient method for the synthesis of biodegradable, highly branched polycaprolactone (PCL) is presented. The solvent-free (bulk) reaction was carried out via ring opening polymerization (ROP), catalyzed by tin octanoate Sn(Oct)_2_, and it employed hyperbranched polyamide (HPPA) as a macro-initiator. The core–shell structure of the obtained products (PCL-HPPA), with the hyperbranched HPPA core and linear PCL chains as shell, was in the focus of the product characterization. ^1^H nuclear magnetic resonance (^1^H NMR) and elemental analysis confirmed the covalent incorporation of the HPPA in the products, as well as a high degree of grafting conversion of its amino functional groups. Confocal Raman Micro spectroscopy, and especially Time-of-Flight Secondary Ion Mass Spectrometry, further supported the existence of a core–shell structure in the products. Direct observation of macromolecules by means of cryogenic transmission electron microscopy, as well as gel permeation chromatography (GPC), suggested the existence of a minor ‘aggregated’ product fraction with multiple HPPA cores, which was attributed to transesterification reactions. Differential scanning calorimetry, as well as X-ray diffraction, demonstrated that the PCL-HPPA polymers displayed a similar degree of crystallinity to linear neat PCL, but that the branched products possessed smaller and less regular crystallites.

## 1. Introduction

Highly branched polymers (HP) have attracted considerable attention because of their unique physical and chemical properties such as high solubility, low viscosity in the molten state, low crystallinity and high end-group concentration, as compared to linear polymers. However, because of very limited possibilities of entanglement of their macromolecules, the branched polymers have poor mechanical properties. Attaching linear polymer chains as the outermost layer of the hyperbranched structure makes entanglement possible and thus improves the mechanical properties of such HPs [1,2].

In recent years, researchers have focused on developing new synthesis techniques for obtaining hyperbranched polymers in order to tailor their structure and properties. For this purpose, the range of monomers and functional groups that can be incorporated into hyperbranched polymers was greatly expanded. Recent research focuses on techniques achieving precise control of the hyperbranched structure, and on the relationship between the obtained structure and the physico-chemical and mechanical material properties. Structural parameters for branched polymers are referred to the degree of branching which defines the proportion of the number of dendritic units in a polymer to all the units (terminal, linear, dendritic); this can be calculated based on an NMR spectrum. Degree of branching can be also determined based on the fractional conversion of initial polymerization unit [1,3]. Poly(amidoamine) PAMAM dendrimers are one of the most popular objects of interest in this field of research. They are synthesized through a stepwise growth process, resulting in a highly branched structure with a central core and multiple dendritic branches with terminal amino groups [4]. The numerous functional groups on the surface of the PAMAM dendrimers can also be relatively easily converted into various different ones, thus allowing for additional tailoring of properties. Promising applications include drug delivery, imaging or nanotechnology [5,6,7]. Hyperbranched polyesters are an attractive polymer class with promising application potential. They can be synthesized via the polymerization of multifunctional monomers, so that a wide range of hyperbranched polyester varieties can be obtained: ABn-type hyperbranched polyesters, AB/AB2-type hyperbranched polyesters and polyesteramide hyperbranched polymers [8,9,10,11]. The AB2-type hyperbranched polyester Boltorn^®^ (with branches growing on a trialcohol core unit) is even offered as a commercial product, with alcohol groups on surface (standard) or with modified surface groups [12]. Such a functionalization allows hyperbranched polyesters to find applications in coatings, adhesives, biomedical materials or in nanotechnology.

Polycaprolactone (PCL) belongs to the biodegradable linear polyesters. It has received considerable research interest due to its unique properties, such as its mentioned biodegradability, biocompatibility and miscibility with a range of other polymers. The degree of crystallinity and the molecular weight of PCL, which are controlled by the synthesis conditions, are important factors in its thermal and mechanical properties [13,14]. The miscibility of PCL allows for the preparation of a variety of composites with new properties, customized to the planned usage. An example of biodegradable composites is polycaprolactone with bamboo powder. The addition of bamboo powder influenced mechanical, thermal and biodegradability [15]. Another example can be a co-continuous calcium phosphate/polycaprolactone composite used in bone scaffolds. Polycaprolactone is an important polymer in scaffolds, and the ability to produce composites increases its importance in this field [16]. Branched varieties, as well as PCL, have been extensively studied, which contain additional structural units such as glycidol, lactide, pentaerythritol or poly(ethylene glycol) [13,17,18,19]. However, only few studies to date have shown interest in highly branched PCL obtained by chain growth from a HP core. In these rare works, the tested HP cores consisted of hyperbranched polyesters, hyperbranched polyamidoamines or hyperbranched polyethers [20,21].

One of the most preferred routes to PCL is the ring opening polymerization (ROP) of ε-caprolactone (CL) with an initiator. An innovative approach is the use of hyperbranched polymers (HP) as multi-functional caprolactone polymerization initiators instead of mono-functional ones [21]. This approach yields a highly branched PCL polymer, with HP as a core and PCL as numerous linear arms.

In the present work, hyperbranched polyamide (HPPA) was employed as macro-initiator of ring-opening polymerization of ε-caprolactone (CL) in order to obtain branched PCL with HPPA core. Polymers with varied mass ratios macro-initiator/monomer were prepared, and their molecular parameters, morphology and thermal properties were characterized.

## 2. Results

### 2.1. Synthesis and Basic Characterization of the Highly Branched PCL Polymers

**Figure 1:**
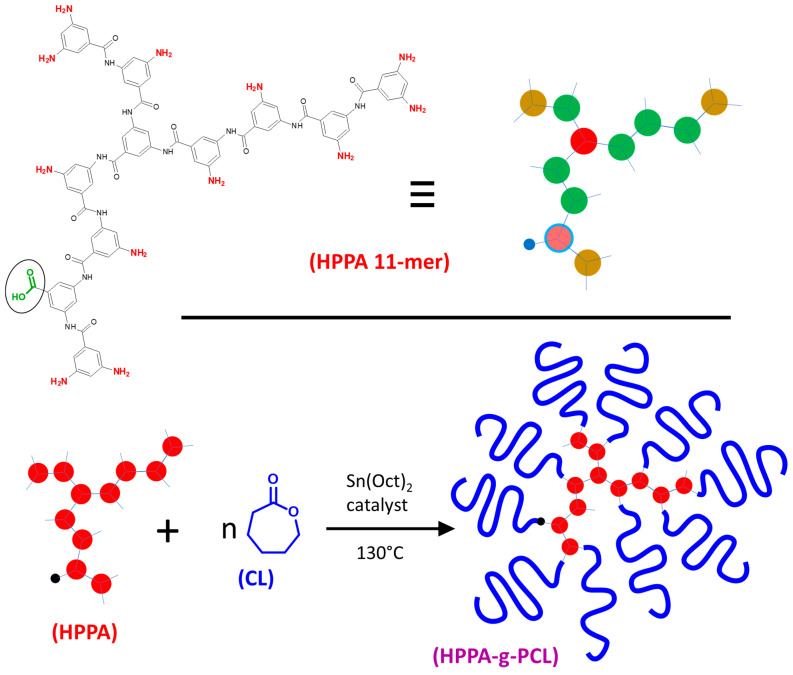
Synthesis of HPPA-PCL copolymers: Top: the HPPA macro-initiator core: structure of an average molecule, as well as its symbolic representation (brown spheres symbolize terminal repeat units, green spheres linear bonding ones, the red one is a branching unit, while the pink sphere with turquoise border is a starting unit in branching configuration which contains a lonely COOH group represented as a small turquoise sphere; the structure of the macro-initiator is discussed in detail in the Supplementary Information File). Bottom: grafting of caprolactone (CL) onto HPPA (oligomer made of red spheres), with PCL chains (blue color) starting from 84% of amino groups and also from the lonely COOH group of HPPA.

Highly branched polycaprolactone (PCL) polymers were synthesized according to the reaction depicted in Figure 1, starting from ε-caprolactone (CL) monomer and using the hyperbranched polymeric polyamide (HPPA, based on the 3,5-diaminobenzoic acid monomer, and previously described by the authors in [22]) as aromatic-amine-polyfunctional macro-initiator. Figure 1 shows an HPPA macro-initiator molecule close to the numeric average one (10.5-mer, *M* = 1428.216 g/mol, 11.5 amino groups and one COOH group, 1.3 branching units per molecule), as well as its grafting reaction with CL, which yields a highly branched PCL-HPPA structure (82% of the amino groups, and also the carboxyl are grafted). The characterization of the primary structure of the copolymer is discussed further below (The analysis of the primary structure of the macro-initiator is extensively analyzed in the Supplementary Information File, Section S3). The amine-initiated polymerization was catalyzed by tin dioctanoate (SnOct_2_). A reference sample of PCL (“PCL-linear”) was prepared at the same conditions, but without the aromatic-amine-polyfunctional macro-initiator (traces of moisture served as the nucleophilic initiator in this latter case). All the prepared PCL products were purified by dissolution/precipitation. The synthesis procedure is described in detail in the Experimental section.

The synthesized products were named according to the amount of the macro-initiator HPPA in the reaction mixture (0.033, 0.067, 0.13 and 0.33 wt.%), e.g., “PCL-0.033% HPPA”. The structure and molecular masses of the prepared highly branched PCL polymers, the proof of the incorporation of the hyperbranched HPPA core, the products’ phase behavior and the shape and size of the PCL macromolecules are discussed further below.

#### 2.1.1. Primary Structure Analyzed by Means of ^1^H NMR and FTIR Spectroscopy

**Figure 2:**
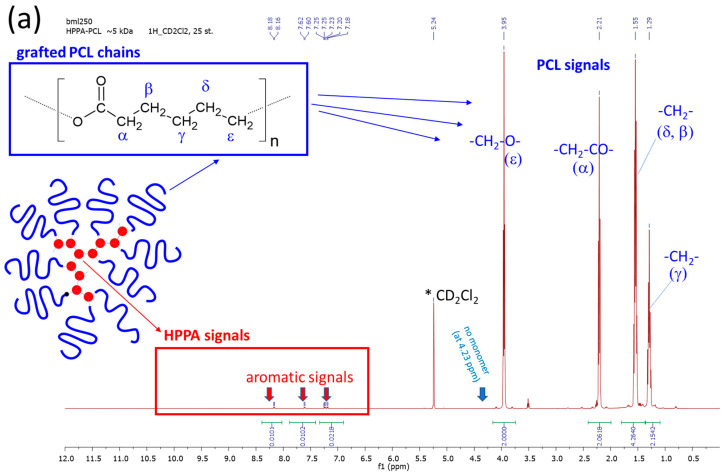

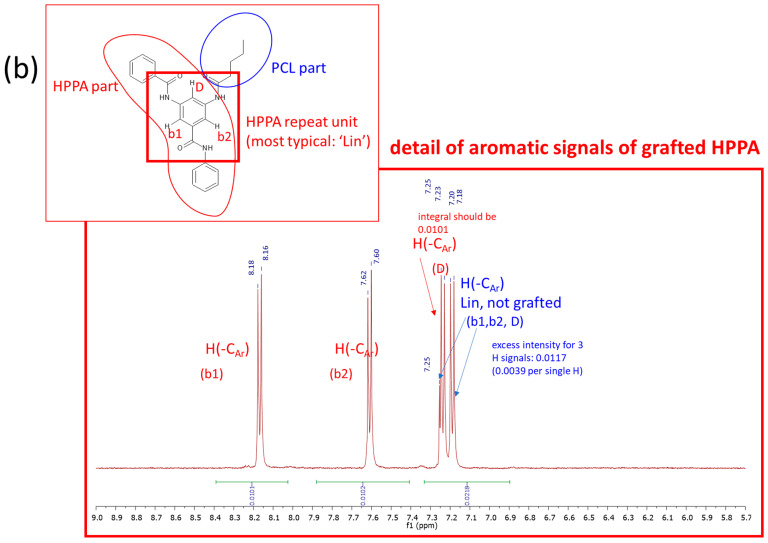
PCL-0.33% HPPA—5 kDa: ^1^H NMR spectrum (**a**) standard zoom; (**b**) highly amplified aromatic region. * CD_2_Cl_2_—Deuterated dichloromethane—solution for analysis.

The primary structure of the highly branched polycaprolactone products synthesized in this work was characterized by means of ^1^H NMR- and of ^13^C NMR-spectroscopy (the ^13^C spectra are shown in the Supplementary Information File). As example, the ^1^H NMR spectrum of the low-molecular-mass product PCL-0.33% HPPA—5 kDa is shown in Figure 2. The spectra of the remaining products can be found in the Supplementary Information File, Figures S20–S24. The spectra of all the products displayed the typical PCL signals at 3.99 ppm (-CH_2_CH_2_OC(O)-), 2.25 ppm (-CH_2_CH_2_COO-), 1.55 ppm (-CH_2_CH_2_COO-) and 1.32 ppm (-CH_2_CH_2_CH_2_CH_2_CH_2_-). The similarly positioned but somewhat shifted peaks of the CL monomer (especially the ones at 4.17 and 2.60 ppm, see spectrum in Figure S25) were absent. The peaks at 5.24 ppm and 1.0 ppm originate from CD_2_Cl_2_ (residual protons in this solvent) and tetramethyl silane (added as reference), respectively.

**Figure 3:**
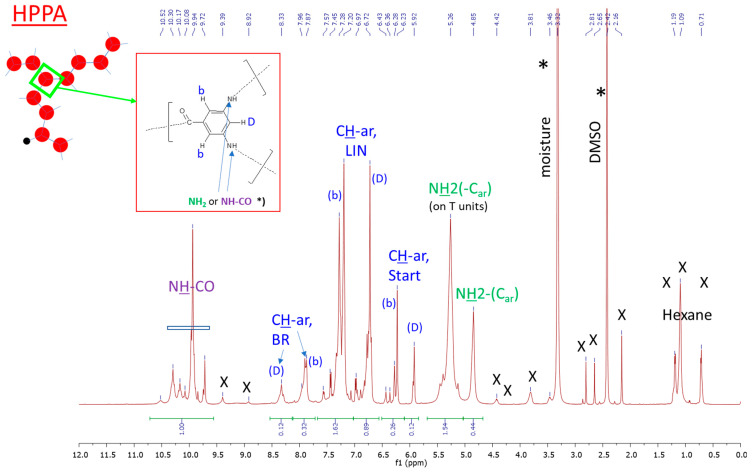
HPPA initiator: ^1^H NMR spectrum in DMSO at 80 °C; impurity signals are marked with “X”. * is described in a figure as moisture and DMSO.

The aromatic signals of the HPPA core (expected content: 0.33 wt.%) were too weak to be observed at the standard zoom of the ^1^H NMR spectrum, as seen in Figure 2a. If the spectrum is highly amplified (Figure 2b), however, weak aromatic signals at 8.17, 7.61, 7.24 and 7.19 ppm can be observed, which could be well correlated with the aromatic signals of HPPA highly grafted with PCL (see comparison with simulated spectrum in Figure S18), namely of a grafted originally linear HPPA unit (the linear unit is most frequent in HPPA). In Figure 3, the assigned ^1^H NMR spectrum of the non-grafted HPPA core is shown for comparison (recorded at different conditions, in DMSO-d6 at 80 °C). It can be seen that the aromatic signals of the intact HPPA initiator practically disappeared in Figure 2b, except for the region 7.25–7.18 ppm, where residues of the 7.28, 7.20 and of the up-shifted 6.72 ppm overlap with the signals of grafted HPPA. This can be recognized because the peak group at 7.25–7.18 ppm (assigned to H(-C_ar_)(D)) in the copolymer spectrum (Figure 2b) displays a higher integral than the theoretical one (0.0218 instead of 0.0102), and also some additional splitting. The excess intensity can be mainly attributed to non-grafted linear units of HPPA (as they generate the most intense peak in neat HPPA). However, the branching (and hence non-graftable) units of grafted HPPA (shifted in comparison to non-grafted HPPA spectrum) also must be hidden in this intensity excess. According to the analysis of the branching degree of the employed HPPA (see Supplementary Information File, Section S3. Initiator macromolecule: its molecular structure and degree of branching”), ca. 12% of the HPPA repeat units must be the branching ones. Hence, the excess aromatic peak intensity, which amounts to 28% of the total aromatic peak intensity, must be diminished by these 12% to obtain the intensity of graftable (mainly linear) repeat units which stayed non-grafted, thus yielding the value of 16% of aromatic intensity for these units. The conversion degree of the amine groups can hence be evaluated by comparing the intensity of the non-grafted units with that of the graftable ones (both grafted and non-grafted), thus yielding 16/(16 + 72), which corresponds to 82%: in the case of 10-mer, ca. 9 out of 11 amino groups are grafted. The details of the evaluation of the grafting degree via ^1^H NMR can be found in Supplementary Information File, Section S4. PCL-HPPA copolymer: NMR Analysis of its structure”. In the same Supplementary Section, the grafting analysis by ^13^C-NMR also is discussed. The aromatic peaks could be evaluated only qualitatively, but the spectra confirm a considerable conversion to grafted HPPA with some NH_2_ groups unreacted.

The length of the grafted PCL chains was also possible to evaluate, namely from the ratio of the integrals

-CH_2_-O-(PCL)/3 CH_ar_(HPPA) = 2.00/[(2/3) × (0.0102 + 0.0101 + est. 0.1015)] (from Figure 2a; the factor 2/3 is for normalization). From the ratio of 98.52, it follows that ca. 99 PCL repeat units occur per one per HPPA repeat unit. an HPPA 10-mer molecule has 11 amino groups (*P*_n_ + 1, see Supplementary Information File, Section S1. Possible structures of the initiator molecule”), of which 82% (9.02 groups) are grafted (according to ^1^H-NMR), as well as one graftable COOH group which is reactive and not sterically shielded and hence grafted. This means that on an average HPPA 10-mer, there are 10 PCL-grafting sites: 9 amino groups and 1 COOH group. The CL/HPPA monomers ratio hence also corresponds to the PCL chain length: 99 units per PCL chain in the sample “PCL-0.33% HPPA—5 kDa”, which would correspond to the mass fraction of 1.16% of HPPA and to a mass of 120.7 kDa. This molecular mass and the PCL content appears very high, if the GPC-determined molecular mass (5 kDa) is considered. Also, the elemental analysis (nitrogen) suggests a ca. 6-fold higher HPPA content, hence ca. 16 PCL units per chain and *M_n_* = 20.2 kDa. A reason for the discrepancy might be the worse relaxation of the polyaromatic HPPA core in the NMR analyses, which reduces its intensity. The GPC molecular mass of the relatively small, branched molecule can be under-estimated, on the other hand.

It can be summarized that the ^1^H-NMR results confirm the purity of the obtained PCL products (no residual monomer), and also indicate the presence of the highly grafted (82%) HPPA core in the products, which, however, can be proven only at high HPPA contents. The change in the position of aromatic HPPA peaks both in ^1^H and ^13^C NMR also confirms its grafting. Additional proofs of the covalent incorporation of the HPPA core are discussed further below (see Section 2.2).

The purity of the synthesized PCL products also was verified by means of FTIR spectroscopy. The spectra of all products, including the exemplary one PCL-0.33% HPPA, are presented in the Supplementary Information File (Figures S26–S30). The FTIR spectra of the prepared PCL products correspond to known spectra of linear PCL (see, e.g., [23], or Figure S26, PCL-linear). The peak at 3440 cm^−1^ is expectedly small: it corresponds to the stretching vibration of residual water (moisture) in KBr and of hydroxyl groups in PCL chain ends. The stretching vibrations of the CH_3_ and CH_2_ groups of PCL are prominent at 2946 cm^−1^ and 2866 cm^−1^, as well as the carbonyl peak of the PCL ester groups at 1725 cm^−1^. The characteristic peak of the bending vibration of CH_3_ and CH_2_ in PCL at 1471 cm^−1^ also clearly can be recognized. On the other hand, the fairly intense FTIR peak at ca. 1600 cm^−1^ (see FTIR of monomer in Figure S31), which is characteristic for the CL monomer but absent in PCL, was not observed in any of the obtained PCL products. Eventual FTIR peaks of the HPPA core could not be observed, which was in agreement with expectation, as its content was 0.33 wt.% or less.

#### 2.1.2. Nitrogen from HPPA Proven by Elemental Analysis

The presence of the HPPA core dendrimer in the prepared PCL products was also verified by means of elemental analysis, as the above-discussed proof by ^1^H NMR was close to the detection limit. As will be discussed below, the elemental analysis, namely the nitrogen content (HPPA contains nitrogen in contrast to PCL) determined for the sample PCL-0.33% HPPA, provided further evidence of the incorporation of the HPPA dendrimers into the PCL products (Table 1), and it also helped to more accurately assess the PCL/HPPB ratio (without NMR relaxation effects). The contents of carbon and hydrogen were also determined. The PCL-0.33% HPPA polymer after the standard reaction time of 4 h (and practically complete monomer conversion) achieved the molecular mass (GPC) of approximately 35 kDa (see Section 2.1.3 further below). Because of the low percentage of HPPA (in which nitrogen accounts for just 18.25 wt.%) in the sample, an analogous sample was prepared with much shorter PCL chains, “PCL-0.33% HPPA—5 kDa”, whose synthesis was interrupted after 30 min of reaction time and which achieved a molecular mass of approximately 5 kDa (according to GPC). This latter sample also was used for the above discussed structure analyses via NMR.

In the case of the standard SAMPLE “PCL-0.33% HPPA—35 kDa”, the percentage of nitrogen in the polymer was just on the threshold of the sensitivity of the analysis (0.26% N, see Table 1; sensitivity: <0.1%). Therefore, the analysis of the ‘small polymer’ with the molecular mass of 5 kDa was of great interest in order to confirm the presence and evaluate the content of HPPA more reliably. The analysis results indeed confirmed the presence of nitrogen and hence of the HPPA core in the product “PCL-0.33% HPPA—5 kDa” (namely 1.26% of N, which was distinctly above the analysis threshold). The increase in nitrogen content in the sample “PCL-0.33% HPPA—5 kDa” sample correlates well with its reduced molecular mass (which was achieved by shorter PCL chains). Additional proofs of the covalent incorporation of the HPPA core are discussed further below (see Section 2.2). If the contents of nitrogen are recalculated to the contents of HPPA and finally to monomer ratios PCL/HPPA and molecular masses (with 10.5-mer core), we obtain, for “PCL-0.33% HPPA—35 kDa”, a PCL chain length of 81 units, *M_n_* = 98.5 kDa (vs. 35 kDa from GPC) and an HPPA content of 1.42% (vs. nominal 0.33% at synthesis). In the case of “PCL-0.33% HPPA—5 kDa”, we obtain 15.7 PCL/chain, *M_n_* = 20.2 kDa (vs. 5 kDa in GPC) and an HPPA content of 6.92% (vs. nominal 0.33%). The molecular mass from GPC is under-estimated 4.0 times for the “5 kDa” sample and 2.8 times for “35 kDa”, if the elemental analysis results, combined with MALDI-TOF analysis of the HPPA core ([22] and also in Supplementary Information File), are considered more reliable. The under-estimation factor of GPC decreases with increasing length of the PCL chains grafted on the HPPA 10.5-mers, which is an expected trend. Unfortunately, the copolymers with low HPPA content could not be reliably analyzed concerning their N-content.

#### 2.1.3. Molecular Masses and Their Distribution

**Figure 4:**
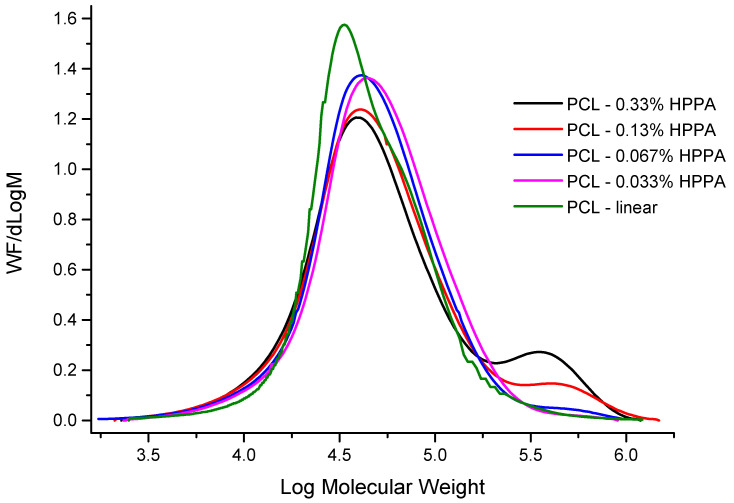
Molecular weight distribution curves of the synthesized PCL.

The molecular masses, as well as their distributions, for the obtained highly branched PCL polymers were characterized by means of gel permeation chromatography (GPC). Figure 4 compares the molecular weight distribution curves of the studied PCL polymers, while Table 2 summarizes the determined molecular masses and the dispersity indices (*M_w_*/*M_n_*). In view of the trends in the above-discussed elemental analysis data, and of the molecular masses measured by GPC, it can be estimated that the real molecular masses of the highly branched copolymers are ca. 2–3 times higher than the ones determined by GPC.

It can be seen in Figure 4 that the GPC curve of the PCL product obtained with 0.033 wt.% of macroinitiator (HPPA) showed a single peak. With 0.067 wt.% of HPPA, a very small second higher-molecular mass peak can be observed, while the curves of the polymer initiated with 0.13 and 0.33 wt.% of HPPA displayed already well-visible bimodality (highest in the case of 0.33 wt.% HPPA). This means that there are fractions with distinctly different molecular masses, which is also the reason why dispersity higher than 2 is observed with 0.13 and 0.33 wt.% of HPPA (the constituent peaks themselves are not significantly wider than in the case of 0.033 wt.% of HPPA).

**Scheme 1:**
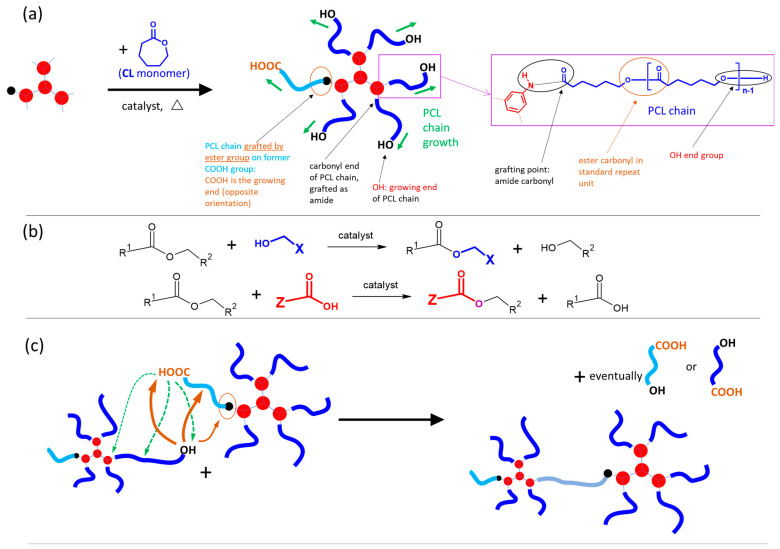

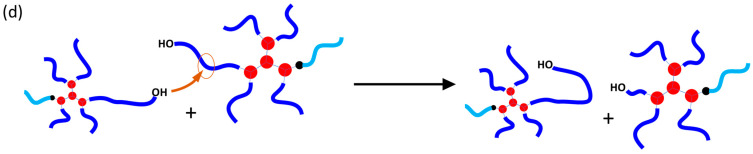
Side reactions: transesterification: (**a**) chain growth of PCL grafting on HPPA and important reactive sites; (**b**) transesterification of an ester with an alcohol or with an acid; (**c**) condensation of two branched molecules via the attack of an OH-terminated growing PCL chain on an COOH-terminated one, or vice versa; (**d**) exemplary chain transfer reaction (OH-terminated chains; COOH-terminated ones undergo analogous transfer reactions); the chain transfer can also occur intra-molecularly between two PCL chains on one molecule or on one chain alone (back-biting: elimination of a PCL cycle + shortening of the growing chain).

In Figure 4 and Table 2, it can be seen that the molecular mass of the linear PCL polymer, whose polymerization was initiated by residual moisture and catalyzed by the same catalyst as the highly branched PCL products, is somewhat lower (29 kDa vs. 34–37 kDa) than the molecular mass of the highly branched PCL products. The linear PCL also has a significantly lower dispersity index. In the highly branched PCL products, the numeric average molecular mass practically does not depend on the macro-initiator concentration, while the mass average molecular mass visibly grows between the HPPA contents of 0.067 and 0.33 wt.%. The bimodal distribution could be explained by a varying degree of branching of the PCL products with higher HPPA content, namely by occasional HPPA–HPPA condensation to larger cores, or more often by incorporation of another HPPA unit into a PCL chain growing from a ‘central’ HPPA core via transesterification reactions (see Scheme 1). These side reactions likely also could be catalyzed by the employed tin octanoate catalyst. 

Transesterification reactions are known to occur during or after ring-opening polymerization and broaden the molecular weight distribution [24]. The most likely transesterification reactions which can be expected to occur in the studied system are presented in Scheme 1: The reaction type shown in Scheme 1c leads to the covalent connection of two branched copolymer molecules, while the one in Scheme 1d just increases polydispersity. Both types also can generate free PCL chains. Considering the mentioned side reactions and the characteristics of GPC curves, the chemical heterogeneity of polymers can be confirmed, but the fraction of simple products is still dominant. Aggregated macromolecule structures (which could form according to Scheme 1c) were indeed observed by cryogenic transmission electron microscopy (Cryo-TEM), see further below (Section 2.3.1). Cryo-TEM and dynamic light scattering (DLS) were additionally used to assess the size of the macromolecules of the obtained PCL products (see further below, Section 2.3.1 and Section 2.3.2).

If referring to literature, it can be seen that the development of molecular mass distribution in the case of using macro-initiators is relatively complex. Additional proofs of the covalent incorporation of the HPPA core are discussed further below (see Section 2.2) [25,26].

The molecular weight distribution curve (GPC) of the HPPA macro-initiator in DMSO (not in THF, in contrast to the copolymers) is presented in the Supplementary Information File (Figure S4): the peaks appear between log*M* values of 2.2 and 3.6.

### 2.2. Proving the Covalent Incorporation of the HPPA Initiator into the PCL Structure

An important issue during the characterization of the prepared highly branched PCL products was to confirm that the HPPA macro-initiator was covalently incorporated into the polymer structure. Supporting analysis results were obtained, such as the observation of weak aromatic signals at markedly shifted positions (due to grafting) in NMR spectra, or the observed presence of nitrogen in the polymer structure (elemental analysis). But even in the case of the sample with the highest HPPA content (0.33 wt.%), both analyses were close to their detection limits. At so low, and especially at lower, HPPA contents, the presence of this macro-initiator (or any detail about its bonding situation) is almost impossible to determine using known techniques. The best-quality analysis results were obtained if the synthesis of the sample PCL-0.33% HPPA was quenched after a short polymerization time, so that a molecular weight of approximately 5 kDa (GPC) was achieved instead of 35 kDa (GPC) and the real HPPA content was raised ca. 6 times.

Several additional analyses were performed in order to prove more directly the covalent incorporation of HPPA into the prepared PCL products: the kinetics of the polymerization of the CL monomer (^1^H NMR) were recorded for all the HPPA-initiated systems, as well as for the neat PCL system. A distinct acceleration was observed in the case of the addition of increasing amounts of HPPA as macro-initiator. Additionally, Time-of-Flight Secondary Ion Mass Spectrometry (ToF-SIMS) was employed to detect structural fragments containing nitrogen and carbon, while Raman spectroscopy was used to detect characteristic C-N bond vibrations. Both the latter methods indicated that a nitrogen-containing core is covered by a nitrogen-free (PCL) shell: significant signals with 5 kDa vs. very weak signals with 35 kDa.

#### 2.2.1. Kinetics of the HPPA-Initiated Polymerization

**Figure 5:**
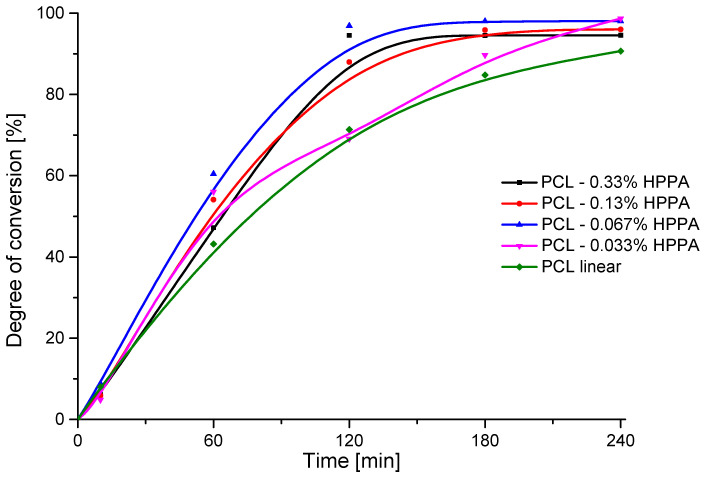
Kinetic curves for PCL with addition of hyperbranched polymer as an initiator and PCL obtained without any initiator.

The kinetics curves of the polymerization of the CL monomer (determined using ^1^H NMR) for the different studied HPPA-initiated PCL polymers, as well as for the reference sample PCL-linear (initiated by moisture traces), are depicted in Figure 5.

The reaction of PCL without macro-initiator (PCL-linear) is distinctly slower, and a conversion slightly above 90% is reached only after 4 h of reaction (at 130 °C, details: see Experimental section). In the case of PCL synthesis using 0.067% to 0.33% of the HPPA macro-initiator, the same conversion (90%) is reached already after 2 h. In the case of 0.033 wt.% of HPPA, 3 h are needed to achieve 90% conversion. All the HPPA-initiated syntheses achieve practically quantitative conversion after 4 h, so that this reaction time was used as the standard one in all the preparative reactions in this study. The kinetics study clearly proves that at the employed synthesis conditions, the polymerization of the CL monomer started by the HPPA macro-initiator is distinctly faster than CL-self-polymerization. Hence, if HPPA is present in the studied PCL samples, it can be expected to be covalently incorporated, due to the observed initiating activity.

#### 2.2.2. Core-Shell Structure and Nitrogen Presence Evaluated by ToF—SIMS

**Figure 6:**
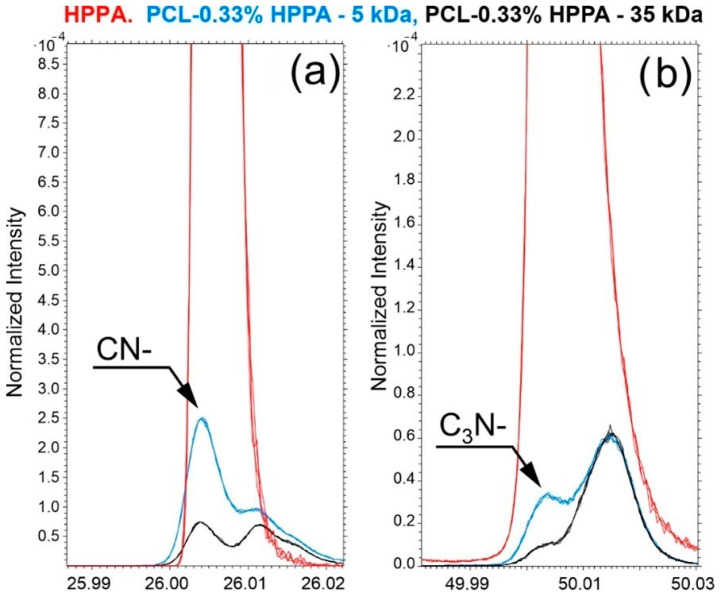
High mass resolution spectra of (**a**) CN- (*m*/*z* = 26.003) and (**b**) C_3_N- (*m*/*z* = 50.003) collected for thin films of (red) HPPA, (blue) PCL-0.33% HPPA—5 kDa and (black) PCL-0.33% HPPA-35 kDa.

The Time-of-Flight Secondary Ion Mass Spectrometry (ToF-SIMS) was employed to probe the branched structure of the prepared PCL polymers, and specifically the presence of the HPPA core inside a PCL shell (Figure 6). The ToF-SIMS negative ion spectra collected from a film consisting of neat HPPA macro-initiator show very intense peaks of fragments containing nitrogen, namely CN- (*m/z* = 26.003) and C_3_N- (*m/z* = 50.003), as can be seen in Figure 6 (red curves). On the other hand, these fragments are barely detected for the PCL-0.33% HPPA—35 kDa thin film (see Figure 6, black curves). But the intensity of both the CN- and C_3_N-signals increases (Figure 6, blue curves) if the molecular weight of the probed PCL polymer is much lower (sample PCL-0.33% HPPA—5 kDa). The results in Figure 6 can be explained by keeping in mind that the sampling depth of ToF-SIMS is limited to the outermost layers of the probed sample film (1–1.5 nm from surface for amorphous polymers). Therefore, in the case of the polymer with a molecular mass of approximately 35 kDa, the long PCL chains protect the deeper-located HPPA core. The sevenfold decrease in the molecular mass of the PCL sample (from 35 down to 5 kDa) results in the HPPA core being more easily accessed by ToF-SIMS (due to a thinner shell of PCL chains), and the intensity of the fragments characteristic for HPPA increases (see similar situation in [27]). On the other hand, the simple effect of different ‘dilution’ of HPPA in the probed materials would also be expected to generate a difference in the signals of the CN- and C_3_N-anions. Nevertheless, the huge difference between the sample HPPA (with 18.25 wt.% N according to elemental analysis) and PCL-0.33% HPPA—5 kDa (and 1.26 wt.% N according to elemental analysis) suggests an important contribution of the shielding effect of a wide PCL shell.

#### 2.2.3. Raman Spectroscopy as a Method to Detect the HPPA Core

**Figure 7:**
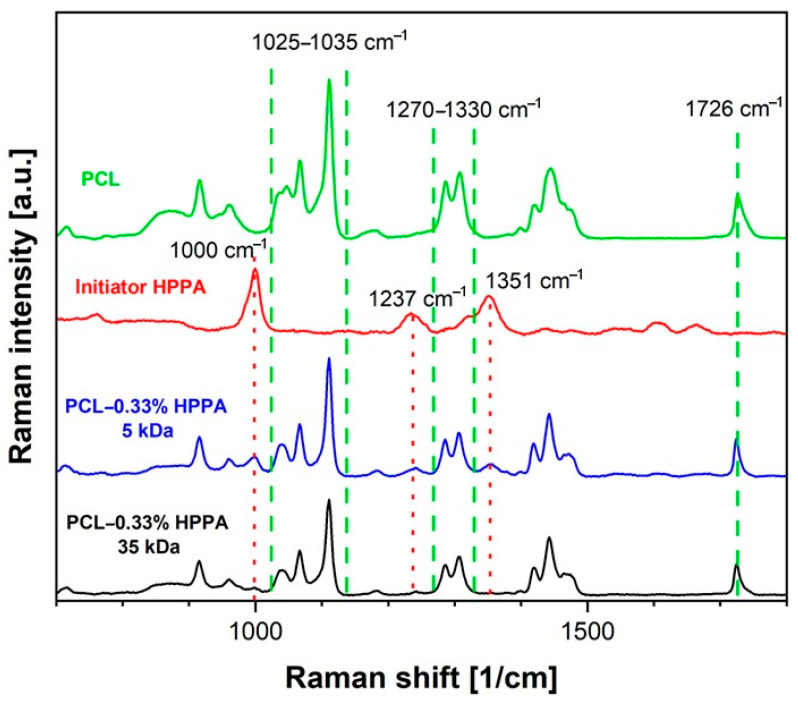
Raman spectra of (green) PCL, (red) HPPA, (blue) PCL-0.33% HPPA—5 kDa and (black) PCL-0.33% HPPA—35 kDa.

Besides ToF-SIMS, the core–shell structure of the prepared highly branched PCL polymers (with HPPA macro-initiator core) was also analyzed by means of Confocal Raman spectroscopy. The Raman spectra recorded for the samples PCL-0.33% HPPA—35 kDa and PCL-0.33% HPPA—5 kDa, as well as for the reference materials pure PCL and pure HPPA, are shown in Figure 7. The spectra for both PCL-HPPA polymers reveal peaks at 1025–1035 cm^–1^ (skeletal stretch), 1270–1330 cm^−1^ (CH_2_ twist) and 1726 cm^−1^ (C=O stretch), which are characteristic for PCL [28]. More interestingly, a few bands are also visible that are characteristic for the macro-initiator HPPA: at 1000 cm^−1^, 1237 cm^−1^ and at 1351 cm^−1^ (corresponding to phenyl group or C-N stretching) [29]. The intensity variations of these signals, if comparing the HPPA, PCL-0.33% HPPA—5 kDa, PCL-0.33% HPPA—35 kDa and PCL samples, prove the presence of HPPA in the highly branched polymers and confirm the trend observed with ToF-SIMS.

### 2.3. Shape and Size of the Highly Branched PCL Macromolecules

The shape and size of the macromolecules of the prepared highly branched PCL polymers were evaluated by observing single macromolecules via Cryogenic Transmission Electron Microscopy (cryo-TEM), and also by means of Dynamic Light Scattering (DLS, size only). The cryo-TEM analyses indicated aggregation of the branched structures in some of the products, which was suspected in view of the GPC chromatograms of samples with higher HPPA contents.

#### 2.3.1. Observation by Cryogenic Transmission Electron Microscopy (Cryo-TEM)

**Figure 8:**
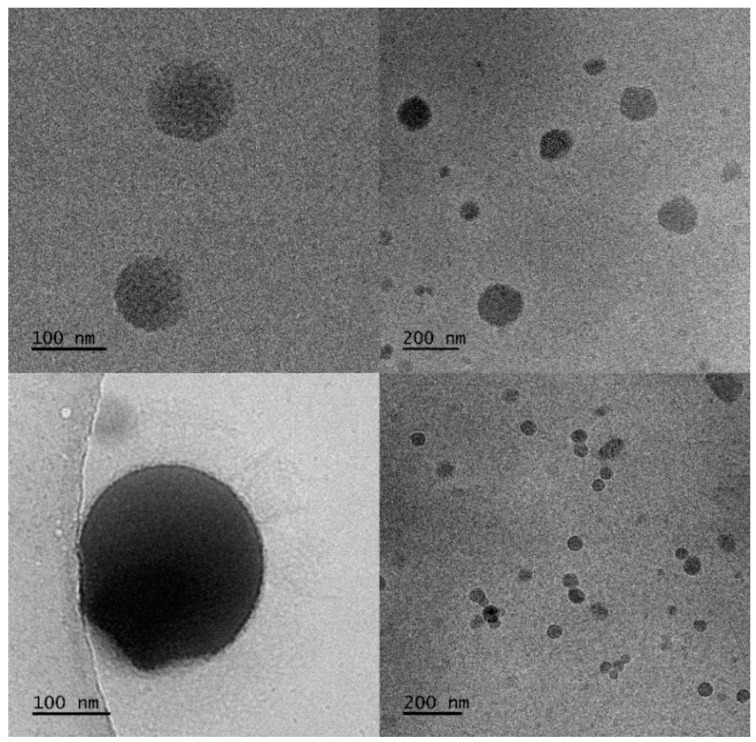
TEM images of PCL-linear.

**Figure 9:**
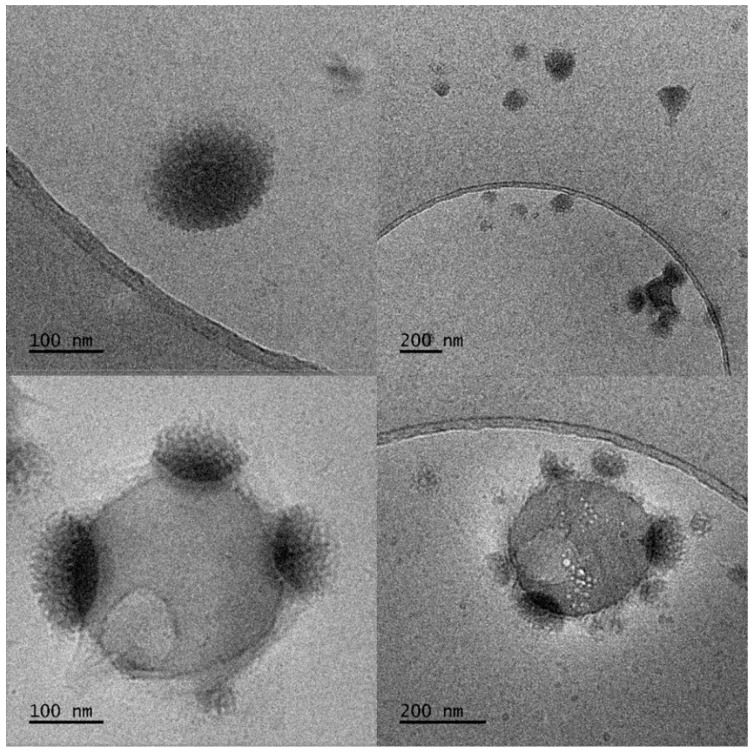
TEM images of PCL-0.13% HPPA.

The cryo-TEM images are compared below for polycaprolactone obtained either by homopolymerization (Figure 8) or by adding 0.13% of HPPA as initiator (Figure 9). There are visible differences between these two polymers: The observed molecules of linear PCL are usually well-separated spherical coils and their structure is homogeneous. In the case of the PCL-0.13% HPPA polymer, it is clear that their shapes are different, as well as the size of the molecules (they are slightly larger). The molecules additionally often form aggregates. Moreover, even in the case of the simplest shapes of the PCL-0.13% HPPA molecular particles, their boundaries are much more diffuse than those of the particles consisting of neat linear PCL.

#### 2.3.2. Size of Macromolecules as Observed by DLS

**Figure 10:**
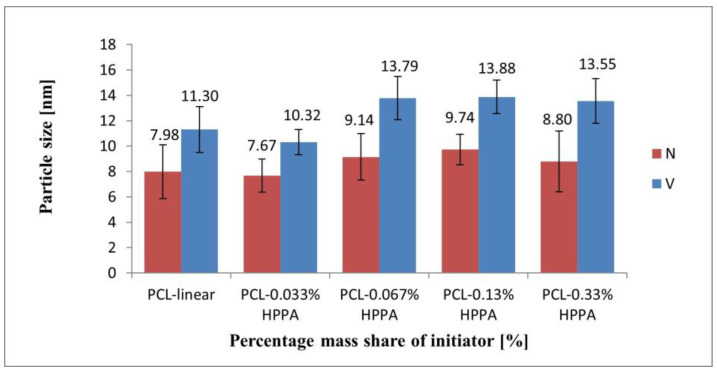
Particle size of linear PCL and PCL with different amount of initiator HPPA (“N” means particle size by number of scattering particles, “V” means particle size by volume of the dispersing phase).

In order to evaluate the average particle size of the different prepared PCL polymers in solution, a dynamic light scattering (DLS) analysis was performed. The obtained results indicate that molecules are about ten times smaller than had been measured by cryo-TEM, which is the effect of preparation of the sample for DLS analysis which included ultrasounding of polymer solution. This step caused breaking of agglomerates, which can be seen in TEM images. On the basis of the DLS analysis, the results of which are summarized in Figure 10, it can be concluded that the linear PCL particles are slightly smaller, while the size of the highly branched PCL particles increases if going from 0.033 to 0.067 wt.% of HPPA added macro-initiator. Between HPPA contents of 0.067 and 0.33 wt.%, the size practically does not change. This latter trend correlates well with the GPC results. Polydispersity indices obtained by DLS measurement are shown in Table 3 for every sample. The polydispersity index indicates how much light is scattered from the various size of particles. For the analyzed samples, PDI is between 0.46–0.49, which indicates medium dispersity of analyzed particles. On the other hand, the absence of a significant difference in DLS between PCL-linear and PCL-0.033% HPPA (4 h reaction), which differed significantly in GPC, indicates a different behavior in solution of the linear (coiled) and of the highly branched polymer.

### 2.4. Phase Behavior of the Products

#### 2.4.1. Thermal Transitions Observed by DSC

**Figure 11:**
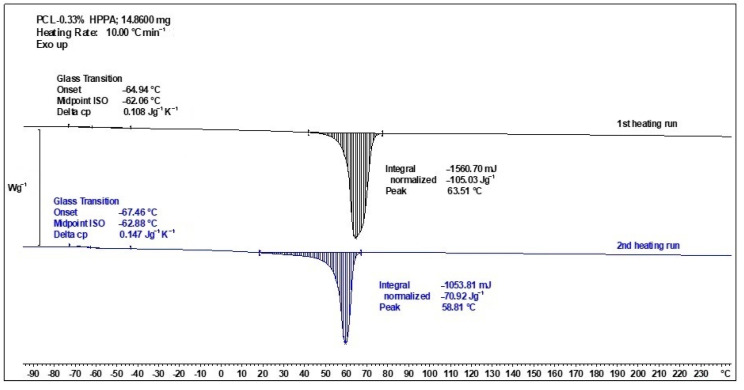
The DSC curve of PCL-0.33% HPPA.

The thermal properties of the polycaprolactone synthesized without (PCL linear) and with the addition of hyperbranched polyamide HPPA were investigated by differential scanning calorimetry DSC (representative samples: see Figure 11; remaining samples Figures S32–S36 in the Supplementary Information File). The DSC results indicate that all polymers have similar thermal properties, obviously due to the dominant fraction of long linear PCL chains. The presented DSC curves show that the polymers melt at about 60 °C, and that the crystalline phase is prominent, while the glass transition of a sufficiently significant amorphous phase can be observed at ca. −60 °C. The melting enthalpy value obtained from the DSC curve allowed the calculation of the degree of crystallinity of each of the tested polymers. The formula (Equation (1)) for calculating the degree of crystallinity, as well as the value of the melting enthalpy of 100% crystalline PCL, were taken from the publication [30]. The data obtained by DSC analysis are summarized further below together with X-ray diffraction results (see Table 4). The crystallinity (as determined by DSC) was found to be 78–79% in the HPPA-initiated samples, while it was somewhat smaller (74%) in the case of PCL-linear. The elongation of polymer chains results in hindering crystallization and reducing the content of the crystalline phase. Linear PCL has longer chains than branched PCL-HPPA polymer, which causes diversity in the degree of crystallinity.

(1) $$Cr=∆H/∆H_{0}\times 100\%$$

where

*Cr*—degree of crystallinity
Δ*H*—enthalpy of melting of the tested PCL
Δ*H*_0_—enthalpy of melting of 100% crystalline PCL, Δ*H*_0_ = 135.5 Jg^−1^ [26].

#### 2.4.2. Crystallinity Observed by XRD

**Figure 12:**
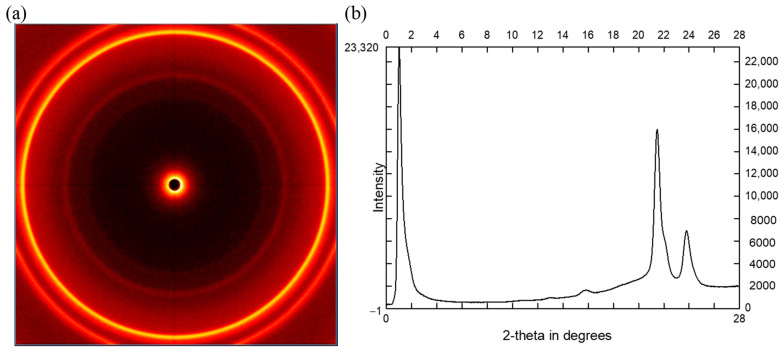
(**a**) PCL-0.33% HPPA XRD image and (**b**) XRD intensity chart.

X-ray diffraction (XRD) analysis (see exemplary XRD image Figure 12 and remaining images Figures S37–S41) confirmed the partial crystallinity of the obtained PCL samples, and it also made possible to determine the degree of crystallinity independently from DSC (discussed above). The diffraction was evaluated using the DIFFRAC.EVA system, which directly yielded calculated crystallinity (amorphous halo was deconvoluted from the sharp reflection peaks of the crystalline phase and the ratio of the crystal peak area to the total area was calculated) [31]. The crystallinity results obtained by XRD are summarized in Table 4. The XRD data suggest that the crystallinity in the highly branched PCL-HPPA products is slightly lower (72–73%) than in PCL-linear (74.5%), except in the case of the polymer with the highest content of HPPA where the crystallinity is slightly higher, at 75.5%. The differences, however, are comparable with the error margin. The higher crystallinity suggested by DSC could be attributed to a fraction of very small or imperfect crystallites, which undergo enthalpy changes connected with melting, but which do not contribute to the sharp crystalline peaks.

#### 2.4.3. Mechanical Properties—Tensile Strength

The tensile strength of some of the obtained polymers was tested and the results are shown in Table 5. 

As mentioned earlier, the degree of crystallinity determined by DSC was generally higher for branched PCL samples if compared to that of linear PCL, but such a trend was not confirmed by the SAXS analysis results. The content of the crystalline phase in polymers affects their mechanical properties, but the size of the crystallites is also important in this case. If there are many small or imperfect crystallites in the sample (as indicated by the results obtained), their impact on the mechanical properties may be different. Moreover, with an increase in the amount of initiator, an enlargement in polymer dispersity is also observed. Small fractions of macromolecules may have a plasticizing effect and, for example, raise the elongation at break and lower the elastic modulus. The complex structure of the obtained branched polymers results in their specific mechanical properties. It can be expected that increasing the content of the macro-initiator during the synthesis will result in a faster growth in branches, which may also be shortened at the same time. The initially obtained results of static tensile strength measurements confirm these expectations to be related to the morphology of the PCL polymers synthesized with the multifunctional HPPA initiator. From the results obtained, the E_mod_ modulus was found to be higher for the branched PCL; it increases with increasing initiator content. Elongation at break is also increased for samples synthesized with the initiator HPPA, if comparing with neat PCL, but the extensibility drops with the increasing HPPA content, and at 0.33% of HPPA, it is lower than for neat PCL-linear. This can be explained by the changing possibility of entanglement of PCL branched chains while the macromolecules become more irregular and polydisperse. These chains can block the elongation of the sample during stretching. The values of the maximum tensile strength F_max_ and the breaking strength F_Br_ for each sample stay at similar levels. However, when F_max_ and F_Br_ values for a particular polymer were compared, more distinct differences were observed for the branched PCL samples than for the linear polymer, suggesting a greater increase in ductility when PCL is grafted on HPPA.

## 3. Materials and Methods

### 3.1. Starting Materials

Tin octanoate Sn(Oct)_2_ 95% was supplied by Sigma-Aldrich (Tokyo, Japan), diethyl ether 99%, tetrahydrofuran (THF) 99% and ε-caprolactone (CL) 98% by Merck (Tokyo, Japan), and all were used as received. The hyperbranched polyamide polymer (oligomer) (HPPA) was synthesized from the 3,5-diaminobenzoic acid (also from Merck) according to a procedure published previously by the authors [22].

### 3.2. Synthesis of PCL

CL was polymerized in the presence of a metalorganic catalyst, tin octanoate Sn(Oct)_2_ and of the hyperbranched polyamide (HPPA) which served as macro-initiator (see Figure 1 in Section 2.1). HPPA is an attractive macro-initiator because of its very mild synthesis conditions (room temperature, several hours stirring) and of its relatively easy isolation. Four different mass ratios HPPA/CL were used in the syntheses (see Table 6). A synthesis without the addition of macro-initiator also was performed, in order to obtain a reference sample (PCL-linear, see Table 6). 

Procedure: ε-caprolactone and HPPA were added into a three-neck round bottom flask, in quantities given in Table 6. Under argon atmosphere, the mixture was heated in an oil bath to 130 °C. Upon reaching this temperature, the catalyst (amount: see Table 6) was added. The reaction mixture was stirred at 130 °C for approximately four hours, which in all cases led to a high monomer conversion—above 90%. The obtained products were purified by dissolution in THF followed by precipitation with diethyl ether. The PCL precipitate was filtered and dried at room temperature for 24 h, and subsequently in 40 °C under reduced pressure (20 mbar).

Observation of kinetics and structure formation: During the synthesis, small samples were taken after 10 min, 60 min, 120 min and 180 min in order to evaluate conversion by means of ^1^H NMR analysis and to obtain kinetic curves of the PCL-HPPA synthesis. Additionally, after 30 min of reaction time (where a molecular mass of about 5 kDa is typically achieved), a reaction sample was taken for performing TOF-SIMS and elemental analysis, as well as Confocal Raman Microspectroscopy characterization.

### 3.3. Characterization

#### 3.3.1. ^1^H Nuclear Magnetic Resonance—^1^H NMR Spectroscopy

^1^H nuclear magnetic resonance spectra were obtained on a Bruker Avance II Plus spectrometer operating at 500 MHz. CDCl_3_ and CD_2_Cl_2_ δ was used as a solvent for PCL-HPPA polymers and DMSO-d6, δ was used as solvent for HPPA initiator, and tetramethylsilane (TMS) as internal reference.

#### 3.3.2. Carbon-13 Nuclear Magnetic Resonance—^13^C NMR

The quantitative Carbon-13 nuclear magnetic resonance spectra were obtained on a Bruker (Ettlingen, Germany) Avance II Plus spectrometer CDCl_3_, δ was used as a solvent for PCL-HPPA polymers and DMSO-d6 and δ was used as solvent for HPPA initiator.

#### 3.3.3. Kinetics Analysis by Means of ^1^H NMR

^1^H NMR analysis was also employed to determine the degree of conversion of the ε-caprolactone monomer and to follow the kinetics of the reaction. In order to calculate the conversion degree, integration of characteristic signals for the polymer and the monomer was performed. Using formula (Equation (2)) the degree of conversion was obtained:

(2) p=P/(M+P)·100%

where

*P*—the integration value of the signal coming from the polymer
*M*—the integration value of the signal coming from the monomer
*p*—degree of conversion

#### 3.3.4. FT-IR Spectroscopy

FT-IR spectroscopy was performed to confirm the presence of characteristic functional groups in the obtained PCL. The spectra were recorded using a Nicolet 6700 FT-IR Spectrometer from Thermo Fisher (Waltham, MA, USA), in the range of 4000 to 400 cm^–1^. Samples were prepared as KBr pellets in the case of PCL and PCL-HPPA polymer and by ATR method for caprolactone monomer.

#### 3.3.5. Gel Permeation Chromatography—GPC

Gel permeation chromatography was performed using the columns PSS SDV Guard and PSS SDV 100, 1000 and 10,000 Å, grain diameter 5 µm, in combination with the Shodex RI-71 differential refractometer. The data were evaluated using OmniSEC software, version 4.2. The samples were dissolved (5 mg/mL) in tetrahydrofuran (THF). The HPPA initiator sample was dissolved in dimethylformamide (DMF) (5 mg/mL). The analyses were carried out at room temperature. The solvent flow rate was 1 mL/min, and the loop volume was 100 μL. Calibration of the chromatographic system was carried out using polystyrene standards.

#### 3.3.6. Dynamic Light Scattering—DLS

The size of the branched polymer particles was determined by dynamic light scattering (DLS). This characterization was performed with a DLS Zetasizer Nano analyzer (Malvern, Worcestershire, UK) equipped with a laser with a wavelength of 644 nm. Analysis was performed at scattering angles 173° at 20 °C, using cumulants analysis method. The samples were prepared as 1 mg/mL solutions of the obtained polymers in dimethylfumarate. Then, the solution was ultrasounded and filtered through a Teflon syringe filter with a pore diameter of 0.2 µm. The analysis was carried out in glass cuvettes supplied with the DLS analyzer used.

#### 3.3.7. Differential Scanning Calorimetry—DSC

The DSC analyses were performed on the differential scanning calorimeter DSC from Mettler Toledo, using STAR^e^ System software (version 16.2). The calorimeter was calibrated with indium and zinc supplied by Mettler Toledo. The curves were recorded at the heating rate of 10 K/min, under nitrogen flow of 60 mg/mL. The samples with a mass of 7–10 mg were loaded into aluminum DSC pans with a volume of 40 μL, and then sealed with a lid with a small hole. All thermograms were recorded with an empty aluminum pan as reference.

#### 3.3.8. X-ray Diffraction—XRD

X-ray diffraction analyses (XRD) were performed on a NanoStar-U diffractometer from Bruker. X-rays of the wavelength λ = 1.54 Å were produced by a copper lamp, powered by 600 µA at 50 kV. The diffractometer was equipped with two Göbel mirrors for monochromating and aligning a beam of 500 µm diameter. The range of scattering angles was 0° to 28°. The analyses were carried out at room temperature (22 ± 2 °C), with powdered samples filled into glass capillaries.

#### 3.3.9. Cryogenic Transmission Electron Microscopy—Cryo-TEM

Cryogenic Transmission Electron Microscopy (cryo-TEM) images were obtained using a Tecnai F20 X TWIN microscope (FEI Company, Hillsboro, OR, USA) equipped with field emission gun, operating at an acceleration voltage of 200 kV. Images were recorded using the Gatan Rio 16 CMOS 4k camera (Gatan Inc., Pleasanton, CA, USA) and processed with Gatan Microscopy Suite (GMS) software version 3.31.2360.0 (Gatan Inc., Pleasanton, CA, USA). Specimen preparation was carried out by vitrification of the aqueous solutions on grids with holey carbon film (Quantifoil R 2/2; Quantifoil Micro Tools GmbH, Großlöbichau, Germany). Prior to use, the grids were activated for 15 s in oxygen plasma using a Femto plasma cleaner (Diener Electronic, Ebhausen, Germany). Cryo-samples were prepared by applying a droplet (3 μL) of the suspension to the grid, blotting with filter paper and immediate freezing in liquid ethane using a fully automated blotting device Vitrobot Mark IV (Thermo Fisher Scientific, Waltham, MA, USA). After preparation, the vitrified specimens were kept under liquid nitrogen until they were inserted into a cryo-TEM-holder Gatan 626 (Gatan Inc., Pleasanton, USA) and analyzed in the TEM mode at −178 °C.

#### 3.3.10. Time-of-Flight Secondary Ion Mass Spectrometry—ToF-SIMS

To examine the products with ToF-SIMS, the samples PCL-0.33% HPPA (molecular mass: 5 kDa) and PCL-0.33% HPPA (molecular mass: 35 kDa) were dissolved in tetrahydrofuran, while HPPA was dissolved in dimethylformamide, all with concertation of 20 mg/mL. Then, the solutions were spin casted (ω = 3000) on silicon substrates. The polymeric surfaces were analyzed with a TOF.SIMS 5 instrument (IONTOF GmbH, Münster, Germany) using a 30 keV bismuth liquid metal ion gun and Bi^3+^ clusters. The negative ions spectra were recorded from at least four different non-overlapping 200 µm × 200 µm regions, with a high mass resolution of m/Δm > 6100 at C_3_- (*m*/*z* = 36). To ensure a static mode condition, the dose density deposited on the surface was less than 10^12^ ions cm^–2^ for all the measured points.

#### 3.3.11. Raman Spectroscopy

Raman spectroscopy analyses were conducted with powdered samples using a Confocal Raman Microscope System Alpha 300R (WITec, Ulm, Germany), with a UHTS300 spectrometer and with a DR316B_LD CCD detector employing a 300 g mm^–1^ grating. A 785 nm laser set with a laser power of 90 mW in front of the objective was used. Spectral data were acquired using a 20× objective ((NA 0.4) EC Epiplan, Zeiss, Gina, Germany). Single spectra were recorded in a range of 100–3560 cm^−1^, with an integration time of 4 s and with 10 accumulations. Each sample was measured in five different spots.

All acquired Raman spectra were further processed using the WITec ProjectSIX 6.1 software. All spectra were treated with a cosmic-ray-removal and a background subtraction procedure, and the “shape” function was applied, when necessary. After that, all spectra for each sample were averaged using the “average spectra” function.

#### 3.3.12. Elemental Analysis

Elemental analysis for C, H and N was carried out with a Carlo-Erba EA 1108 analyzer from Thermo Fisher Scientific Inc., Waltham, MA, USA.

#### 3.3.13. Mechanical Properties—Tensile Strength

Samples for tensile strength testing were prepared by injection molding using a Haake ThermoScientific micro-injection molding machine with parameters: cylinder temperature –75 °C, plasticization time 120–150 s, mold temperature 28 °C, injection time 16 s, injection pressure 400 bar, clamping time 20 s, clamping pressure 300 bar.

The obtained samples were tested on the INSTRON 5967 universal testing machine (Instron, Grove City, PA, USA). The tests were carried out in conformity to the ISO 527-1:2020 standard [32]. The used tensile speed was 5 mm/min.

## 4. Conclusions

A simple and efficient solvent-free method for the synthesis of highly branched polycaprolactone (PCL) has been developed, with a highly branched polyfunctional core and with a shell consisting of linear PCL chains. The aromatic-amino-functionalized polyamide macro-initiator (10.5-mer) named HPPA was used at synthesis contents between 0.033 and 0.33 wt.%. HPPA is an attractive macro-initiator because of its very mild synthesis conditions (room temperature, several hours stirring) and its relatively easy isolation.

NMR and elemental analysis confirmed the presence and the covalent incorporation of HPPA in the products. A high grafting efficiency (81%) of the amino groups of HPPA was also proven.

The polymers initiated by HPPA were found to always possess higher molecular masses (GPC) than self-polymerized PCL.

Polymerization kinetics indicated that increasing amounts of HPPA macro-initiator distinctly accelerate the conversion of the ε-caprolactone monomer, and hence that PCL is preferably grafted onto HPPA rather than self-polymerizing. The dispersity of the HPPA-PCL copolymers significantly rises with higher contents of HPPA, most likely due to occasional transesterification reactions which can connect growing branched PCL macromolecules.

Confocal Raman Microspectroscopy, and especially Time-of-Flight Secondary Ion Mass Spectrometry (ToF-SIMS), supported the existence of a core–shell structure in the products.

Direct observation of macromolecules by means of cryogenic transmission electron microscopy (cryo-TEM), and also gel permeation chromatography (GPC), indicated the existence of an ‘aggregated’ product fraction with multiple HPPA cores in the case of higher HPPA initiator contents in the reaction mixture.

Some differences between GPC and dynamic light scattering (DLS) results confirmed a specific solution behavior of the highly branched PCL–HPPA products.

Differential scanning calorimetry, as well as X-ray diffraction, demonstrated that the PCL-HPPA polymers displayed a similar degree of crystallinity as linear neat PCL, but that the branched products possessed smaller and less regular crystallites.

Mechanical properties analyzed in tensile tests confirm expectations related to the morphology of the PCL-HPPA polymers; the E_mod_ modulus and elongation at break were found to be higher for the branched PCL compared with neat PCL.

## Figures and Tables

**Table 1:** Elemental analysis summary.

Sample	C [%]	H [%]	N [%]
PCL-linear	59.83	8.230	0
PCL-0.33% HPPA—35 kDa	61.76	8.351	0.26
PCL-0.33% HPPA—5 kDa	64.43	8.48	1.26
HPPA-initiator	55.33	5.247	18.25

**Table 2:** Molecular data summary of PCL.

Sample	*M_n_* [Dalton]	*M_w_* [Dalton]	*M_z_* [Dalton]	*M_p_* [Dalton]	*M_w_*/*M_n_*
PCL-linear	28,850	45,850	86,470	33,120	1.59
PCL-0.033% HPPA	37,110	67,030	126,700	45,370	1.80
PCL-0.067% HPPA	34,200	67,060	157,050	41,210	1.96
PCL-0.13% HPPA	36,630	88,440	270,400	42,430	2.41
PCL-0.33% HPPA	35,270	98,320	286,780	39,550	2.78

**Table 3:** Polydispersity index obtained by DLS measurement.

Sample	Polydispersity Index PDI
PCL-linear	0.49
PCL-0.033% HPPA	0.46
PCL-0.067% HPPA	0.49
PCL-0.13% HPPA	0.46
PCL-0.33% HPPA	0.49

**Table 4:** DSC parameters and degree of crystallinity calculated from DSC and SAXS analysis.

Polymer	Tg [°C]	Tm [°C]	Cr % DSC ^1^	Cr % SAXS
PCL linear	−59.8	63.8	73.9	74.5
PCL-0.033% HPPA	−61.3	60.8	79.4	73.0
PCL-0.067% HPPA	−62.1	60.1	77.6	72.8
PCL-0.13% HPPA	−65.6	64.0	78.4	72.0
PCL-0.33% HPPA	−62.06	63.5	77.5	75.5

^1^ determined for the first heating cycle during the DSC analysis.

**Table 5:** Tensile strength results (E_mod_—Young’s modulus, F_max_—maximum tensile strength, F_Br_—breaking strength).

Sample	E_mod_	F_max_	Elongation at Break	F_Br_
	MPa	MPa	%	MPa
PCL-linear	331.0	21.9	338.8	21.2
PCL-0.033% HPPA	341.2	19.4	424.5	15.4
PCL-0.067% HPPA	425.2	21.4	371.5	15.3
PCL-0.13% HPPA	446.2	25.2	360.6	22.1
PCL-0.33% HPPA	485.7	21.9	317.5	15.0

**Table 6:** Conditions of PCL-HPPA syntheses.

Sample	Time [Hours]	Initiator—HPPA Percentage Mass Share [%]	Monomer ε-Caprolactone CL [mL]	Catalyst Sn(Oct)_2_ [mL]
PCL-linear	4	0	21	0.03
PCL-0.033% HPPA		0.033		
PCL-0.067% HPPA		0.067		
PCL-0.13% HPPA		0.13		
PCL-0.33% HPPA		0.33		

## Data Availability

Data is contained within the article and Supplementary Material.

## Supplementary Materials

The following supporting information can be downloaded at: https://www.mdpi.com/article/10.3390/molecules29050991/s1.
