# Supplementary material for: Synthesis and Morphology Characteristics of New Highly Branched Polycaprolactone PCL
Source: Molecules. 2024 Feb 24;29(5):991. doi: 10.3390/molecules29050991 (PMC10934558; doi:10.3390/molecules29050991)
Supplement: Supplementary file 1 [file molecules-29-00991-s001.zip › molecules-2798150-supplementary.pdf]

SUPPLEMENTARY INFORMATION FILE

Synthesis and Morphology Characteristics of New Highly Branched Polycaprolactone PCL

Aleksandra Zioło [1,2,*], Beata Mossety-Leszczak [2], Małgorzata Walczak [2], Beata Strachota [3], Adam Strachota [3], Kamil Awsiuk [4], Natalia Janiszewska [4,5] and Joanna Raczkowska [4]

[1] Doctoral School of the Rzeszów University of Technology, al. Powstańców Warszawy 12, 35-959 Rzeszow, Poland
[2] Department of Industrial and Materials Chemistry, Faculty of Chemistry, Rzeszow University of Technology, al. Powstańców Warszawy 12, 35-959 Rzeszow, Poland; mossety@prz.edu.pl (B.M.-L.); mwalczak@prz.edu.pl (M.W.)
[3] Institute of Macromolecular Chemistry, Czech Academy of Sciences, Heyrovského nám. 2, 162 00 Praha, Czech Republic; beata@imc.cas.cz (B.S.)
[4] M. Smoluchowski Institute of Physics, Faculty of Physics, Astronomy and Applied Computer Science, Jagiellonian University, Łojasiewicza 11, 30-348 Krakow, Poland; natalia.janiszewska@doctoral.uj.edu.pl (N.J.)
[5] Doctoral School of Exact and Natural Sciences, Jagiellonian University, Łojasiewicza 11, 30-348 Krakow, Poland
* Correspondence: d527@stud.prz.edu.pl

1. Possible structures of the initiator molecule.

The HPPA macro-initiator employed in this work was subjected to a thorough analysis, in order to assess its primary structure, i.e. the molecular mass, the number of reactive amino functions (which initiate caprolactone polymerization), as well as the degree of branching.

The Figures below illustrate the structures of dendrimeric (maximum branching), linear (no branching) and partly branched HPPA polymers.

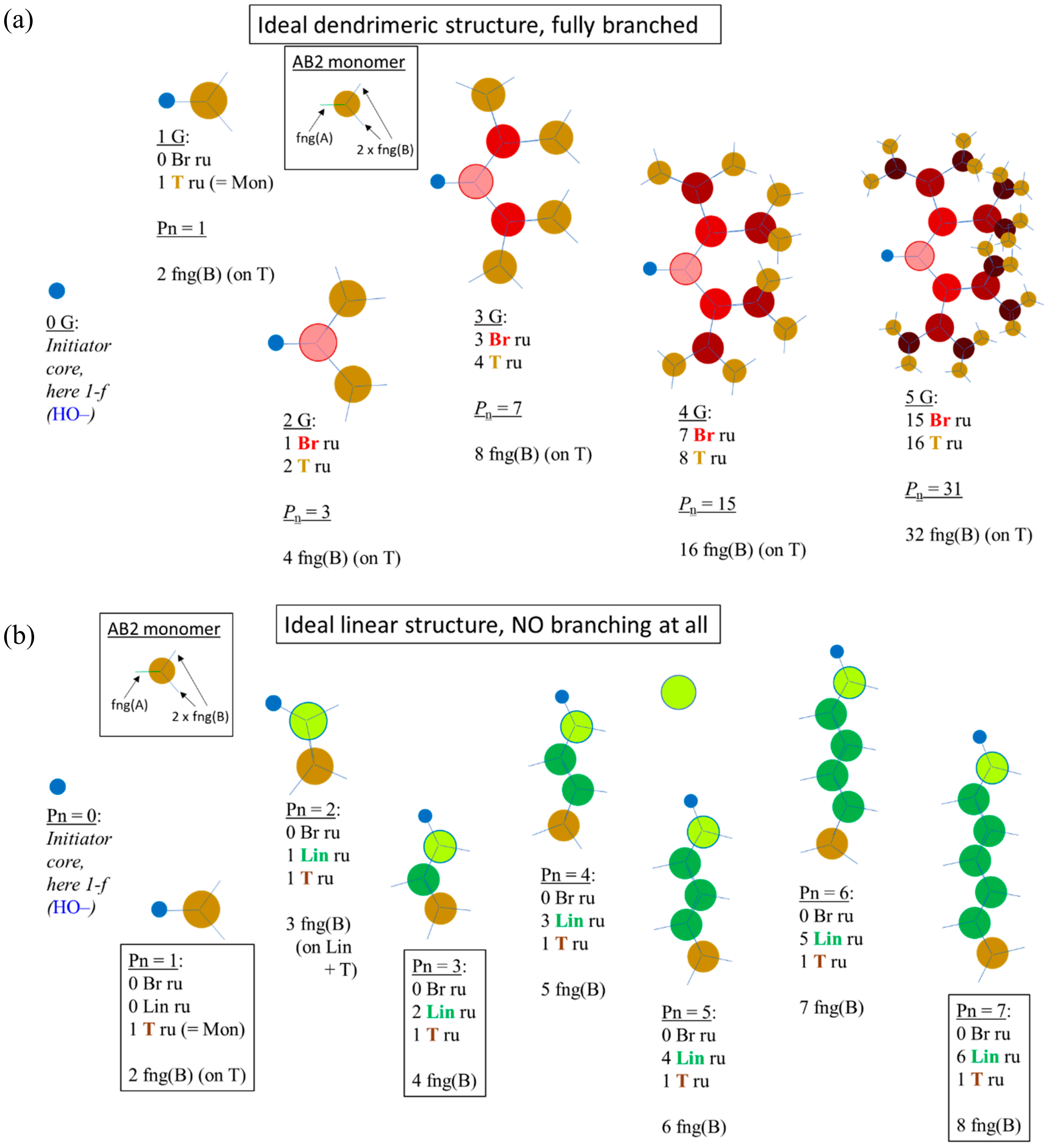

Figure S1. *consideration of the molecular structure of the HPPA initiator molecule and of the numbers of different types of repeat units and of the residual functional groups:*
(a) ideal dendrimeric structure; (b) ideal linear structure.

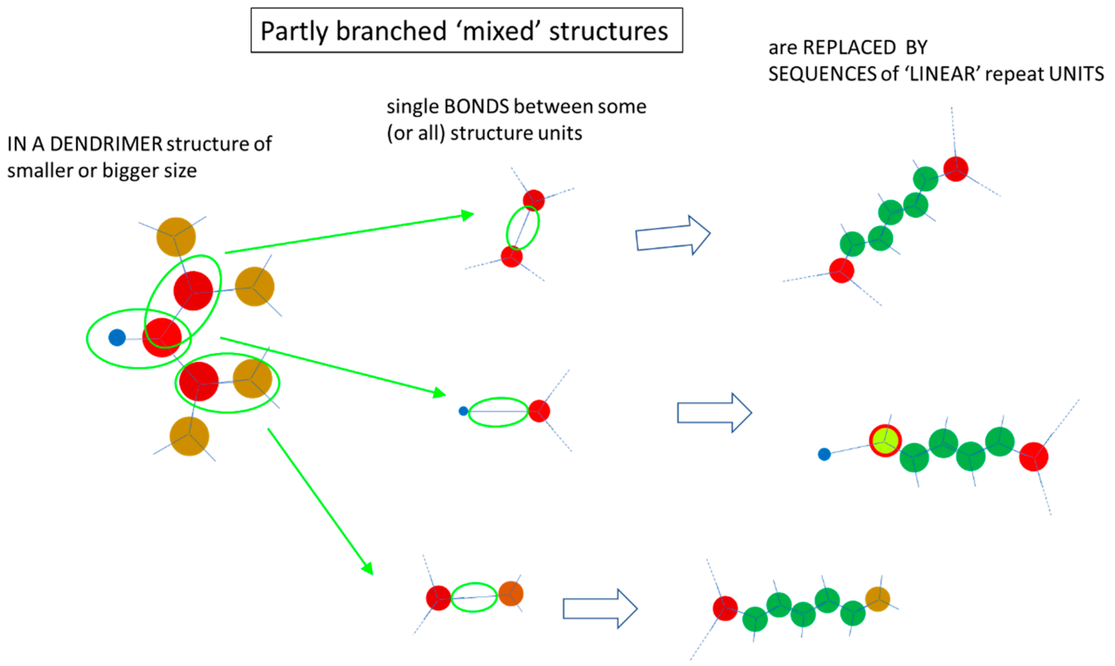

Figure S2. *consideration of the molecular structure of the HPPA initiator molecule and of the numbers of different types of repeat units and of the residual functional groups: partly branched structure.*

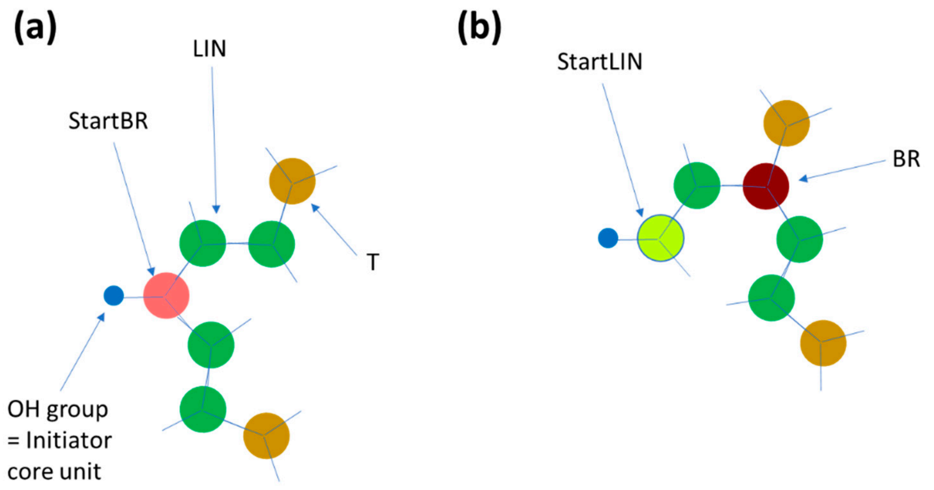

Figure S3. *types of repeat units in partly branched molecules: (a)* ***StartBR*** *= branching type of 'starting' unit bonded to core, in our case the repeat unit which possesses an unreacted COOH group, but whose both amino groups are reacted;* ***LIN*** *= linearly bonded repeat unit;* ***T*** *= terminal unit; (b)* ***StartLIN*** *= linearly bonded starting unit in which also one of the amino group is not reacted;* ***BR*** *= 'normal' branching unit in the polymer, which is not a starting one.*

Mathematic relations: numbers of repeat units and functional groups

For the ideal Dendrimeric structure, it holds:

number of **surface** repeat **units** (= Terminal units, "T"):

$$(n_T) = N_c \, N_b^{G-1} \qquad \text{(S1)}$$

where:
G is the number of generations (G=0 for core without monomer, G = 1 for core bonded to one or several Monomer units, depending on Nc)
N_b is the branch-juncture multiplicity
N_c is the initiator-core multiplicity (simple dendrimer: $N_c = 1$)

number of **branching units** ("BR") (internal dendritic units):

$$(n_{BR}) = N_c \, (1 - N_b^{G-1}) / (1 - N_b) \qquad \text{(S2)}$$

the equation exploits the formula for the sum (s_n) of geometric series:

$$s_n = a\,r^0 + a\,r^1 + a\,r^2 + \ldots + a\,r^{(n-1)}$$

where a and r are two constants:

$$s_n = a\,[\,(1 - r^n) / (1 - r)\,] \quad , \text{ for } r \neq 1$$

->>in some literature, $(N_b^{G-1} - 1) / (N_b - 1)$ is used instead of $(1 - N_b^{G-1}) / (1 - N_b)$;

the current form was preferred due to more clear exponent syntax (in "N_b^{G-1}")

degree of polymerization, ("Pn") (= number of all repeat units):

$$(P_n) = N_c \, (1 - N_b^{G}) / (1 - N_b) \qquad \text{(S3)}$$

surface(T) / dendritic r.u. (BR) ratio:

$$(T) / (BR) = N_c N_b^{G-1} / [\, N_c (1 - N_b^{G-1}) / (1 - N_b)\,] = (1 - N_b)\,(N_c N_b^{G-1}) / [\, N_c (1 - N_b^{G-1})] \qquad \text{(S4)}$$

molecular mass (MW):

$$(MW) = M_{core} + N_c \, (n_{BR} M_{BR} + n_T M_T) \qquad \text{(S5)}$$

where:

M_{core} is the molecular mass of the core to which the first monomer generation is attached (here the core is an OH group)

M_{BR} is the molecular mass of the Branching repeat units

M_T is the molecular mass of the Terminal (surface-) repeat units

Common features of Dendrimeric, Linear and Partly Branched products:

number of functional groups (B) (B2 from AB2 monomer): “fng”:

$$fng = P_n + 1 \qquad \text{(S6)}$$

this holds for the Branched as well as Linear polymers made from AB2-monomers, and hence also for the Partly Branched one

in the studied case *fng* means the number of the amino groups

number of amide groups: “n(C-NH-CO-)”

$$n(\text{C-NH-CO-}) = P_n - 1 \qquad \text{(S7)}$$

this holds for the Branched as well as Linear structures, and hence also for the Partly Branched one

number of Terminal repeat units vs Branching (dendritic) units:

$$n(\text{Terminal units}) = n(\text{Branching units}) + 1 \qquad \text{(S8)}$$

all repeat units in the polymer possess precisely 1 carbonyl group, which usually is an amide group, except for the first ‘starting’ repeat unit which is bonded to the nominal “OH-core” and hence is a COOH group (different also in 13C-NMR)

there is only 1 COOH group (unreacted “A” group) **in any** AB2-polymer **macromolecule**

simplified formula for molecular mass, for the studied AB2-type polymer:

$$\text{MW} = M(\text{HO}) + P_n\, M(\text{RU}) + (P_n+1)\, M(\text{H}) \qquad \text{(S9)}$$

where M(RU) is the molecular mass of the branching repeat unit, HO = hydroxyl, H = hydrogen

2. Analysis of molecular mass distribution of the Initiator macromolecule

The molecular masses of the HPPA macro-initiator and their distribution were analyzed by means of GPC in this work. Additionally, mathematical analysis was performed on the MALDI-TOF mass spectrum of the same HPPA product, which was recorded in a previous work [22].

GPC HPPA initiator molecular mass distribution curve

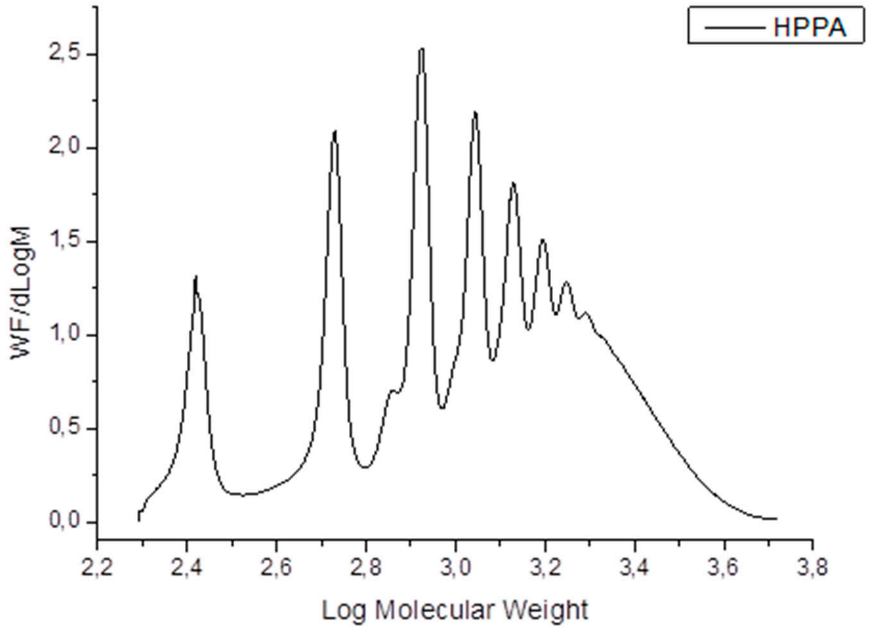

Figure S4. *HPPA initiator molecular mass distribution curve, prepared in DMF as a solution.*

The **GPC chromatogram** (Figure S4) shows a **similar trend**, albeit somewhat distorted, **like the MALDI-TOF spectrum** discussed in the next sub-section. In the latter case, the precise molecular masses were measured, and also their mathematical evaluation was simpler than in case of the GPC chromatogram, which displays to a series of more or less separated molecular peaks, followed by a continuous region.

Hence, the analysis of the MALDI-TOF data was used for obtaining the most accurate values of the molecular masses M_n, M_w, and of the dispersity *Đ*, as well as of the number average polymerization degree and of the number average amino-functionality (*fng*).

Mathematic evaluation of the MALDI-TOF analysis from previous work

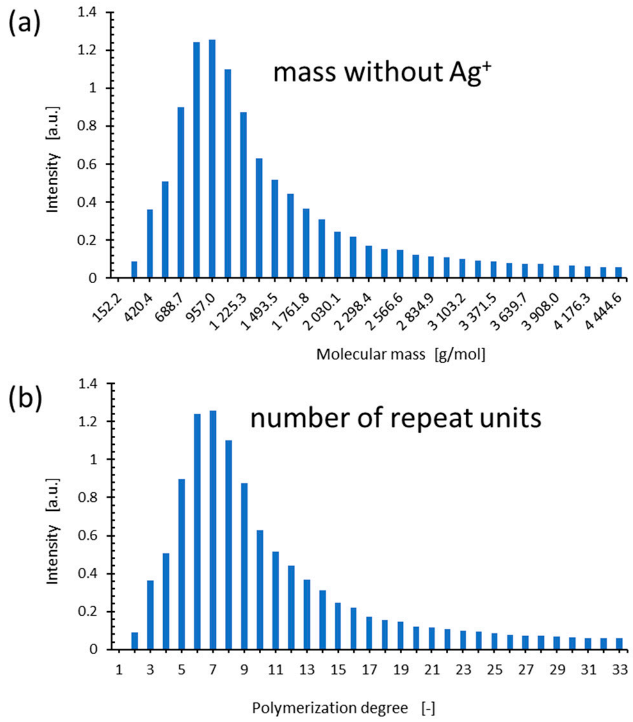

Figure S5. *simplified representation of the distribution of molecular masses as determined by MALDI-TOF, as determined in the authors' previous work [22]: (a) true molecular masses without the adsorbed Ag cations; (b) distribution of polymerization degrees; The data can be found in Table S1.*

using *Formula (S9)* presented further above, it can be written for the given polymer:

MW = 17.007 g/mol + P_n * 133.13 g/mol + (P_n+1) * 1.008 g/mol

The data in *Table S1* can be used for calculating the number average (M_n) and the mass average (M_w) molecular masses as well as the dispersity ($Đ$):

M_n = 1428.216 g/mol (**P_n** = ca. 10.5, with 11.5 NH2 groups)

M_w = 1958.798 g/mol (**P_n** = ca. 14.5, with 15.5 NH2 groups)

$Đ$ = 1.37

the most frequent molecule: **P_n** = ca.6.5, M = 889.912 g/mol, with 7.5 NH2 groups.

Table S1. *Molecular masses and their relative prominence, as determined by MALDI-TOF in previous work [22].*

***MW* (g/mol)**	**P_n (-)**	***Intensity* (a.u.)**
152.153	1	0
286.291	2	0.09
420.429	3	0.36147
554.567	4	0.50688
688.705	5	0.89759
822.843	6	1.24026
956.981	7	1.25627
1091.119	8	1.10117
1225.257	9	0.87526
1359.395	10	0.62953
1493.533	11	0.51658
1627.671	12	0.4424
1761.809	13	0.3678
1895.947	14	0.30963
2030.085	15	0.24514
2164.223	16	0.21943
2298.361	17	0.17096
2432.499	18	0.15495
2566.637	19	0.1482
2700.775	20	0.12249
2834.913	21	0.11617
2969.051	22	0.10943
3103.189	23	0.09973
3237.327	24	0.09341
3371.465	25	0.08709
3505.603	26	0.0774
3639.741	27	0.07402
3773.879	28	0.07402
3908.017	29	0.0677
4042.155	30	0.06433
4176.293	31	0.06096
4310.431	32	0.05801
4444.569	33	0.05801

3. Initiator macromolecule: its molecular structure and degree of branching.

The primary structure of the highly branched HPPA macro-initiator was analyzed by means of ^{13}C-NMR (Figures S6–S11) and ^{1}H-NMR (Figures S12 and S13) spectroscopy. Of especial interest was the degree of branching (relative prominence of branching units) in the HPPA product.

<u>^{13}C-NMR analyses summary</u>:

The spectrum can be divided into several regions (detailed analyses and deconvolutions are further below). Some of the peak groups made possible to differentiate all or some of the various types of repeat units in HPPA, and eventually to calculate $\boldsymbol{P_n}$:

<u>Carbonyl signals of</u> CO(-OH) and CO(-NH-) groups yielded
$\boldsymbol{P_n}$ = ca. 6.7 (not very accurate);
StartLIN / StartBR = ca. 2.55 / 1 (this concerns only the Start units);
(LIN+T) / BR = ca. 2.23 / 1 (not very accurate but highlights the **prominence of LIN units**);

<u>C(-NH$_2$) signals</u> : LIN / T = 2.60 / 1; (LIN + Start_LIN) / T = 2.92 / 1
=> **prominence of LIN units**

<u>C(-NH-CO-) signals</u> : no good signal separation; confirmed: *number*(C-NH2) ≈ *number*(C-NH-CO-) => very **small oligomers are not prominent**

<u>C(-COOH), C(-CO-NH-)</u> signals: useful structure information:
ratio **BR / LIN / T = ca. 0.65 / 3 / 1** , Start units not resolved (overlap with other signals)
The ratio is in good agreement with a **10-mer containing 1 Start_LIN unit, 1 BR unit (above ratio: 1.3), 6 LIN units, and 2 T units.** ($\boldsymbol{P_n}$ not accessible from the signals).

<u>C-H</u>: LIN/BR = ca. 2.15; (LIN + StartLIN)/BR = ca. 2.55 => **prominence of LIN units**
$\boldsymbol{P_n}$ **= ca. 7.75** (not highly accurate)

<u>^{1}H-NMR analyses summary</u>:

more difficult evaluation (couplings and overlaps); detailed analyses are further below.
$\boldsymbol{P_n}$ **= ca. 8.8** ; 1 BR unit in the molecule

<u>Final conclusions:</u>

The more accurate was the determination of $\boldsymbol{P_n}$, the closer it came to the value determined by MALDI-TOF (**10.5 repeat units**). This value hence was accepted for the numeric average molecule. C(-NH$_2$) signals and the C(-COOH), C(-CO-NH-) peak group bots indicate, that the ratio LIN/T is between 3 and 2.6, while C(-COOH)&C(-CO-NH-) and also the ^{1}H-NMR spectrum (aromatic region) suggest, that the number of branching units in a HPPA molecule is close to 1 (1.3). **Hence, the mix of molecules closest to average would consist of 4 molecules, two 10-mers and two 11-mers, three of which contain one BR unit, while one 11-mer contains 2 BR units. 3 out of 4 Start (with COOH) units are linearly bonded.**

Structures of the numeric average and of the most frequent HPPA molecules are shown below, in Scheme S1 and Scheme S2.

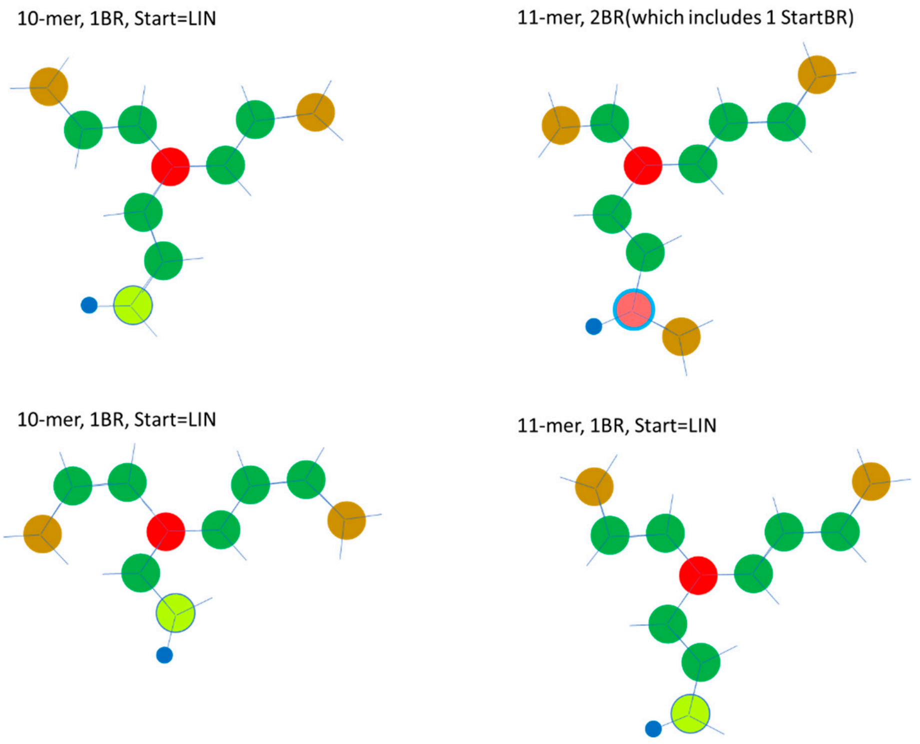

Scheme S1. *The structures closest to the* *<u>numeric average HPPA molecule</u>*: *10- and 11-mers (as determined by MALDI-TOF), among which 1 in 4 has two branching units (instead of typical one), and 1 in 4 has a branching Start unit. The ratios of the various repeat units are closest possible to the experimentally determined ones. The types of repeat units are highlighted by color.*

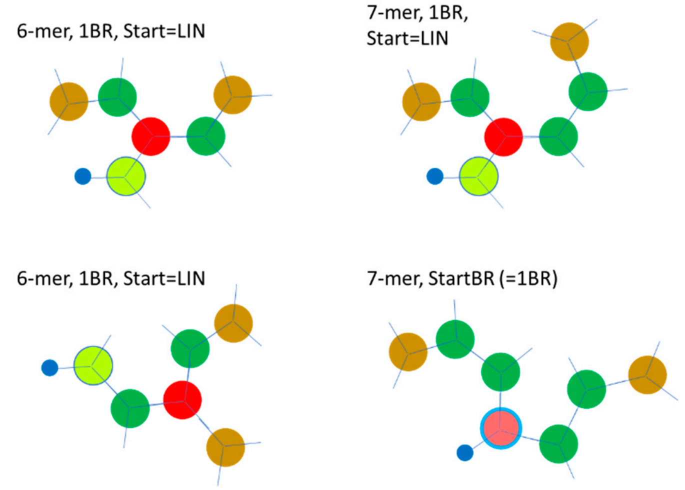

Scheme S2. *The structures closest to the* *<u>most frequent HPPA molecule</u>* *size (as determined by MALDI-TOF): 6- and 7-mers containing usually one branching unit (ca. 1 in 6 should have two) and 1 in 4 has a branching Start unit. The types of repeat units are highlighted by color.*

The detailed evaluation of the spectra follows below.

^{13}C-NMR spectroscopy

The *Figure S6* shows the quantitative ^{13}C-NMR spectrum of the HPPA macro-initiator (recorded in DMSO-d6 solution), and the assignment of its resonance signals. The relatively simple spectrum made possible the evaluation of the relative prominence of Branching, Linear, 'Starting', and Terminal units in the HPPA macromolecule.

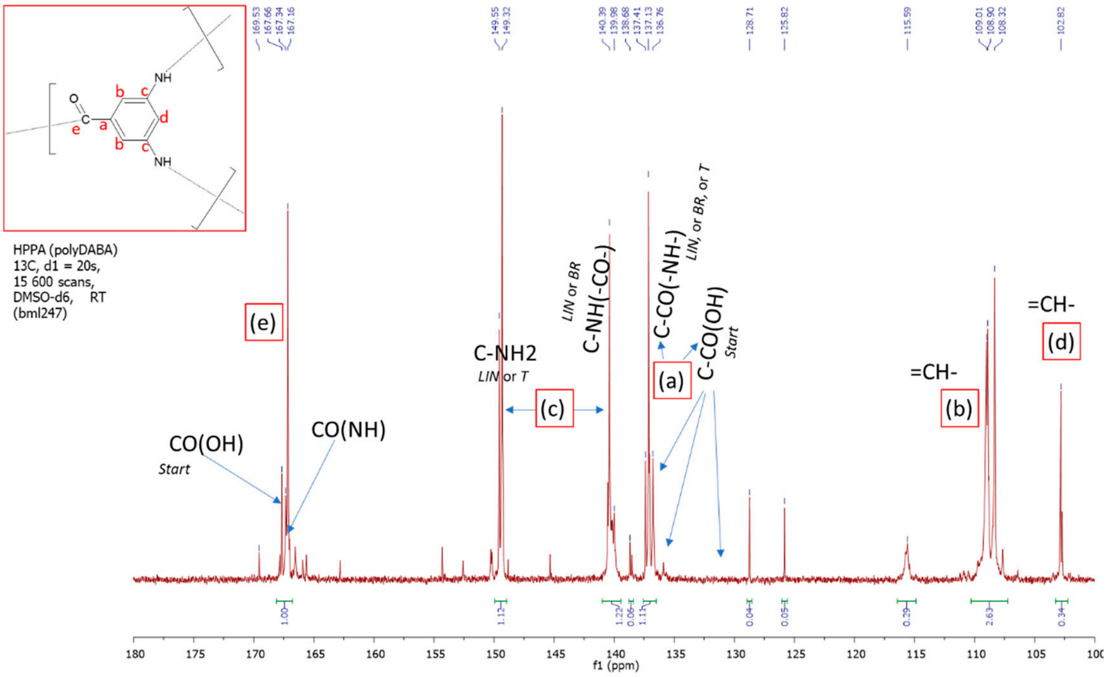

Figure S6. *^{13}C-NMR spectrum of the HPPA macro-initiator, recorded in DMSO-d6 solution (solvent peak is outside of the depicted shift range), and the assignment of its resonance signals; abbreviation "LIN" means "linear repeat units", "BR" means "branching (dendritic) repeat units", "T" means "terminal (outermost) repeat units", "Start" means "starting repeat unit" (the first unit of the macromolecule with COOH group still intact).*

For analyzing the relative prominence of the different repeat units zoomed regions of their signals are depicted on the next pages, along with deconvolution analysis (fit with sum of Lorentz functions).

The following form of the Lorentz function was used:

$$Y = (height * width^2)/(width^2 + 4 * (position - X)^2)$$

where the *height* and *width* parameters are constants defining peak height and width, and the *position* constant is the peak position on the *X* axis. *X* and *Y* axes in the given case are the chemical shift in ppm and the peak intensity.

The detailed analysis of the ^{13}C-NMR spectra follows below.

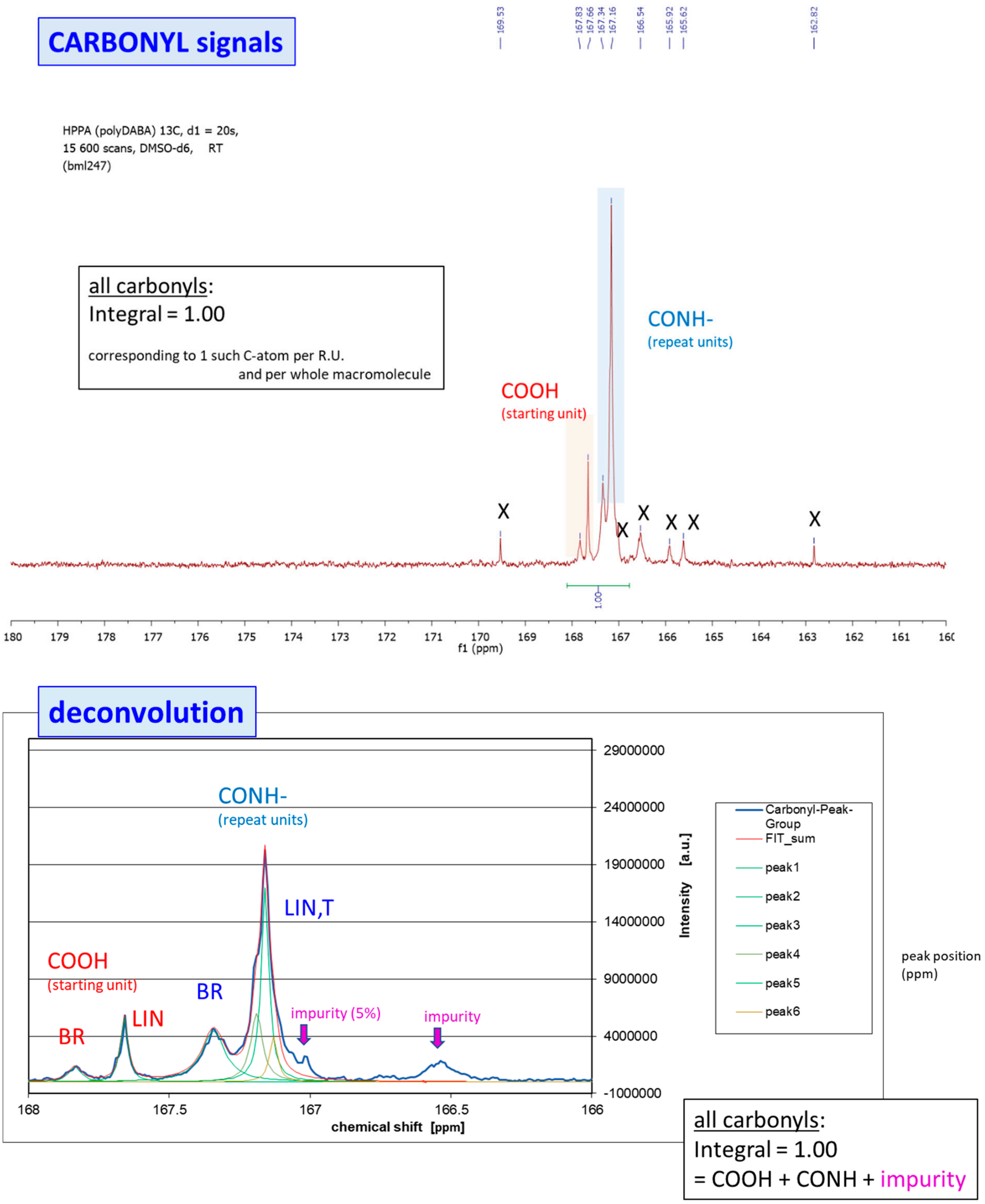

Figure S7. *Carbonyl peaks in the* ^{13}*C-NMR spectrum of the HPPA macro-initiator (top), and the deconvolution (bottom) of their signals (the results are quantified below in Table S2); the component fit functions called "peak 1" to "peak6" are numbered from left (high ppm) to right (low ppm); the sum of the fit functions is shown in red color, while the curve of the original spectrum is dark blue.*

__Table S2.__ Results of deconvolution of the Carbonyl peak region.

**name of component peak**	**assignment**	**peak position (ppm)**	**intensity parameter** of Lorentz function (-)	**width parameter** of Lorentz function (-)	**area below component peak (a.u.)**	**fraction of area – prominence of component peak (%)**
peak1	COOH, BR	167.835	1.30E+06	0.055	1.12E+05	4.03
peak2	COOH, LIN	167.66	5.70E+06	0.032	2.87E+05	10.27
peak3	CONH, BR	167.343	4.45E+06	0.1	6.99E+05	25.05
peak4	CONH, LIN,T	167.19	6.00E+06	0.05	4.71E+05	16.89
peak5	-“-“-	167.16	1.70E+07	0.033	8.81E+05	31.58
peak6	-“-“-	167.13	4.00E+06	0.033	2.07E+05	7.43
impurity	impurity	167.02	n.a.	n.a.	1.32E+05	4.75

Assignment of deconvoluted peaks:

branching units (BR): *peak 1 + peak3*

linear units (LIN): *peak2 + peak4 + peak5 + peak6*

the impurity at 167.02 ppm was included into the group integral (top image) of 1.00 but it was not fitted; its partial area was included into the deconvolution analysis in Table S2.

the impurity at 165.55 ppm was not part of the group integral of 1.00 (top image) and also was not included into the deconvolution analysis.

Area analysis and prominence of structural groups:

COOH = 4.03% + 10.27% = 14.30%

CONH = 25.05% + 16.89% + 31.58% + 7.43% = 25.05% + 55.89% = 80.94%

impurity = 4.76%

=> ratio of ‘Start’ repeat units (with COOH) to the remaining ones (linear, branching and terminal: LIN, BR, T) is:

***number*(Start) / *number*(BR+LIN+T)** = *area*(all COOH) / *area*(all CONH) =

= 14.30% / 80.94% = **1 / 5.66**

=> polymerization degree (P_n) = *number*(Start) / *number*(BR+LIN+T) = 1 +5.66 = **6.66**

as the COOH-containing repeat unit (‘Start’) is only one per macromolecule (the **accuracy is moderate**, as the signal of COOH units is weak).

ratio LIN/BR in case of **‘Start’ (COOH) units:** (no COOH-T units, as eventual unreacted monomer was removed)

StartLIN / StartBr = 10.27% / 4.03% = **2.55 / 1** (this concerns only the Start units)

remaining repeat units (not very accurate):

(LIN+T)/Br = 55.89 / 25.05 = **2.23 / 1** (LIN+T = *peak4 + peak5 + peak6*)

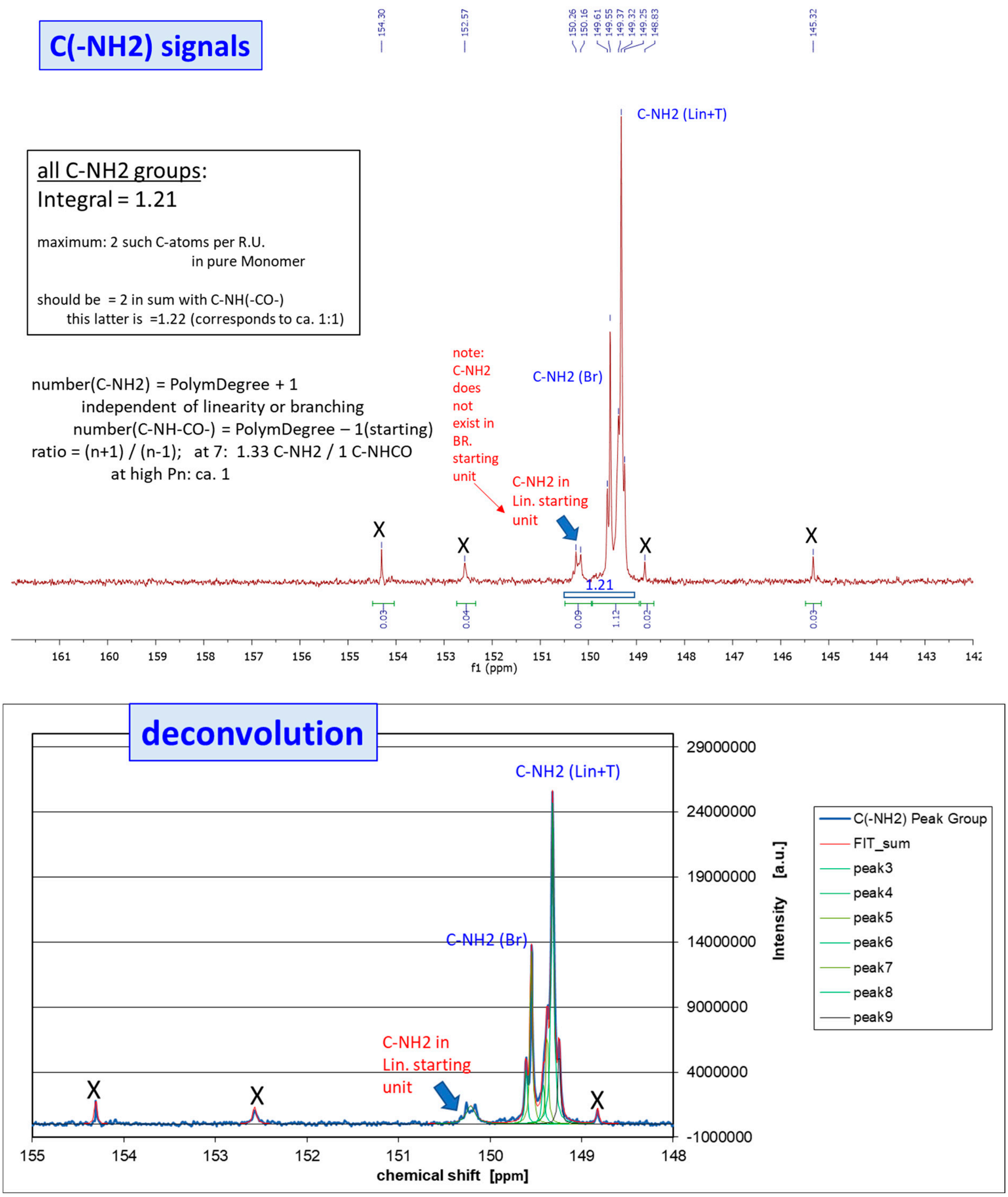

Figure S8. *C(-NH$_2$) peaks in the ^{13}C-NMR spectrum of the HPPA macro-initiator (top), and the deconvolution (bottom) of their signals (the results are quantified below in Table S3); the component fit functions called "peak 3" to "peak9" are numbered from left (high ppm) to right (low ppm); the sum of the fit functions is shown in red color, while the curve of the original spectrum is dark blue.*

Table S3. *Results of deconvolution of the C(-NH_2) peak region.*

**name of component peak**	**assignment**	**peak position (ppm)**	**intensity parameter** of Lorentz function (-)	**width parameter** of Lorentz function (-)	**area below component peak (a.u.)**	**fraction of area – prominence of component peak (%)**
peak3	'StartLIN' unit	150.211	1.40E+06	0.12	2.64E+05	8.11
peak4	T units	149.607	4.30E+06	0.03	2.03E+05	6.23
peak5	-"-"-	149.55	1.33E+07	0.03	6.27E+05	19.28
peak6	LIN (+ ev. T) units	149.42	3.00E+06	0.04	1.88E+05	5.80
peak7	-"-"-	149.376	7.00E+06	0.033	3.63E+05	11.16
peak8	-"-"-	149.317	2.55E+07	0.033	1.32E+06	40.65
peak9	-"-"-	149.245	5.50E+06	0.033	2.85E+05	8.76

Assignment of deconvoluted peaks:

Start linear units (StartLIN): *peak3*

Terminal units (T): *peak4* + *peak5*

Linear (ev. Terminal) units: *peak6* +*peak7* + *peak8* + *peak9*

Integral of the whole C-NH2 peak region = 1.21 =

= StartLIN + LIN + T (BR and StartBR units do not possess any NH2 group, but only NH-CO-).

Intensities from area % in Deconvolution:

startLIN = **8.11%**

BR (and StartBR) = 0%: no C-NH2 signals on such repeat units

T = 6.23% + 19.28% = **25.51%**

LIN = 5.80% + 11.16% + 40.65% + 8.76% = **66.37%**

Branching is not possible to evaluate from this spectral region:

BR and StartBR are invisible;

P_n also **impossible to determine:**

not all Start (StartBR) and none of the internal BR units are visible.

A prominent presence of the LIN units can be seen:

LIN / T = ca. 2.60 / 1

(LIN+StartLIN) / T = ca. 2.92 / 1 .

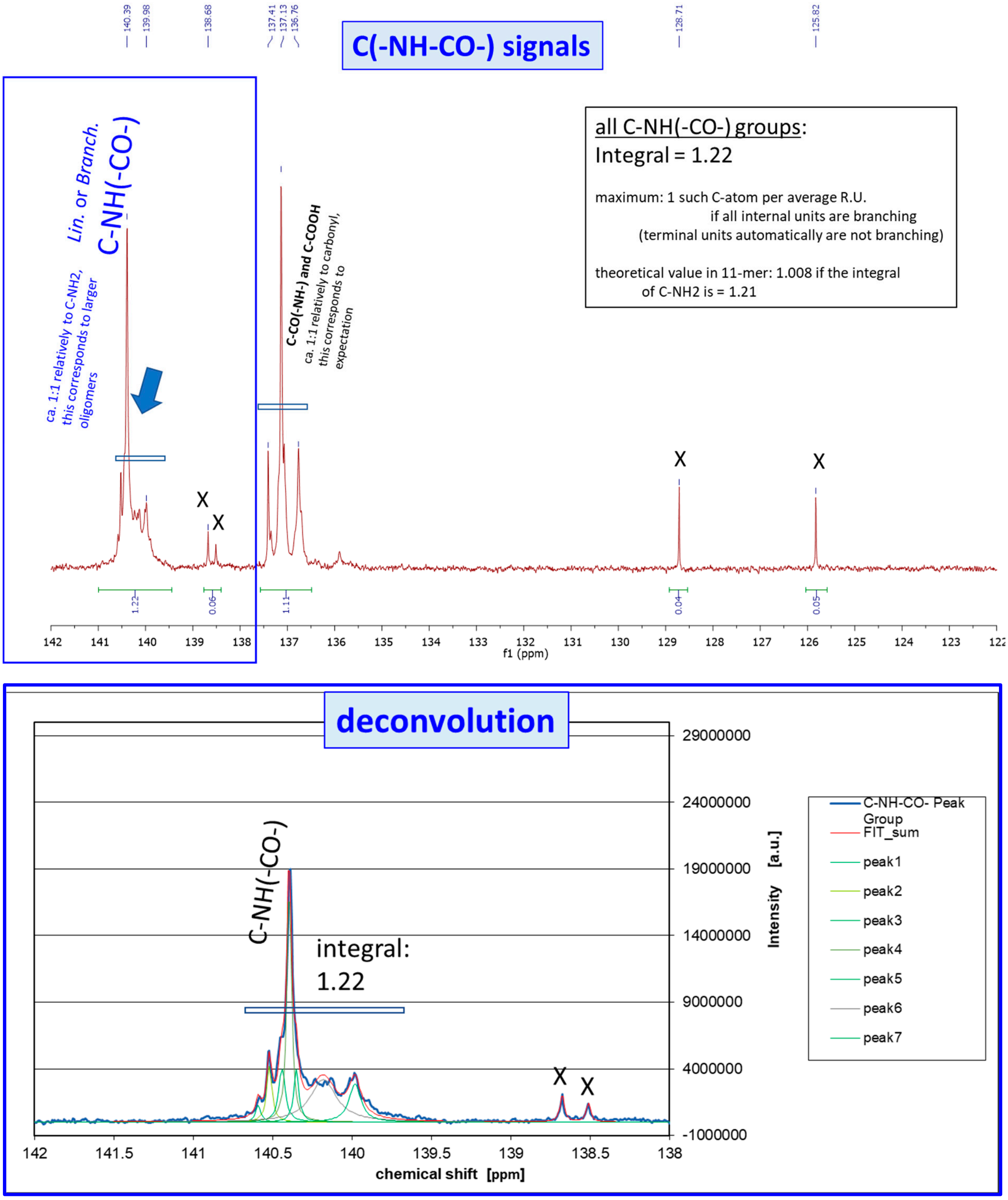

Figure S9. *C(-NH-CO-) peaks in the ^{13}C-NMR spectrum of the HPPA macro-initiator (top), and the deconvolution (bottom) of their signals (the results are quantified below in Table S4); the component fit functions called "peak 1" to "peak7" are numbered from left (high ppm) to right (low ppm); the sum of the fit functions is shown in red color, while the curve of the original spectrum is dark blue.*

Table S4. *Results of deconvolution of the C(-NH-CO-) peak region.*

**name of component peak**	**assignment**	**peak position (ppm)**	**intensity parameter of Lorentz function (-)**	**width parameter of Lorentz function (-)**	**area below component peak (a.u.)**	**fraction of area – prominence of component peak (%)**
peak1	C-NH-CO- signal	140.59	1.30E+06	0.04	8.17E+04	2.50
peak2	C-NH-CO- signal	140.52	4.20E+06	0.04	2.64E+05	8.10
peak3	C-NH-CO- signal	140.44	4.00E+06	0.05	3.14E+05	9.64
peak4	C-NH-CO- signal	140.395	1.80E+07	0.033	9.33E+05	28.63
peak5	C-NH-CO- signal	140.35	4.00E+06	0.04	2.51E+05	7.71
peak6	overlaid multiple signals and/or impurity	140.18	3.20E+06	0.2	1.01E+06	30.85
peak7	Start, or impurity	139.98	2.90E+06	0.09	4.10E+05	12.58

Assignment of deconvoluted peaks:

The peaks are grouped and difficult to assign.

The Integral of the whole C-NH2 peak region is equal to 1.22,

1.01 would be expected in theory, in view of the excessively intensive C-NH_2 signal, of the polymerization degree and the combination of Formulas (6) and (7) further above, which gives the following ratio (independent of degree of branching):

$$number(\text{C-NH2})/number(\text{C-NH-CO-}) = (P_n + 1)/(P_n - 1)$$

which would be 1.222 / 1 for 10-mers (number average polymerization degree as determined by MALDI-TOF).

The *peak6* and *peak7* (shoulder structures at lower ppm values of the main composite peak) might be partly attributed to impurities.

Branching evaluation **cannot be done** using this peak group, due to too close overlapping of signals.

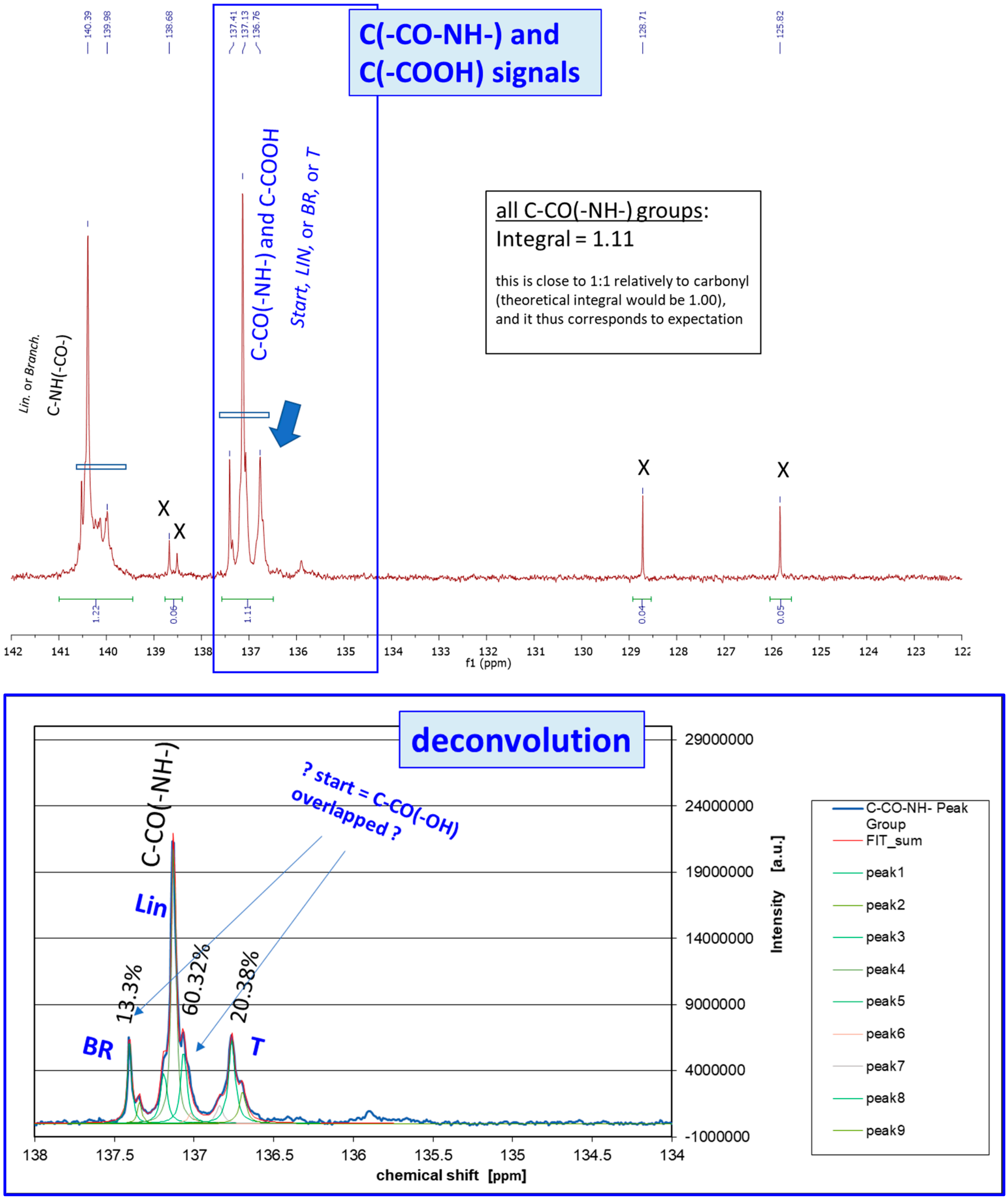

Figure S10. *C(-CO-NH-) and C(-COOH-) peaks in the ^{13}C-NMR spectrum of the HPPA macro-initiator (top), and the deconvolution (bottom) of their signals (the results are quantified below in Table S5); the component fit functions called "peak 1" to "peak9" are numbered from left (high ppm) to right (low ppm); the sum of the fit functions is shown in red color, while the curve of the original spectrum is dark blue.*

Table S5. *Results of deconvolution of the C(-CO-NH-) and C(-COOH-) peak region.*

name of component peak	assignment	peak position (ppm)	intensity parameter of Lorentz function (-)	width parameter of Lorentz function (-)	area below component peak (a.u.)	fraction of area – prominence of component peak (%)
peak1	BR units	137.405	6.70E+06	0.03	3.16E+05	10.4
peak2	-“-“-	137.342	1.70E+06	0.033	8.81E+04	2.9
peak3	LIN units	137.19	3.80E+06	0.05	2.98E+05	9.83
peak4	-“-“-	137.128	2.10E+07	0.033	1.09E+06	35.83
peak5	-“-“-	137.065	5.50E+06	0.045	3.89E+05	12.8
peak6	-“-“-	137.02	8.00E+05	0.045	5.65E+04	1.86
peak7	T units	136.84	1.40E+06	0.05	1.10E+05	3.62
peak8	-“-“-	136.763	6.40E+06	0.05	5.03E+05	16.55
peak9	-“-“-	136.695	2.40E+06	0.05	1.88E+05	6.21

Assignment of deconvoluted peaks:

Branching units (BR): *peak 1* + *peak2* (13.3% of the area of the peak group)

Linear units (LIN): *peak3* + *peak4* + *peak5* + *peak6* (60.32% of the area of the peak group)

Terminal units (T): *peak7* + *peak8* + *peak9* (20.38% of the area of the peak group)

the signals of Start units are not resolved and likely overlapped by BR and LIN signals

The Integral of the whole *C(-CO-NH-)* and *C(-COOH-)* peak region is equal to 1.11 ,

which is close to the theoretical value, which should be 1.00 (like the integral of the carbonyl groups themselves)

Area analysis and prominence of structural groups:

The ratios of the areas of deconvoluted sub-groups of *C(-CO-NH-)* and *C(-COOH-)* signals are:

T / BR / LIN = ca. 1 / 0.65 / 3 .

BR / LIN / T = ca. 0.65 / 3 / 1 .

The signals of the StartLIN and StartBR groups appear to be overlapping with BR, LIN and T signals.

The result is in relatively good agreement with an average 10-mer molecule, as suggested by MALDI-TOF, which contains 1 StartLIN unit, 1 BR unit, 6 LIN units, and 2 T units (according to the precise value of the above ratio, there should be 1.3 BR units and 5 LIN units) .

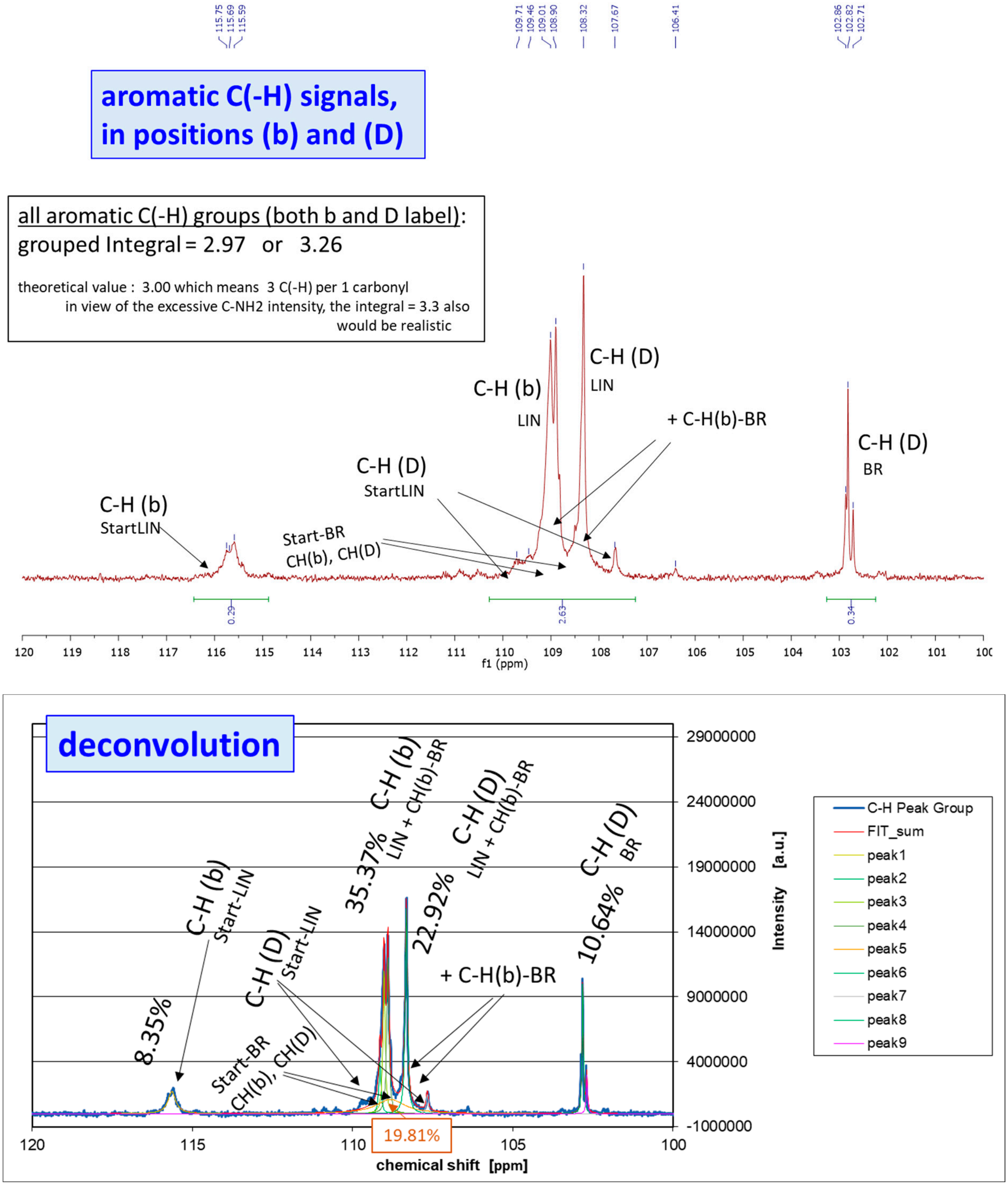

Figure S11. *Aromatic C(-H) peaks (atom positions (b) and (D)) in the* ^{13}*C-NMR spectrum of the HPPA macro-initiator (top), and the deconvolution (bottom) of their signals (the results are quantified below in Table S6); the component fit functions called "peak 1" to "peak9" are numbered from left (high ppm) to right (low ppm); the sum of the fit functions is shown in red color, while the curve of the original spectrum is dark blue.*

Table S6. *Results of deconvolution of the Aromatic C(-H) peaks (atom positions (b) and (D)).*

**name of component peak**	**assignment**	**peak position (ppm)**	**intensity parameter of Lorentz function (-)**	**width parameter of Lorentz function (-)**	**area below component peak (a.u.)**	**fraction of area – prominence of component peak (%)**
impurity	impurity	109.6	n.a.	n.a.	2.79E+05	2.91
peak1	Start, C(-H) (b)	115.64	1.70E+06	0.3	8.01E+05	8.35
peak2	LIN, C(-H) (b)	109.15	4.00E+06	0.08	5.03E+05	5.24
peak3	-“-“-	109.02	1.10E+07	0.08	1.38E+06	14.41
peak4	-“-“-	108.9	1.20E+07	0.08	1.51E+06	15.72
peak5	diffuse signal intensity	108.82	1.10E+06	1.1	1.90E+06	19.81
peak6	LIN, C(-H) (D)	108.326	1.60E+07	0.08	2.01E+06	20.96
peak7	-“-“-	107.67	1.50E+06	0.08	1.88E+05	1.96
peak8	BR, C(-H) (D)	102.82	1.00E+07	0.05	7.85E+05	8.18
peak9	-“-“-	102.69	3.00E+06	0.05	2.36E+05	2.46

Assignment of deconvoluted peaks:
Start-LIN units, C(-H) (b): *peak 1*
LIN units, C(-H) (b): *peak2 + peak3 + peak4*
LIN units, C(-H) (D): *peak6 + peak7*
overlapping with the region LIN{C(-H) (b)} + LIN{C(-H) (D)}:
StartLIN{C(-H)(D)} + BR{C(-H)(b)} + expected weak signals of StartBR
these signals are likely the main source of the ‘diffuse signal’ peak of the deconvolution (*peak5* in the above Table).

BR units, C(-H) (D): *peak8 + peak9*

Area analysis and prominence of structural groups:
StartLIN units: area sized 8.35% is visible (C(-H) (b) signals)
overlapped (hidden) StartLIN{C(-H) (D)} = 8.35% / 2 (according to chemical formula) = 4.17%
StartLIN total = 8.35% + 4.17% = 12.52%
remaining C(-H) signals of HPPA (except the impurity signal),
sum from above table: 88.74%
=> *remaining repeat units* / StartLIN = 88.74% / 12.52% = 6.75 / 1
polymerization degree: P_n = ca. 7.75 (= above ratio + 1 starting unit)

Branching analysis:
C(-H) signals in (D) position:
LIN(D) = 20.96% + 1.96% = 22.92%
BR(D) = 8.18 + 2.46 = 10.64%

LIN/BR = LIN(D)/BR(D) = 22.9%/10.64% = **2.15**

LIN(D) + Start-LIN(D) = 22.92% + 4.17% = 27.1% =>
(StartLIN+LIN)/BR = ca. (LIN(D)+ Start-LIN(D)) / BR(D) = 27.1% / 10.6% = **2.55**

^{1}H-NMR spectroscopy

The *Figures S12* and S*13* (zoom on aromatic signals) show the ^{1}H-NMR spectrum of the HPPA macro-initiator (recorded in CD_2Cl_2 solution), and the assignment of its resonance signals. In ^{1}H-NMR, the signals are more split and are more difficult to separate. It can be nevertheless recognized, that the ratio of the signals of 'Start' units to the remaining ones suggests a degree of polymerization

***P*$_n$ = ca. 8.8** (close to both ^{13}C-NMR and MALDI-TOF), as well as

one branching unit in such a molecule (see comment below the zoomed view of the aromatic region in Figure S12).

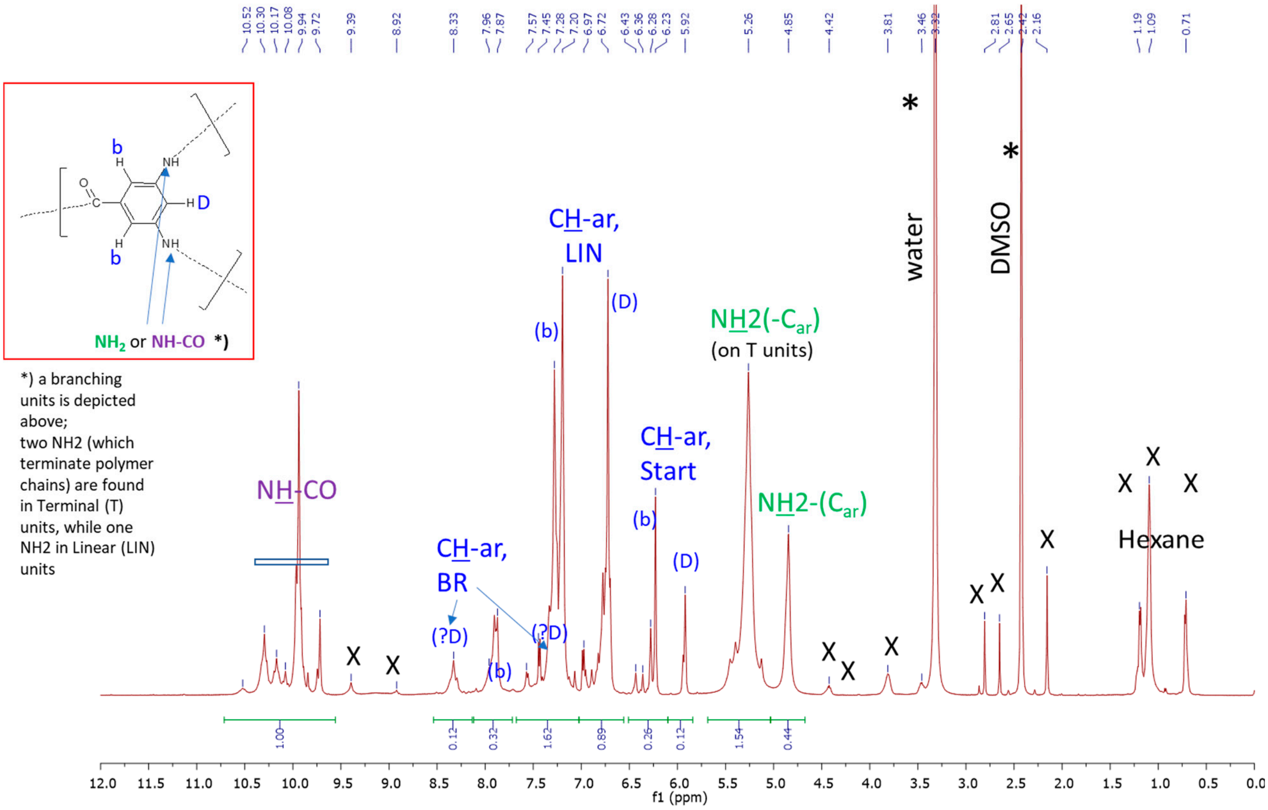

Figure S12. *^{1}H-NMR spectrum of the HPPA macro-initiator, recorded in DMSO-d6 solution, and the assignment of its resonance signals; abbreviation "LIN" means "linear repeat units", "BR" means "branching (dendritic) repeat units", "T" means "terminal (outermost) repeat units", "Start" means "starting repeat unit" (the first unit of the macromolecule with COOH group still intact).*

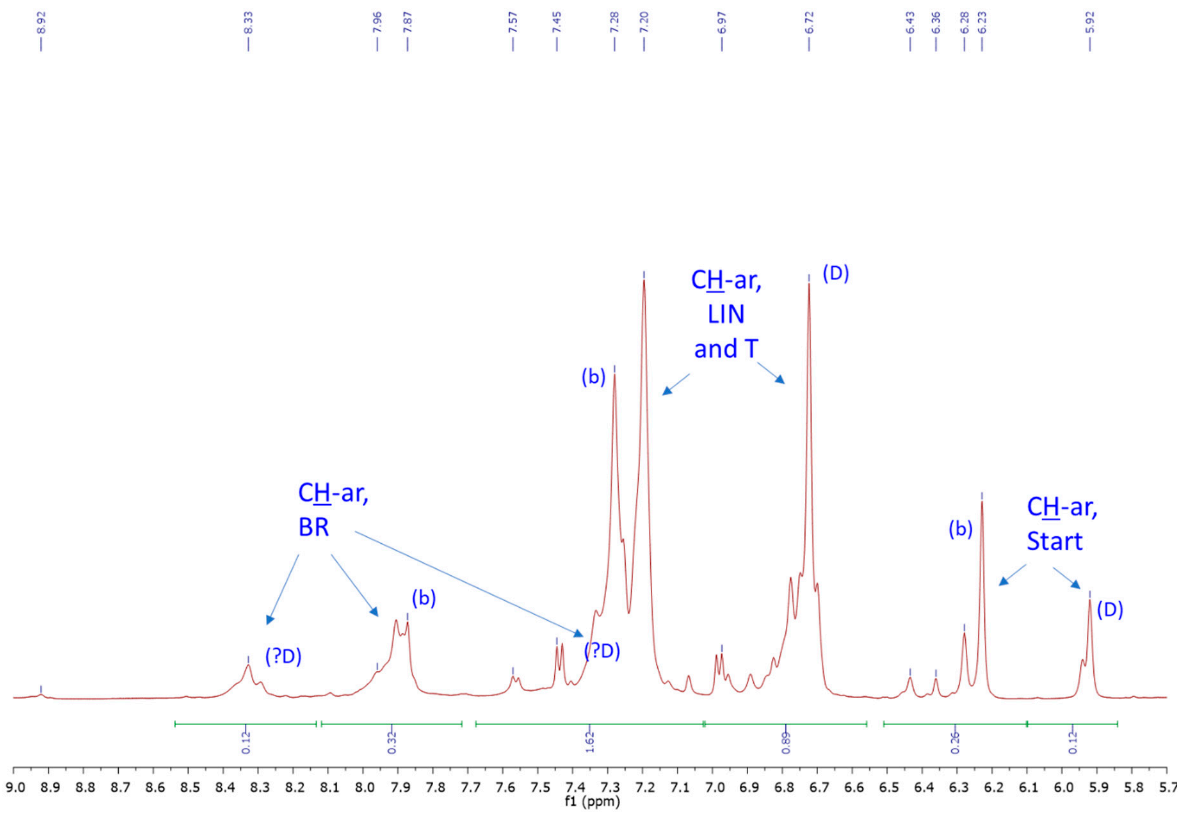

Figure S13. *^{1}H-NMR spectrum of the HPPA macro-initiator, zoom on the aromatic region.*

BR units: {C(-H) (b) + C(-H) (D) signals} = 0.12 + 0.32 = 0.44 area units

LIN and T units: {C(-H) (b) + C(-H) (D) signals} = 1.62 + 0.89 = 2.51 area units

Start units: {C(-H) (b) + C(-H) (D) signals} = 0.26 + 0.12 = 0.38 area units

(BR + LIN + T) / Start = (0.44 + 2.51) / 0.38 = 2.95 / 0.38 = 7.76 / 1

because there is just one Start unit per molecule:

$\boldsymbol{P_n}$ **=** 7.76 + 1 = **ca. 8.8**

because the Integrals of Start and BR units are ca. the same, there is in average **1 BR unit** (more precisely 1.16 units) **per 9-mer** (8.8-mer) **molecule**.

4. PCL-HPPA copolymer: NMR Analysis of its structure.

The primary structure of the highly branched PCL-HPPA copolymer (HPPA-rich sample sample PCL-0.33% HPPA - 5 kDa) was analyzed by means of ^{13}C-NMR (Figures S14–S16) and ^{1}H-NMR (Figures S17–S19) spectroscopy. Of especial interest was the degree of grafting and hence the number of grafted chains per HPPA macro-initiator molecule. Also evaluated was the molar ratio of the repeat units of the components HPPA and PCL, which together with the functionality makes possible to calculate the idealized average length of the grafted chains (if assuming that no free PCL chains were formed).

<u>^{13}C-NMR</u>:

The signals of grafted HPPA can be clearly recognized, but are too weak and noisy to be accurately integrated (see zoom in (Figure S15). Only a rough analysis of grafting is possible.

The spectrum of the grafted HPPA in the copolymer (also according to simulation) is markedly different from the one of neat HPPA and confirms a high degree of grafting. The signals were possible to assign.

The C(-NH_2) signal of carbon atoms in the neighborhood of unreacted NH_2 groups is well visible and distinguishable. It's fairly low intensity **very roughly suggests**, that **ca. 50–70% of the original amino groups did react** (disappear and start HPPA-bonded PCL chains) during the reaction with PCL.

The C(-H)(b) signal of repeat units, which did not react, also is visible but is very weak.

<u>^{1}H-NMR</u>:

In the ^{1}H-NMR spectrum (Figures S17–S19), the quality of the aromatic signals (at least in the case of the sample PCL-0.33% HPPA - 5 kDa) is much better than in ^{13}C-NMR.

<u>Summary of analysis results</u>:

<u>Degree of grafting</u>:

The aromatic signals of the grafted copolymer display a simple structure. The peak positions also are distinctly different from neat HPPA, which suggests a high degree of grafting (also according spectrum simulation)

72% of the aromatic signals (Integrals) can be assigned to grafted LIN or T groups which should yield nearly identical signals. LIN (incl. StartLIN) and T groups are very prominent in the HPPA molecule: together they represent nearly 90% of the repeat units.

28% of signal intensity corresponds to other, non-grafted or branching (BR) repeat units. Ca. 12% must be the branching units (which carry no NH2 groups): average 1.3 BR units per average 10.5-mer HPPA molecule (hence 12%) were found by structural analysis of the macro-initiator.

16% of repeat units (remaining from 28% of the non-grafting units) hence correspond to such ones, which were able of grafting (possessed NH2 groups) but stayed intact.

100*16/(16+72) = **<u>82% of NH2 groups hence have reacted</u>** (10-mer: ca. 9 out of 11 amino groups).

Length of the grafted chains:

The ratio of Integrals CH_{ar} / CH_2-O- (see Figure S17) yields the length of an average PCL chain. The signals can be summarized as:

CH_{ar} = 0.0101 + 0.0102 + 0.01015(estimated CH(D) signal, which must have the same intensity like the CH(b1) and CH(b2) signals). Hence CH_{ar} = 0.03045. This corresponds to 3H atoms. Normalized to 2H, it is CH_{ar} NORM = 0.03045 * (2/3) = 0.0203

CH_2-O- = 2.00; it corresponds to 2 H atoms

The normalized ratio = 2.00 / 0.0203 = 98.5

means that ca. 99 PCL repeat units occur per one per HPPA repeat unit. A HPPA 10-mer molecule has 11 amino groups of which 82% (9.02 groups) are grafted (according to ^{1}H-NMR), as well as one graftable COOH group which is reactive and not sterically shielded (and hence grafted). This means, that on an average HPPA decamer, there are 10 groups which are PCL-grafted and 10 HPPA repeat units. The CL/HPPA-monomer ratio hence also corresponds to the PCL chain length.

=> **99 PCL groups are grafted in average chains** (which are connected to 82% of available amino groups, and to the former COOH group).

The analyzed spectra are shown below.
The simulated NMR spectra, which helped with assignment were obtained from nmrdb.org on the webpage: http://www.nmrdb.org/new_predictor/index.shtml .

^{13}C-NMR spectroscopy

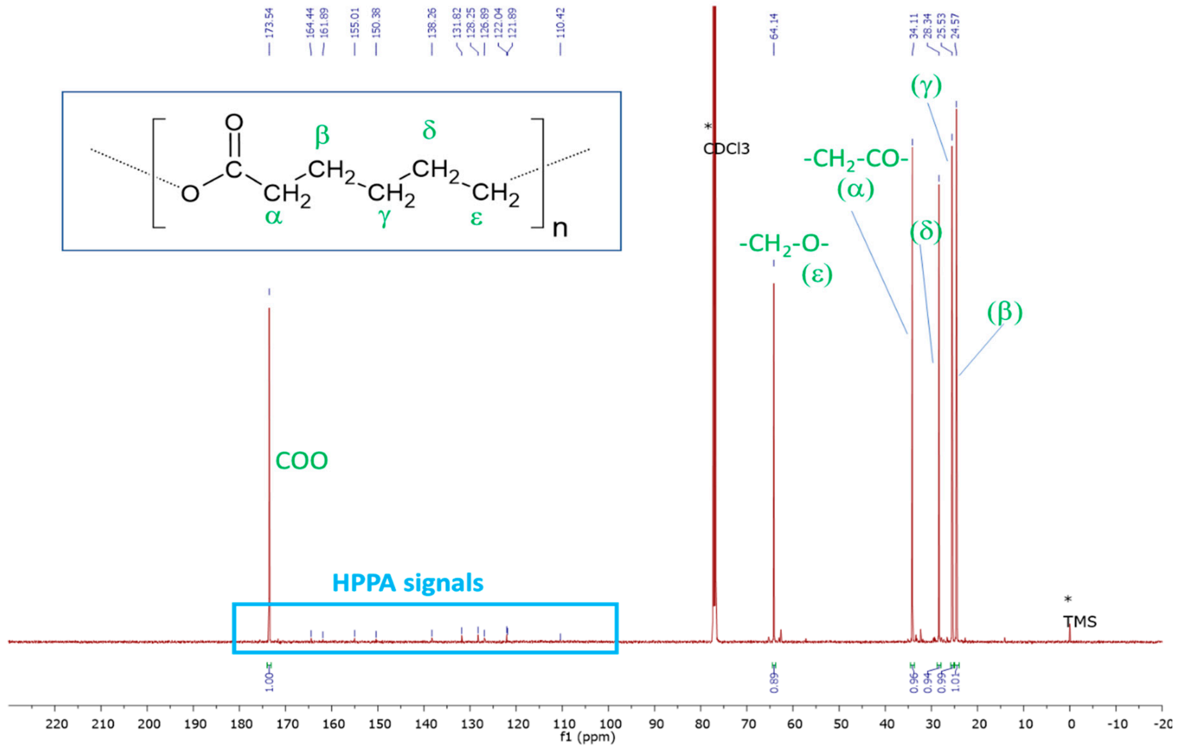

Figure S14. *^{13}C-NMR spectrum of the HPPA-PCL copolymer (in which the branched HPPA is grafted by PCL chains starting from its former NH_2 groups), namely of the sample PCL-0.33% HPPA - 5 kDa, recorded in CD_2Cl_2 solution, and the assignment of its PCL resonance signals. The aromatic signals of the HPPA core segment are barely distinguishable (they are highlighted by turquoise frame; a zoomed view is shown in the next following Figure).*

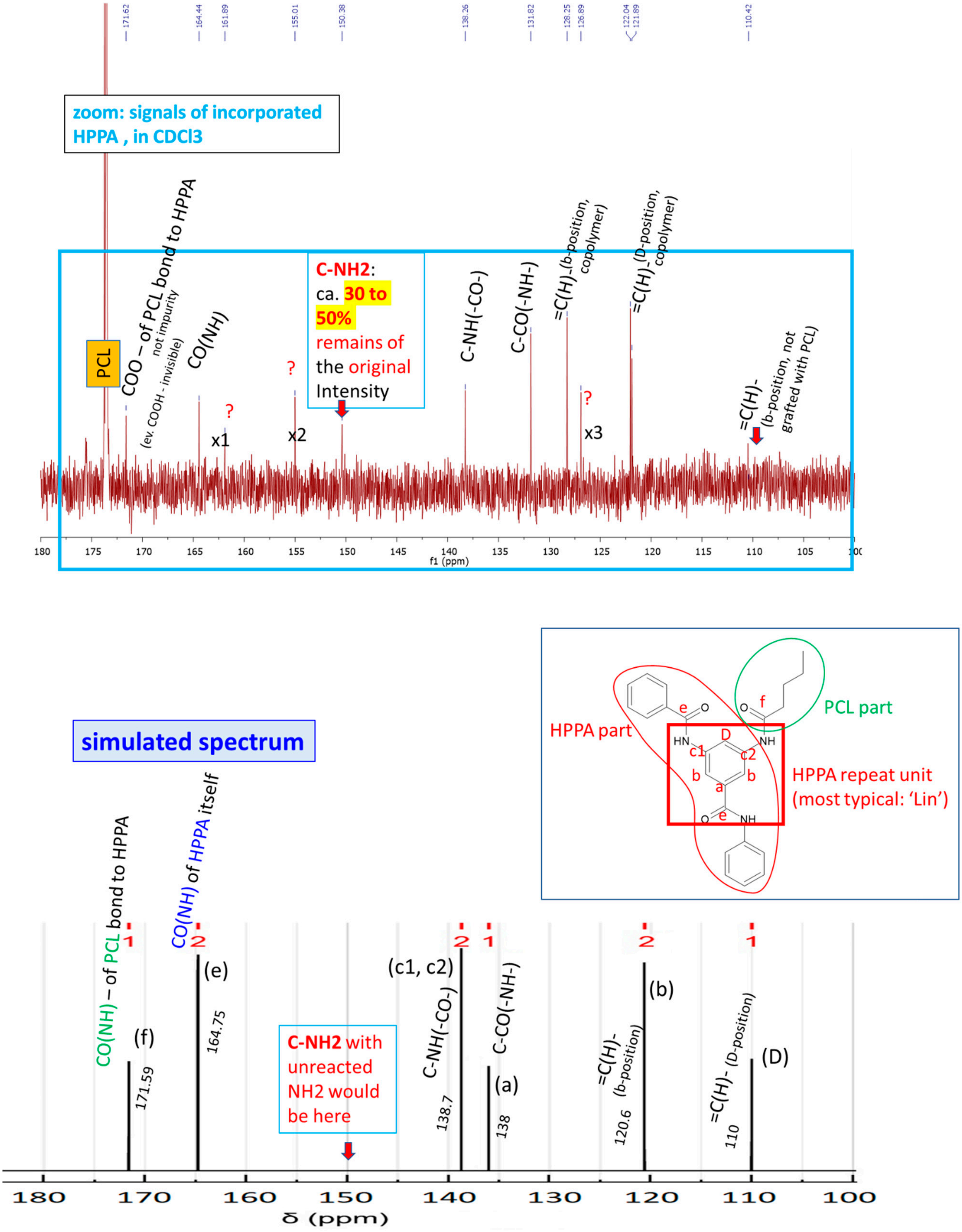

Figure S15. Zoomed view of the aromatic region of the ^{13}C-NMR spectrum of the HPPA-PCL copolymer, sample PCL-0.33% HPPA - 5 kDa, and the assignment of the resonance signals of grafted HPPA: (top): the zoomed spectrum itself; (bottom): simulated spectrum of a grafted HPPA repeat unit of LIN type (the signals of the adjacent groups are not shown in the simulated spectrum).

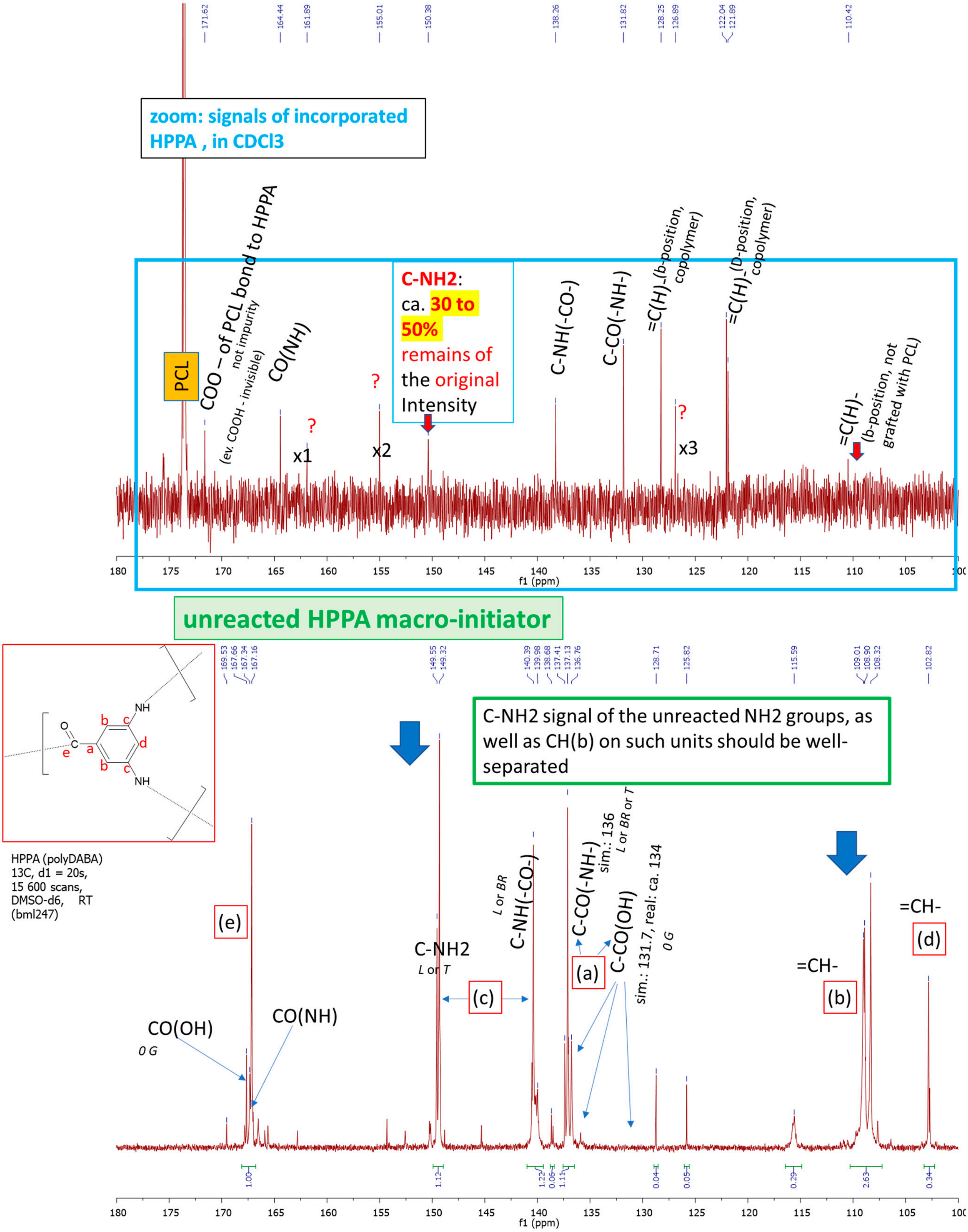

Figure S16. Zoomed view of the aromatic region of the ^{13}*C-NMR spectrum of the HPPA-PCL copolymer, sample PCL-0.33% HPPA - 5 kDa, and its comparison with the spectrum of neat HPPA; the C(-NH₂) signal is the most intense in the spectrum.*

^{1}H-NMR spectroscopy

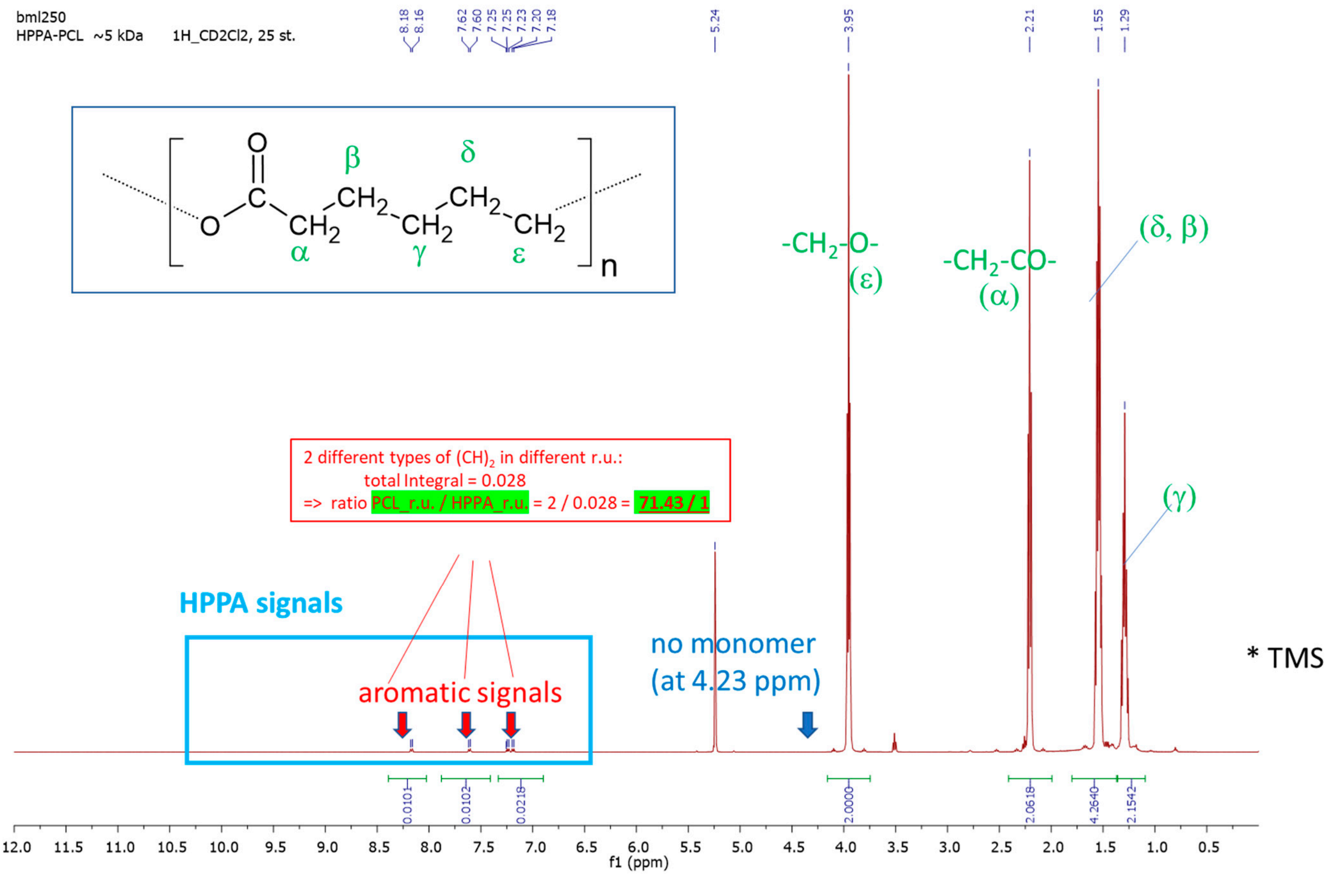

Figure S17. *^{1}H-NMR spectrum of the HPPA-PCL copolymer (in which the branched HPPA is grafted by PCL chains starting from its former NH_2 groups), namely of the sample PCL-0.33% HPPA - 5 kDa, recorded in CD_2Cl_2 solution, and the assignment of its PCL resonance signals. The aromatic signals of the HPPA core segment are barely distinguishable (they are highlighted by turquoise frame; a zoomed view is shown in the next following Figure).*

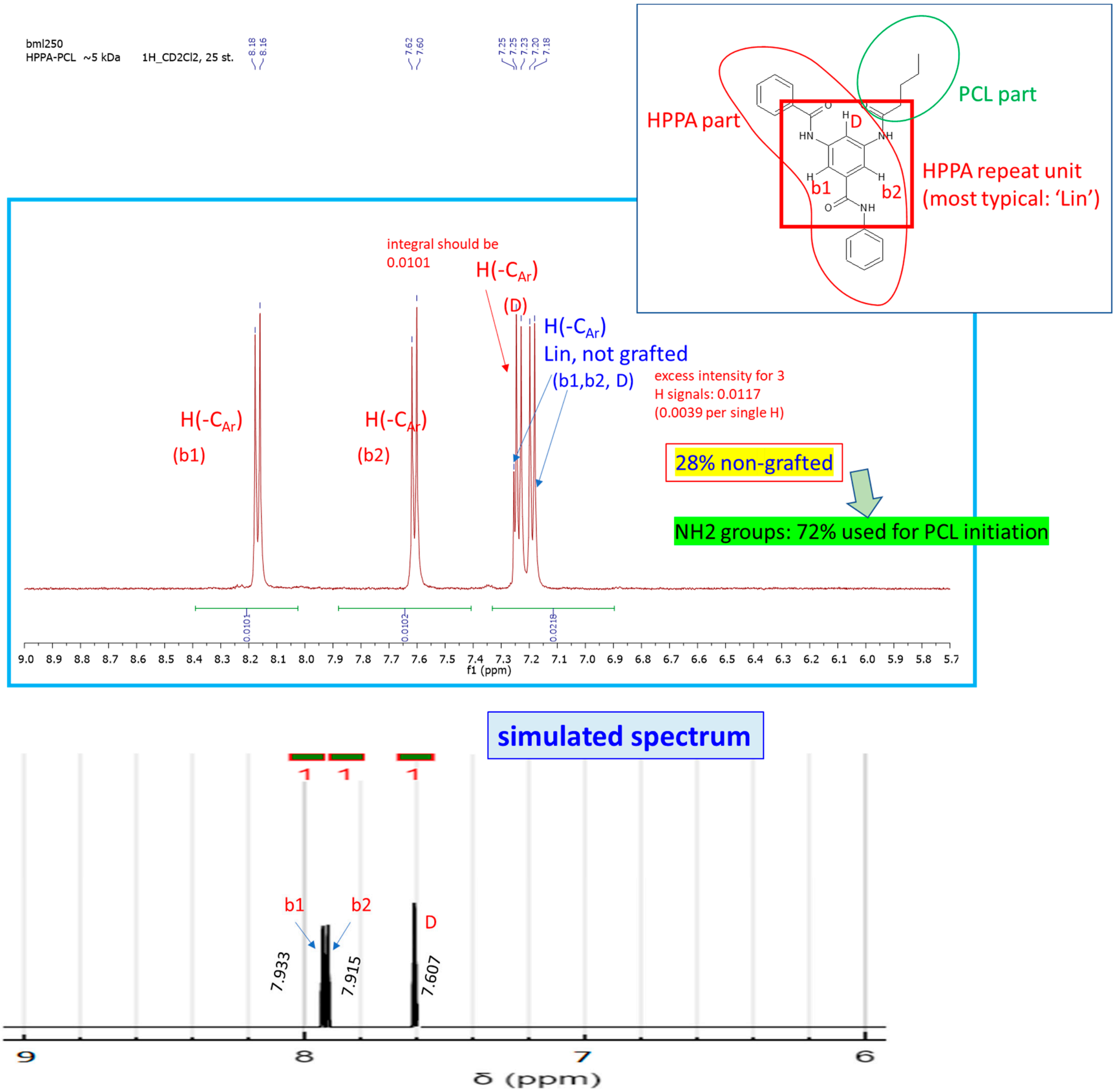

Figure S18. *Zoomed view of the aromatic region of the* ^{1}*H-NMR spectrum of the HPPA-PCL copolymer, sample PCL-0.33% HPPA - 5 kDa, and the assignment of the resonance signals of grafted HPPA: (top): the zoomed spectrum itself; (bottom): simulated spectrum of a grafted HPPA repeat unit of LIN type (the signals of the adjacent groups are not shown in the simulated spectrum). Note: completely grafted T units (which are less abundant than LIN) are expected to have nearly identical spectral signals like LIN units (due to very similar structural surrounding – see structure in the inlay image); the remaining partly grafted or non-grafted structures should yield separate resonance peaks.*

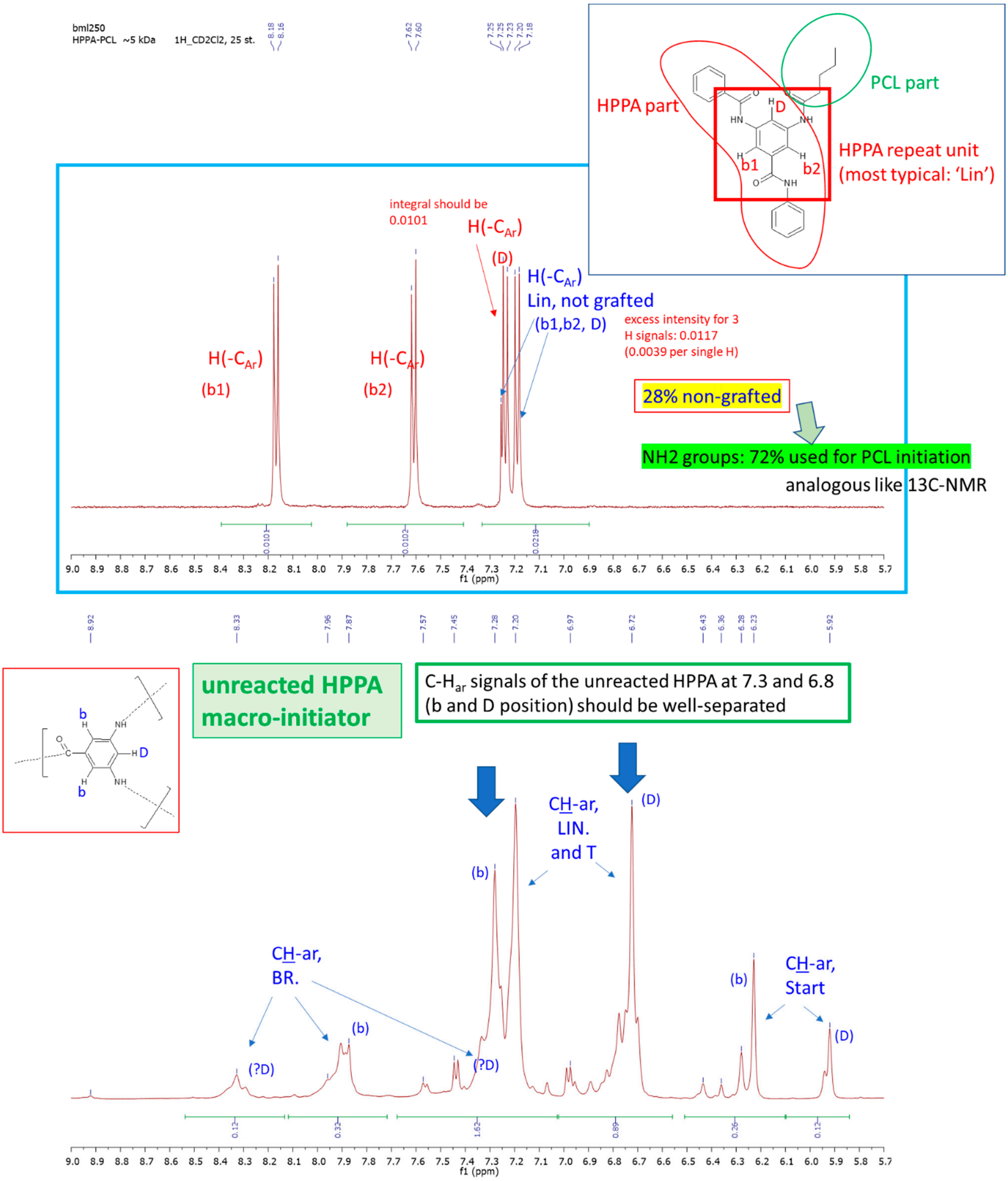

Figure S19. *Zoomed view of the aromatic region of the* [1]*H-NMR spectrum of the HPPA-PCL copolymer, sample PCL-0.33% HPPA - 5 kDa, and its comparison with the spectrum of neat HPPA; "b" and "D" signals of non-grafted LIN units should represent the most intense peaks of HPPA repeat units which did not react.*

5. ^{1}H NMR spectra of obtained PCL-HPPA copolymers, PCL linear and monomer.

The ^{1}H NMR spectra of HPPA-free (linear) PCL, of the prepared PCL-rich PCL-HPPA copolymers, as well as that of neat caprolactone monomer are shown below. The assignment was performed in the previous section (just above, on the example of the copolymer *PCL-0.33% HPPA - 5 kDa*).

a)

b)

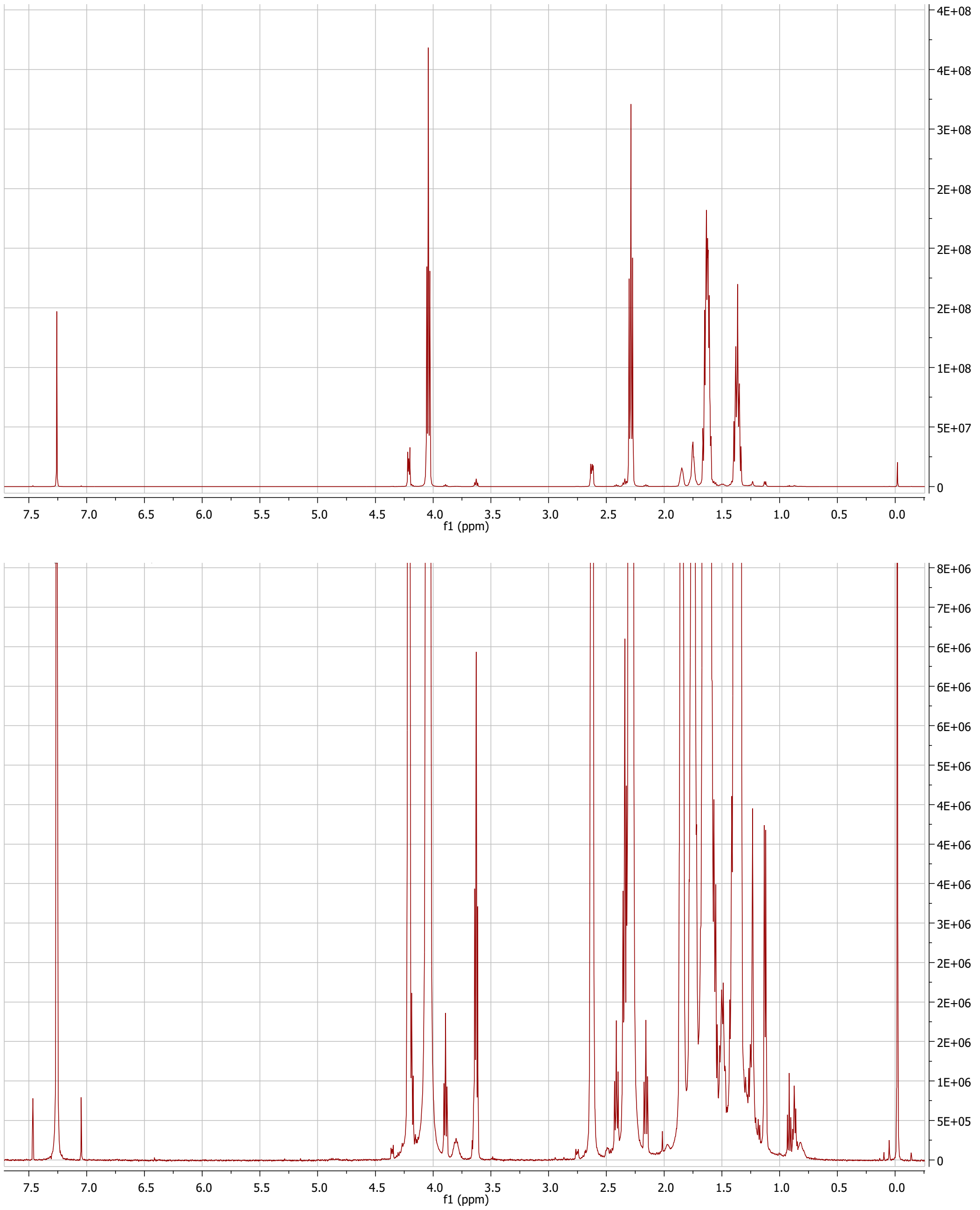

Figure S20. *PCL-linear: ^{1}H NMR spectrum: **a)** standard zoom, **b)** highly amplified.*

a)

b)

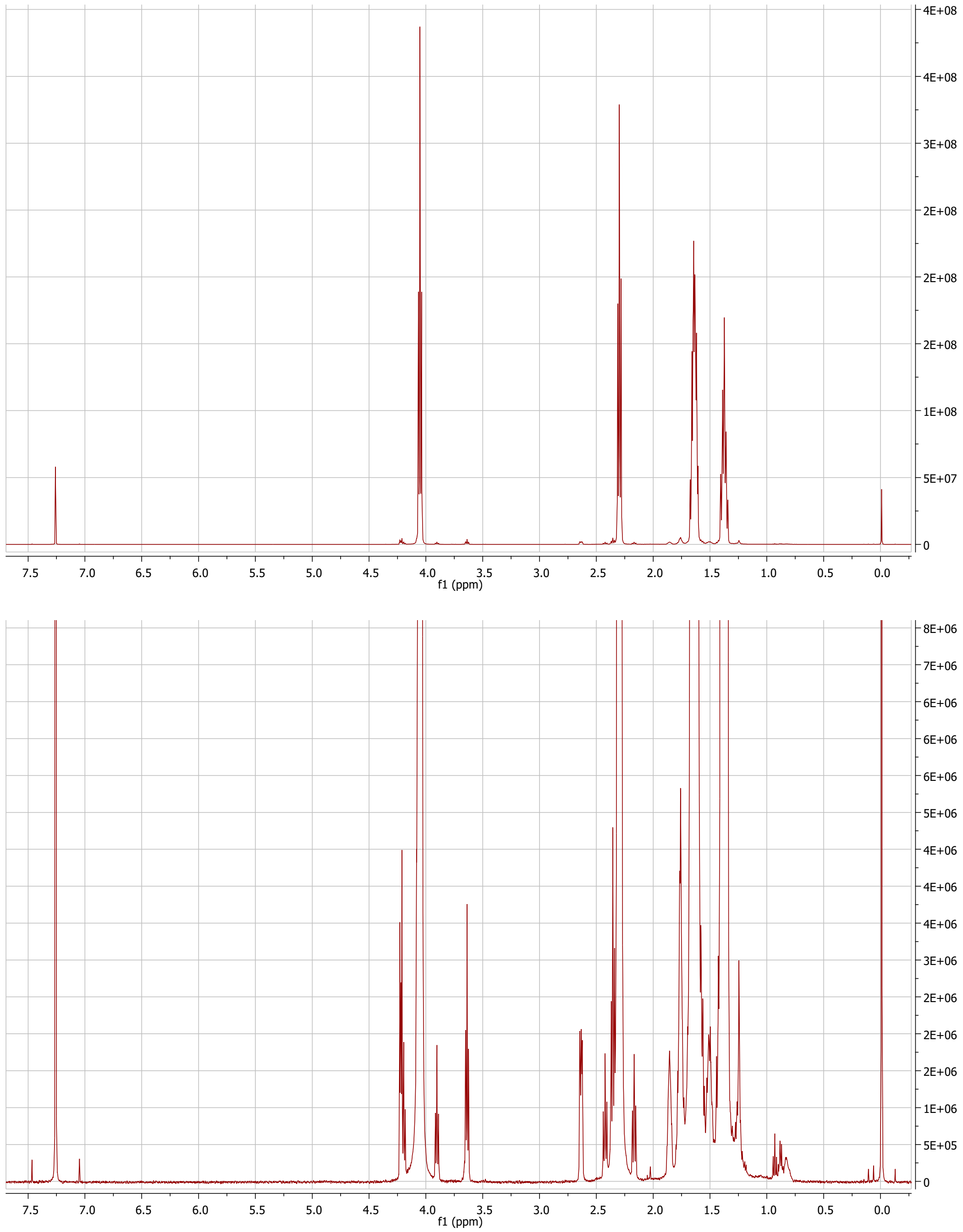

Figure S21. *PCL-0.033% HPPA ^{1}H NMR spectrum;* ***a)*** *standard zoom,* ***b)*** *highly amplified.*

a)

b)

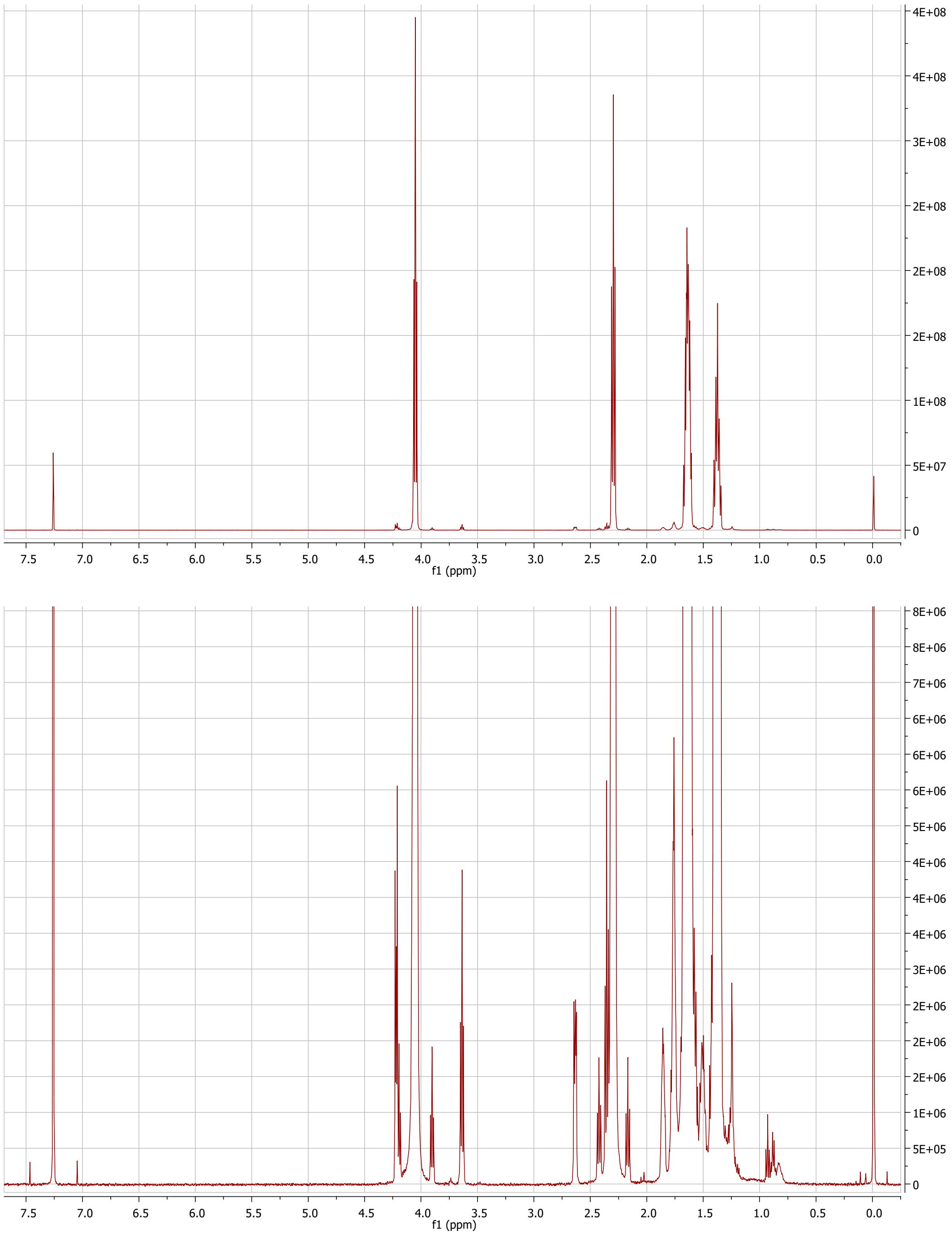

Figure S22. *PCL-0.067% HPPA ^{1}H NMR spectrum;* ***a)*** *standard zoom,* ***b)*** *highly amplified.*

a)

b)

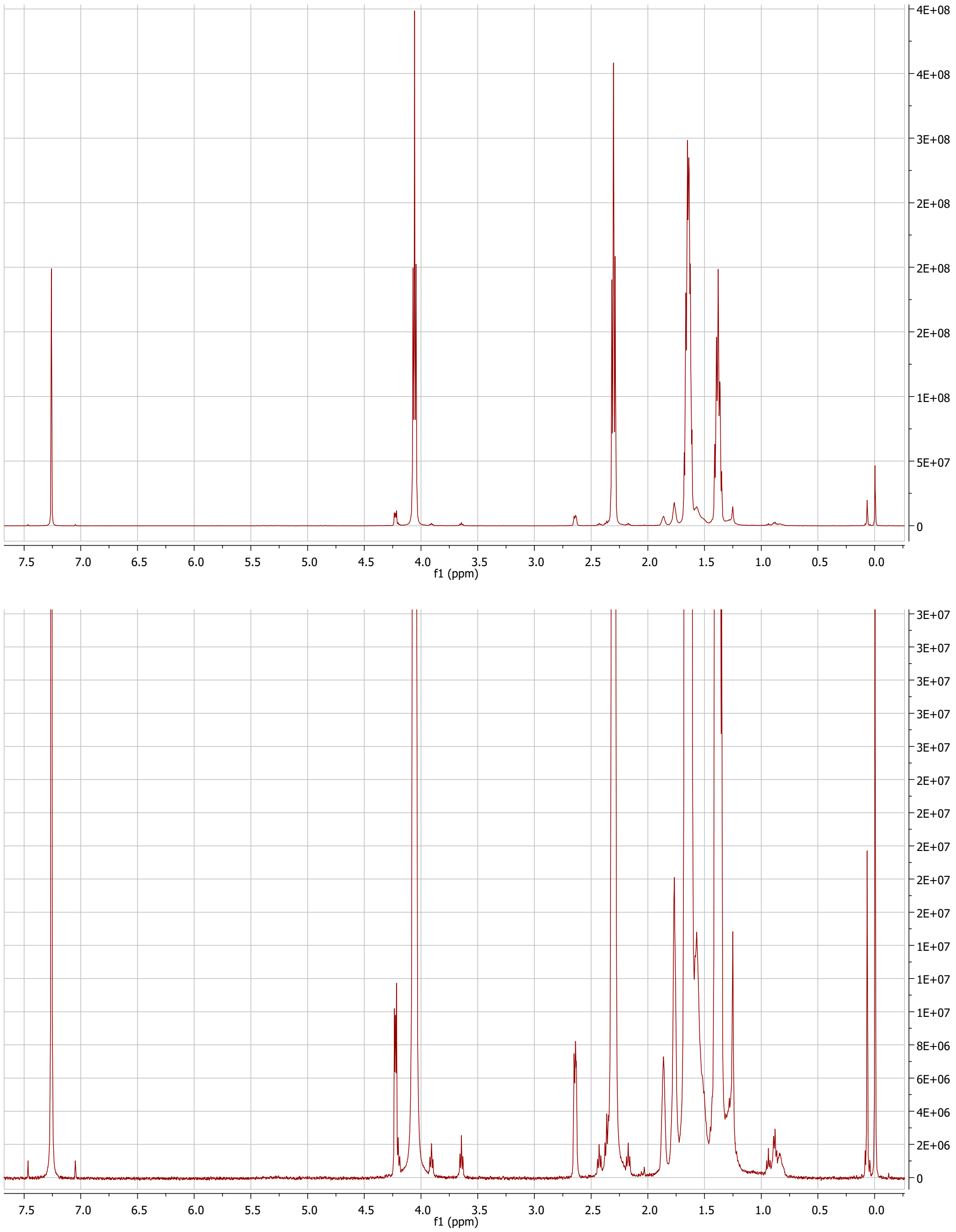

Figure S23. *PCL-0.13% HPPA* ^{1}*H NMR spectrum;* ***a)*** *standard zoom,* ***b)*** *highly amplified.*

a)

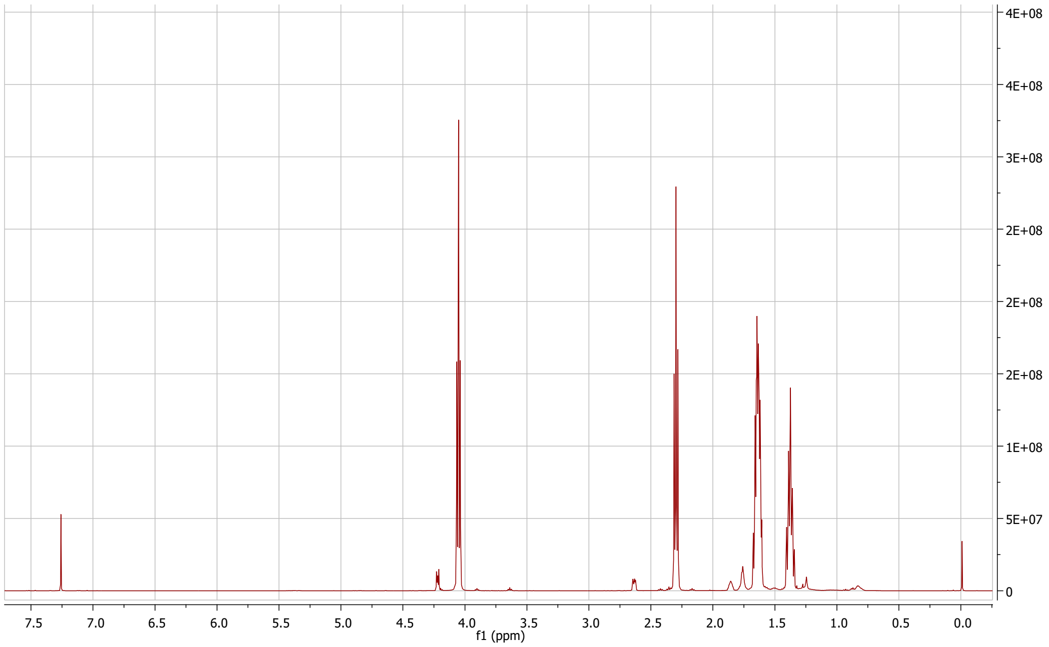

b)

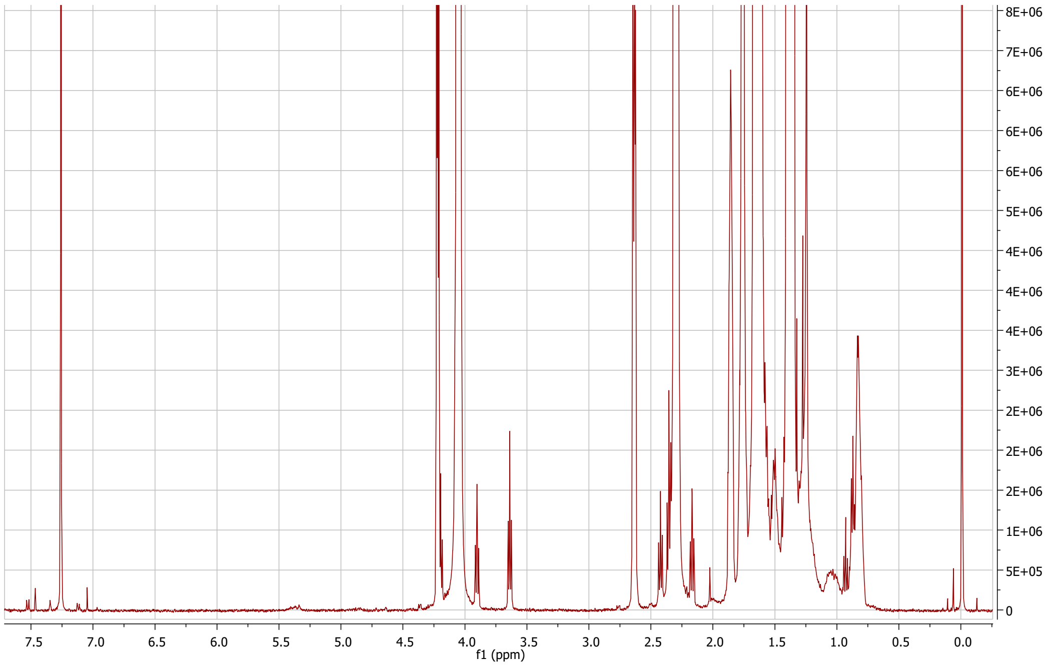

Figure S24. *PCL-0.33% HPPA ^{1}H NMR spectrum;* ***a)*** *standard zoom,* ***b)*** *highly amplified.*

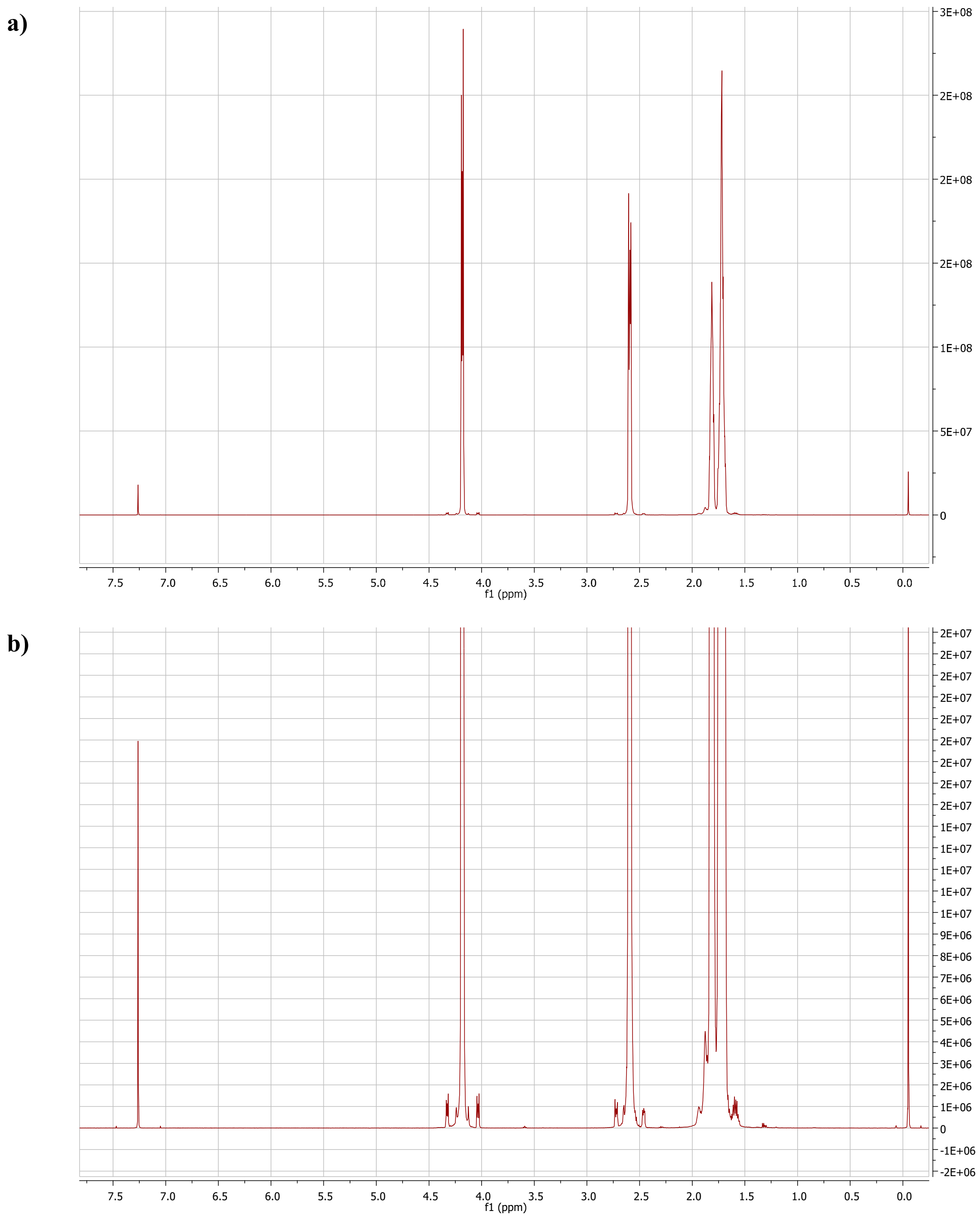

Figure S25. *Caprolactone monomer ^{1}H NMR spectrum;* ***a)*** *standard zoom,* ***b)*** *highly amplified.*

6. FT-IR spectra of obtained PCL-HPPA polymers, PCL linear and monomer.

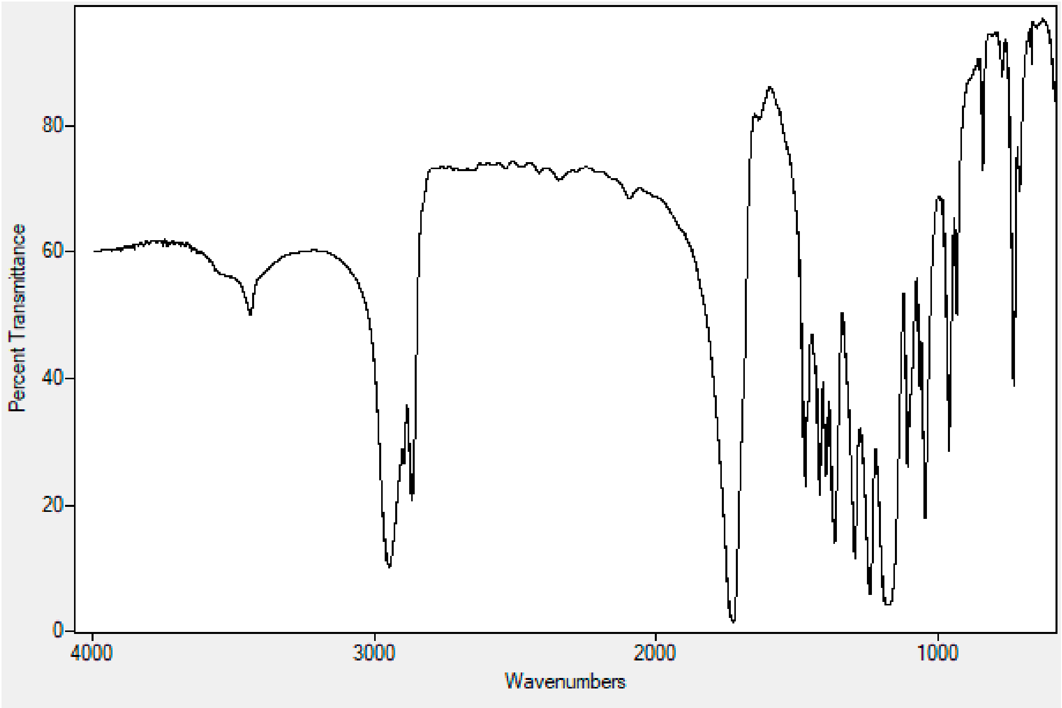

Figure S26. *PCL-linear FTIR spectra*

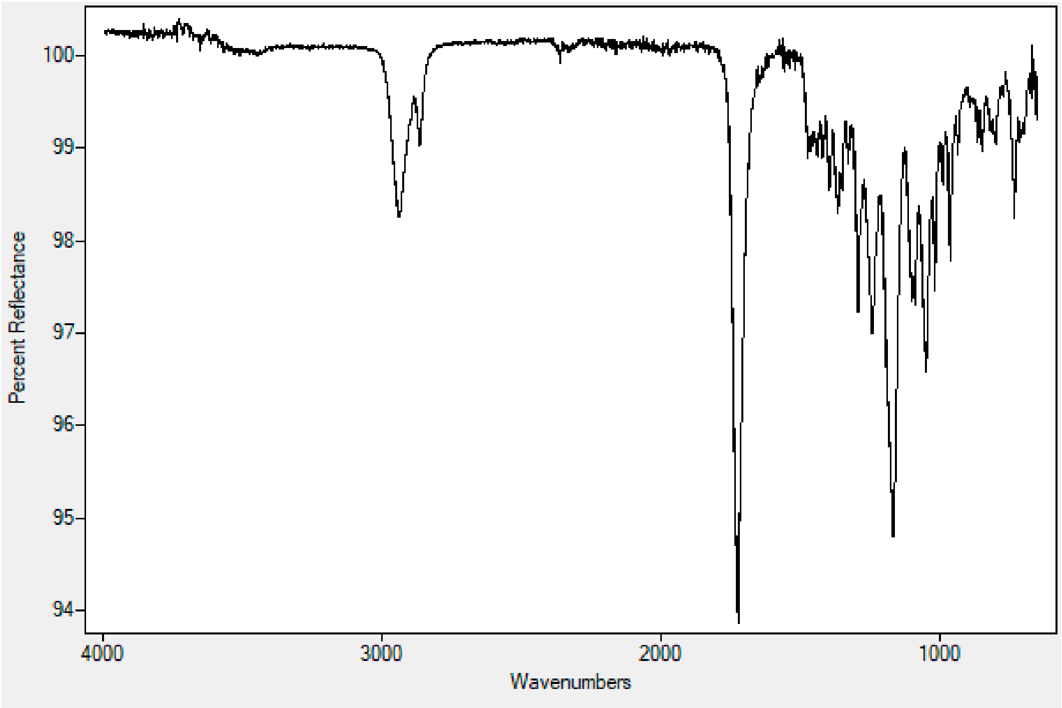

Figure S27. *PCL-0.033% HPPA FTIR spectra*

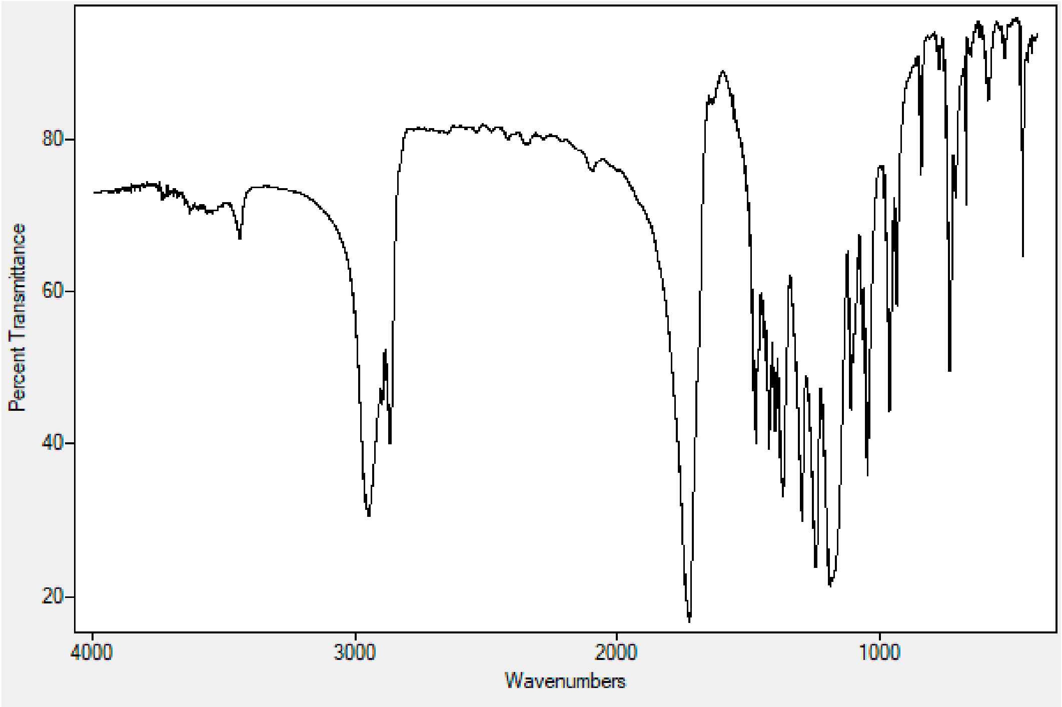

Figure S28. *PCL-0.067% HPPA FTIR spectra*

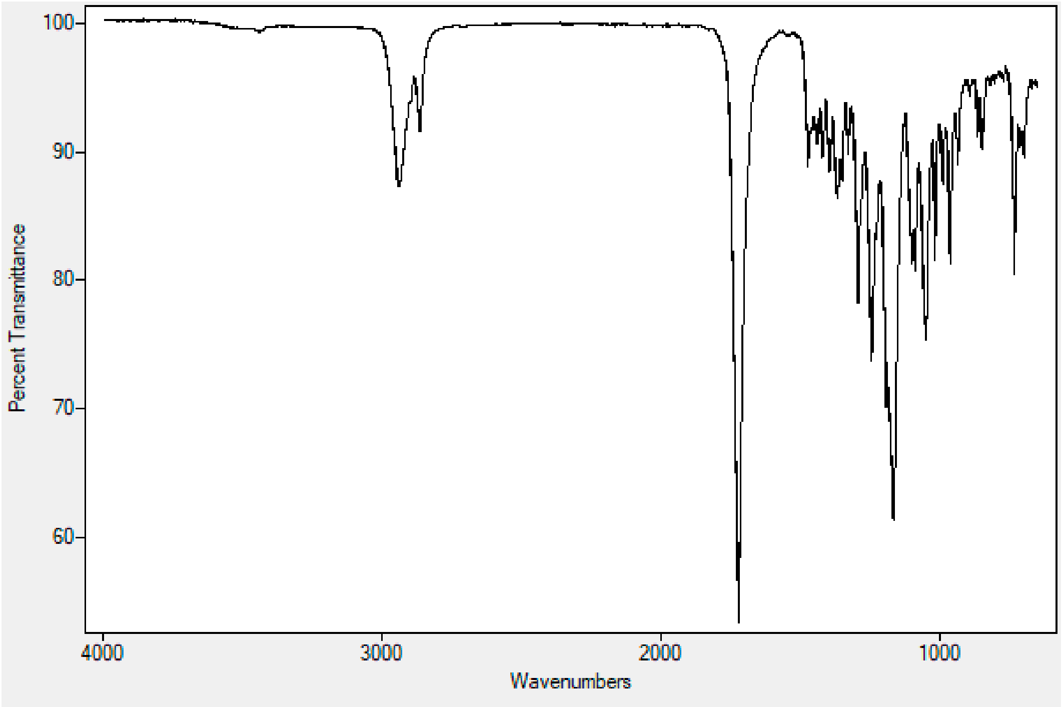

Figure S29. *PCL-0.13% HPPA FTIR spectra*

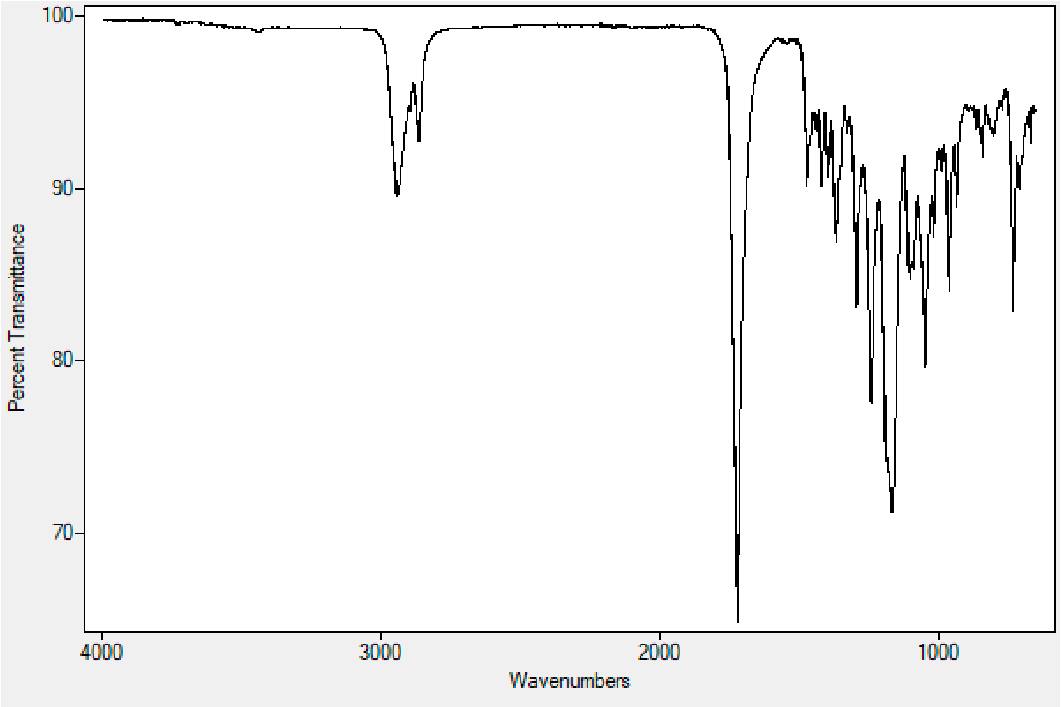

Figure S30. *PCL-0.33% HPPA FTIR spectra*

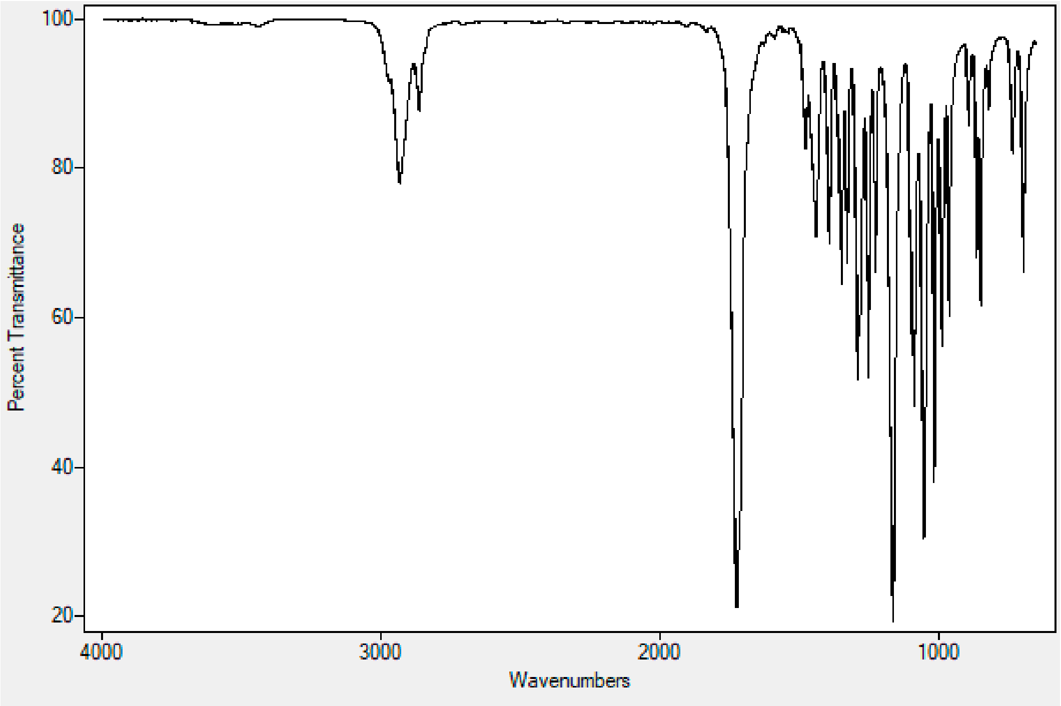

Figure S31. *Caprolactone monomer FTIR spectra*

7. DSC thermograms of obtained PCL-HPPA polymers, PCL linear and monomer.

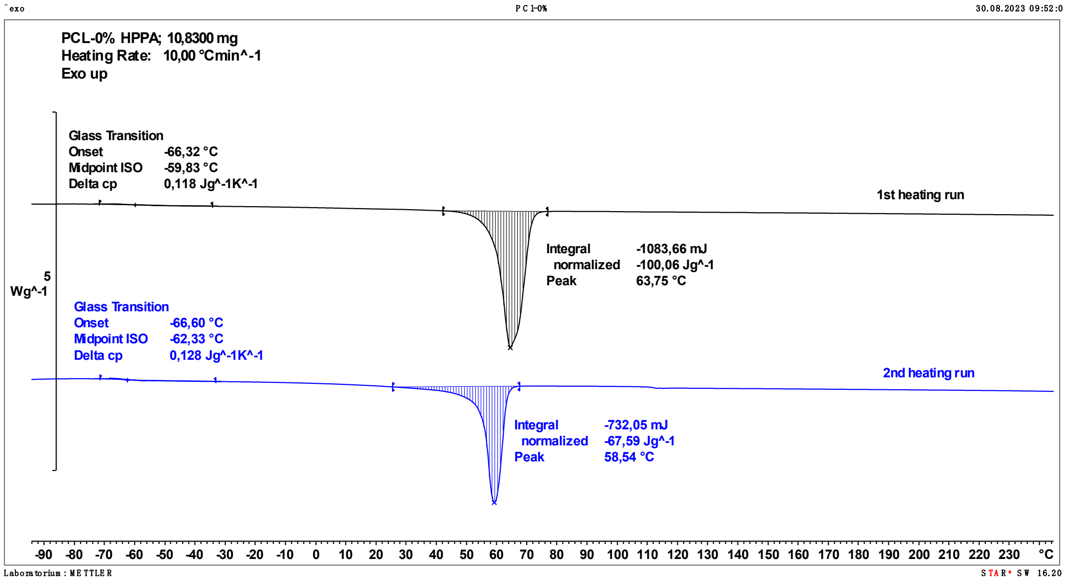

Figure S32. *PCL-linear DSC thermogram*

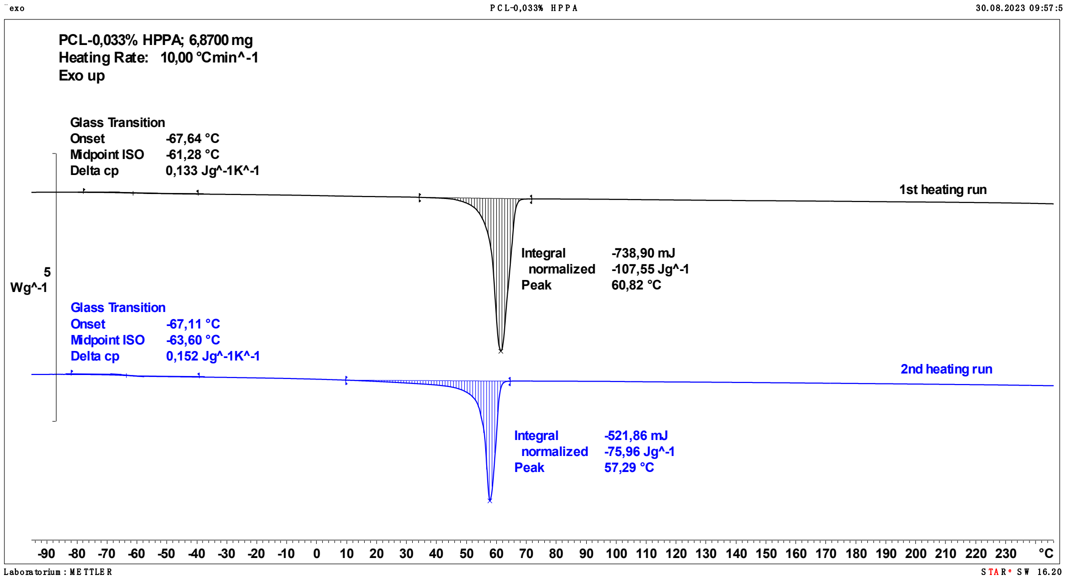

Figure S33. *PCL-0.033% HPPA DSC thermogram*

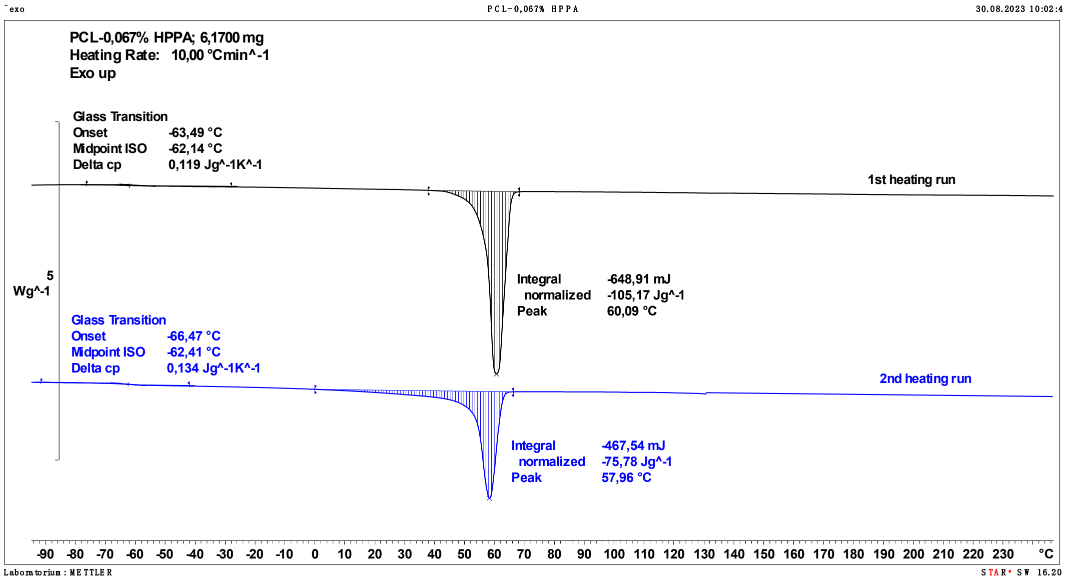

Figure S34. *PCL-0.067% HPPA DSC thermogram*

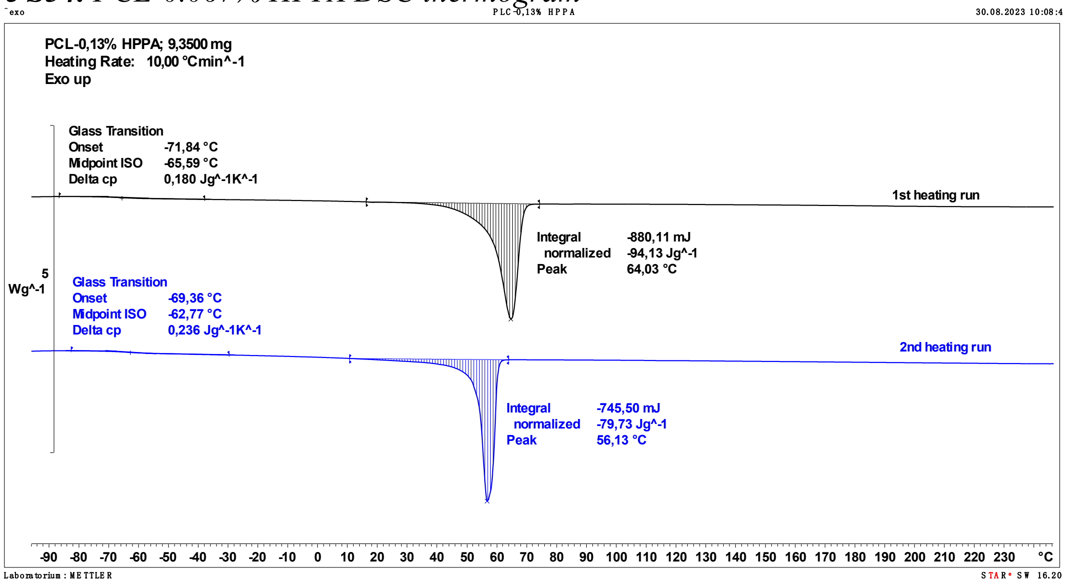

Figure S35. *PCL-0.13% HPPA DSC thermogram*

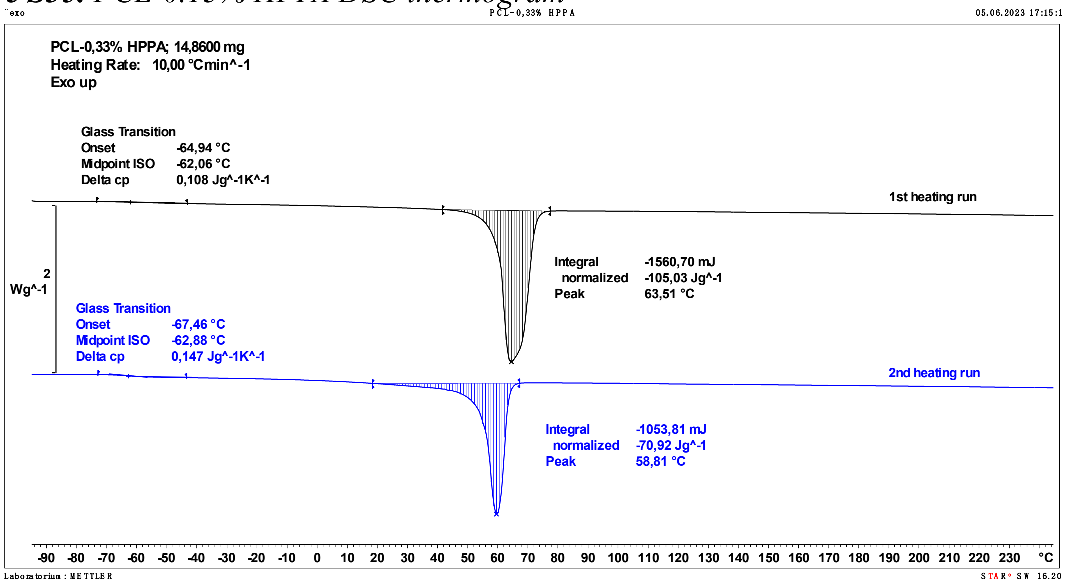

Figure S36. *PCL-0.33% HPPA DSC thermogram*

8. X-ray diffraction (XRD) of obtained PCL-HPPA polymers, PCL linear and monomer.

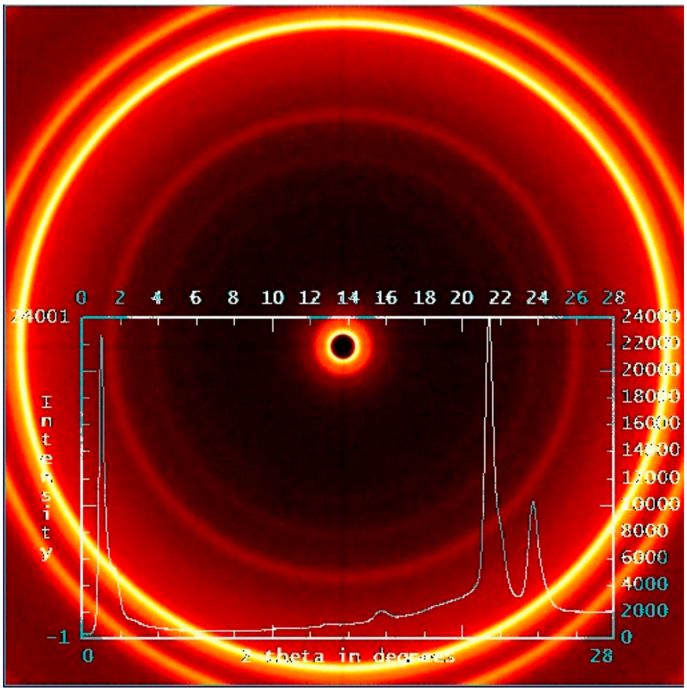

Figure S37. *PCL-linear XRD image*

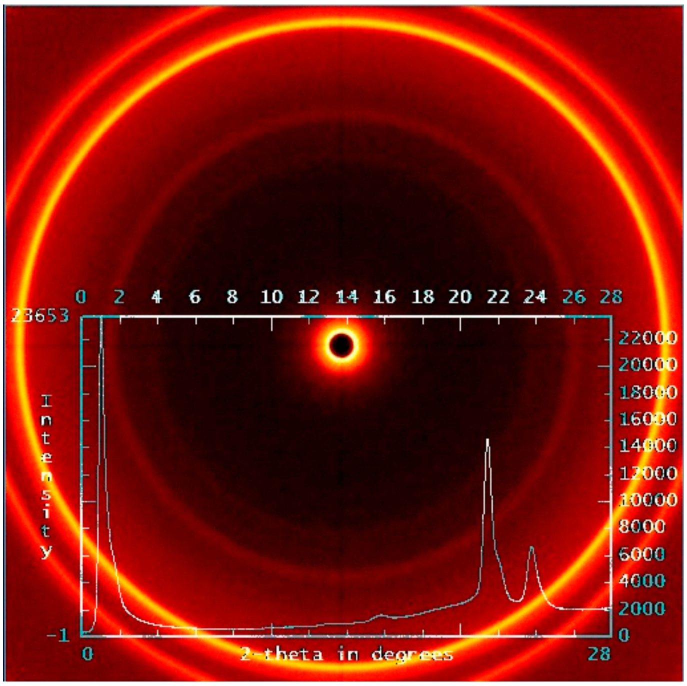

Figure S38. *PCL-0.033% HPPA XRD image*

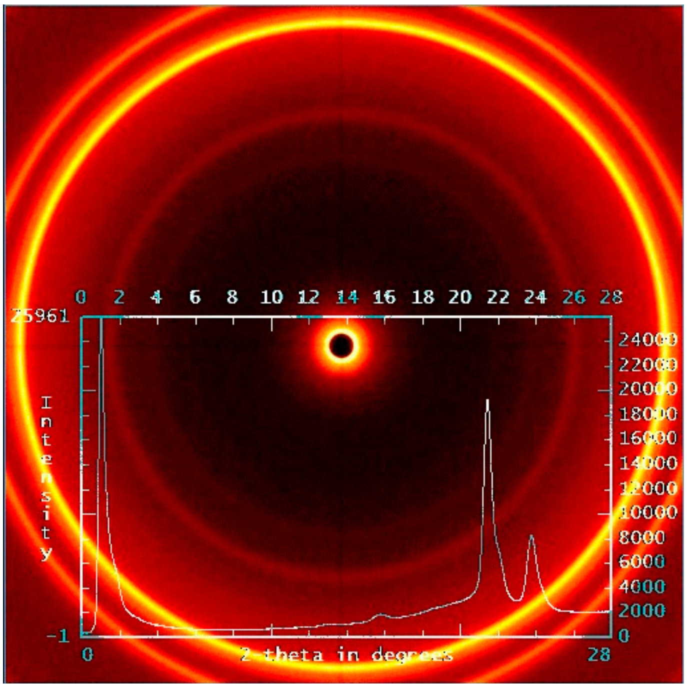

Figure S39. *PCL-0.067% HPPA XRD image*

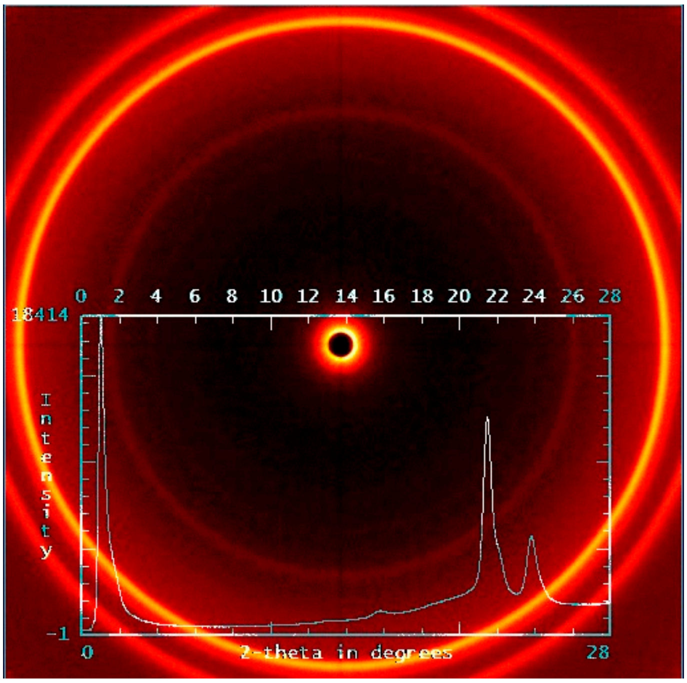

Figure S40. *PCL-0.13% HPPA XRD image*

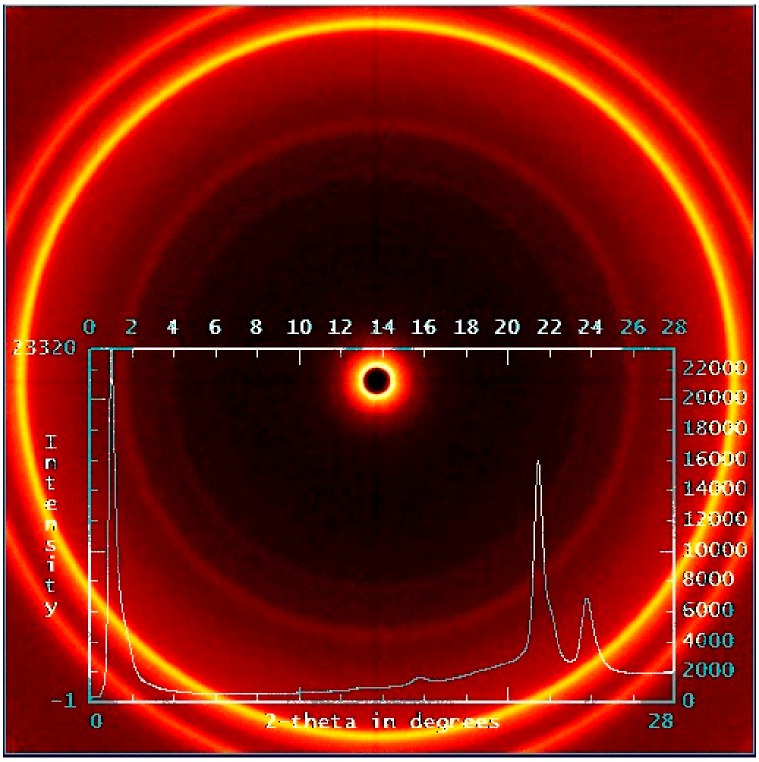

Figure S41. *PCL-0.33% HPPA XRD image*
